# Novel R2R3 MYB transcription factors regulate anthocyanin synthesis in *Aubergine* tomato plants

**DOI:** 10.1186/s12870-023-04153-7

**Published:** 2023-03-20

**Authors:** Jacopo Menconi, Pierdomenico Perata, Silvia Gonzali

**Affiliations:** PlantLab, Center of Plant Sciences, Scuola Superiore Sant’Anna, Piazza Martiri della Libertà 33, Pisa, 56127 Italy

**Keywords:** *Solanum lycopersicum*, Tomato, *Abg*, Anthocyanins, R2R3 MYB, Alternative splicing

## Abstract

**Background:**

A high content in anthocyanins, for their health beneficial properties, represents an added value for fruits and vegetables. Tomato (*Solanum lycopersicum*) is one of the most consumed vegetables worldwide and is rich in vitamins and carotenoids. In recent years, purple-skinned tomatoes, enriched of anthocyanins, were produced recovering allelic variants from wild Solanum species. The molecular basis of the *Anthocyanin fruit* (*Aft*) locus, exploited by breeders to activate the anthocyanin synthesis in tomato epicarp, has been recently identified in the correct splicing of the *R2R3 MYB* gene *AN2like*. *Aubergine* (*Abg*) is a tomato accession which introgressed from *Solanum lycopersicoides* a locus activating the synthesis of anthocyanins in the fruit. The *Abg* locus was mapped in the region of chromosome 10 containing *Aft* and the possibility that *Abg* and *Aft* represented alleles of the same gene was hypothesized.

**Results:**

We dissected the *R2R3 MYB* gene cluster located in the *Abg* genomic introgression and demonstrated that *AN2like* is correctly spliced in *Abg* plants and is expressed in the fruit epicarp. Moreover, its silencing specifically inhibits the anthocyanin synthesis. The *Abg* allele of *AN2like* undergoes alternative splicing and produces two proteins with different activities. Furthermore, in *Abg* the master regulator of the anthocyanin synthesis in tomato vegetative tissues, *AN2*, is very poorly expressed. Finally, a novel *R2R3 MYB* gene was identified: it encodes another positive regulator of the pathway, whose activity was lost in tomato and in its closest relatives.

**Conclusion:**

In this study, we propose that *AN2like* is responsible of the anthocyanin production in *Abg* fruits. Unlike wild type tomato, the *Abg* allele of *AN2like* is active and able to regulate its targets. Furthermore, in *Abg* alternative splicing leads to two forms of *AN2like* with different activities, likely representing a novel type of regulation of anthocyanin synthesis in tomato.

**Supplementary Information:**

The online version contains supplementary material available at 10.1186/s12870-023-04153-7.

## Background

The large chemical diversity of the secondary metabolites existing in plants is made possible by a set of enzymes acting in their biosynthetic pathways and encoded by structural genes transcriptionally regulated in response to environmental or developmental cues. The transcription factors (TFs) modulating these processes are encoded by regulatory genes often belonging to multigene families and arranged in genomes as tandem repeats, deriving from duplication events. In higher plants, indeed, gene duplication represented an important mechanism in the diversification of the secondary metabolism [1, 2].

The R2R3 MYB constitutes one of the plant largest groups of TFs: they belong to the family of MYB (MYeloBlastosis) proteins, ubiquitous in the eukaryotic organisms, and exhibit the conserved MYB DNA-binding domain consisting of two imperfect repeats (R2 and R3) of about 50 amino acids. These TFs have been associated with a multiplicity of different processes, including regulation of cell identity and fate, control of primary and secondary metabolism, and response to biotic and abiotic stresses [3, 4].

One of the best characterized biosynthetic pathways regulated by R2R3 MYBs is the synthesis of anthocyanins, a class of secondary metabolites playing essential roles in defense mechanisms and flower and fruit colors [5]. Anthocyanins are soluble polyphenolic pigments belonging to the group of flavonoids, synthesized through a specific branch of the phenylpropanoid pathway and accumulated in vacuoles of plant cells [6]. The structural genes of this pathway are conventionally divided in early biosynthetic genes and late biosynthetic genes (LBGs), being the early genes involved in the first common enzymatic steps of the flavonoid synthesis and acting the LBGs in the specific final ramifications of the pathway, leading to anthocyanins or proanthocyanidins [7].

In the same species multiple R2R3 MYB TFs can finely regulate the anthocyanin synthesis in different tissues and at different times, acting either in positive or negative ways, and both singularly and taking part to multiprotein MBW complexes where the MYB TF physically interacts with a basic Helix-Loop-Helix (bHLH) factor and a WD40-repeat (WDR) protein [8]. The R2R3 MYB TFs are conventionally divided in subfamilies, according to the presence of specific amino acidic signatures conferring them peculiar properties [3]. In the anthocyanin pathway the positive R2R3 MYB regulators usually belong to the subgroup 6, whereas the negative regulators to the subgroup 4 [9]. When part of a MBW complex, the MYB factor confers target gene specificity to the activity of the complex, and its production is strictly regulated by specific developmental or environmental stimuli. On the other hand, bHLH and WDR partners can be less specific, often regulating more than one biological process [10].

The R2R3 MYB proteins involved in anthocyanin synthesis can be encoded by paralogous genes which often lie in tandem on the same genomic region. In *Arabidopsis thaliana*, for example, four R2R3 MYB TFs (PAP1, PAP2, MYB113 and MYB114), showing a high sequence similarity, can induce anthocyanin synthesis, and three of the relative genes (*PAP2*, *MYB113* and *MYB114*) are tightly linked on chromosome 1 [11]. In petunia (*Petunia* x *hybrida*), four *R2R3 MYB* genes were identified as positive regulators of anthocyanin production in different tissues: *AN2*, *AN4*, *DPL*, and *PHZ* [12, 13]. Except for *AN2*, the other three genes cluster together in the same genomic scaffold. Recently, other three *MYB* genes, *ASR1*, *ASR2* and *ASR3*, have been identified in two petunia wild species (*P. axillaris* and *P. inflate*) arranged in a second cluster, representing a genomic duplication of the first one [14].

Clusters of *R2R3 MYB* genes modulating anthocyanin production are also present in the genome of some cultivated *Solanaceae*. In tomato (*Solanum lycopersicum*), four R2R3 MYB TF-encoding genes, namely *ANT1*, *ANT1like*, *AN2* and *AN2like*, lie on the distal part of the long arm of chromosome 10 [15]. In the tomato accession *Anthocyanin fruit* (*Aft*) the same cluster of *MYB* genes was introgressed from *Solanum chilense* [16] and shows extensive variation in coding and promoter sequences compared to the tomato reference genome [17]. In eggplant (*Solanum melongena*), a major quantitative trait locus, responsible for most of the phenotypic variation in fruit and leaf anthocyanin pigmentation, is in chromosome 10 [18]. Similarly, in wild (*Solanum commersonii*) and cultivated (*Solanum tuberosum*) species of potato, *R2R3 MYB* paralogous genes are in tandem in the same genomic scaffold of chromosome 10 and show sequence variability and functional divergence [19]. Finally, a major region in chromosome 10 containing 12 genes related to the accumulation of anthocyanins in fruits was recently found in pepper [20]. The presence of these clusters of *R2R3 MYB* genes in the same chromosome, often organized in syntenic blocks, indicates a relatively recent divergence of these species from a common ancestor, where the *R2R3 MYB* genes likely expanded by duplication and subsequent diversification.

In tomato the *R2R3 MYB* genes belonging to the cluster in chromosome 10 show highly conserved sequences but also functional divergence [15, 16]. For three of them, *ANT1*, *ANT1like*, and *AN2*, a promoting activity on anthocyanin biosynthesis was demonstrated, with a major role exerted by AN2 in vivo in vegetative tissues [15, 16]. Furthermore, the presence of a splicing mutation in *AN2like* leads to the production of a non-functional TF, preventing the induction of anthocyanin pigmentation in fruits, where this gene is expressed [16, 21]. On the contrary, in *Aft* plants the allele of *AN2like*, derived from *S. chilense*, is functional [16, 21, 22] and allows the production of anthocyanin spots in the fruit peel [23].

A fruit phenotype similar to *Aft* is shown by the *Aubergine* (*Abg*) tomato accession, which carries a genomic introgression from the wild nightshade (*Solanum lycopersicoides*) [24]. This phenotype cosegregates as a monogenic dominant trait with a RAPD marker in the long arm of chromosome 10: this raises the possibility that *Aft* and *Abg* may represent alleles of the same gene [24, 25]. However, the variegated anthocyanin pigmentation shown by *Abg* fruit peel, which can appear spotted, blotched or fully homogeneous even in different fruits of the same plant, is peculiar [24], and the degree of anthocyanin accumulation can be stronger than in *Aft* [26]. In *Abg*, as in *Aft*, the fruit pigmentation is light-dependent, a trait reminiscent of the light-induced strong anthocyanin accumulation shown by the fruits of the wild nightshade, which is native of the Andes region between Peru and Chile, where the high-elevation climate is characterized by very high light intensities [27]. Under adequate light exposition, also *Abg* fruits can show a very intense coloration, resembling the black peel of the “aubergine” (*S. melongena*) fruits, from which the accession took the name [24].

Because of the mutation identified in the gene *AN2like*, the plants of domesticated tomato cannot produce anthocyanin-pigmented fruits. In recent years, however, the growing interest in the dietary effects of anthocyanins in human health [5] led to the production by genetic engineering or breeding of purple tomatoes, which can synthesize and accumulate anthocyanins in addition to carotenoids [28]. Breeding exploited the possibility to cross *S. lycopersicum* with some wild Solanum species which produce anthocyanins in their fruit peel, as the species *S. chilense* and *S. lycopersicoides* above mentioned [29].

The strong interest in the anthocyanin-enriched tomatoes and the necessity to further improve their qualities prompted us to carry out the molecular characterization of the *Abg* accession, known for a long time but never studied in detail, with the aim to ascertain the genetic basis of its phenotype and to possibly find out new sources of genetic variability for tomato breeding.

## Results

### The ***Abg*** plants show a peculiar anthocyanin phenotype

**Fig. 1:**
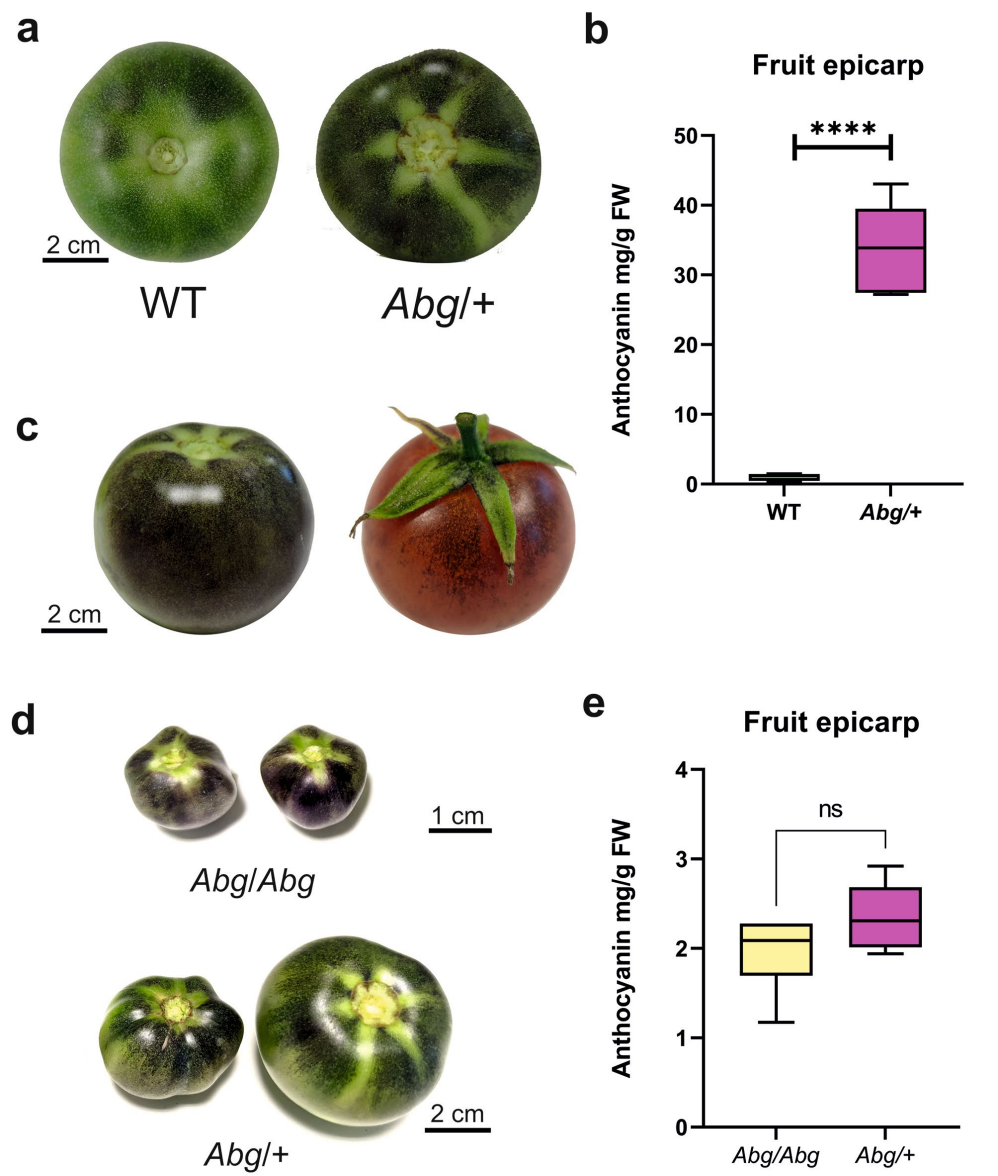
Phenotype of *Abg* fruits. **a** Tomatoes at the mature green stage from cv. Ailsa Craig (wild type, WT) (left), and *Abg* heterozygous genotype (*Abg*/+) (right). **b** Anthocyanin content measured in the fruit epicarp of WT and *Abg*/+ tomatoes at the mature green stage. Anthocyanins are expressed in mg petunidin-3-(p-coumaroyl-rutinoside)-5-glucoside g^− 1^ fresh weight (FW). Data are means of six biological replicates. Unpaired t-test was carried out and **** asterisks indicate significant difference (P ≤ 0.0001). **c** Different patterns of anthocyanin accumulation in *Abg* fruits at the mature green and red stages. **d** Fruit morphology of *Abg*/*Abg* and *Abg*/+ plants. **e** Anthocyanin content measured in the fruit peel of *Abg*/*Abg* and *Abg*/+ tomatoes at the mature green stage. Anthocyanins are expressed in mg petunidin-3-(p-coumaroyl-rutinoside)-5-glucoside g^− 1^ FW. Data are means of six biological replicates. Unpaired t-test (P ≤ 0.05) was carried out. Photographs are from the authors

The *Abg* tomato line was originally selected for its fruit phenotype [24]: when fruits are light-exposed, they can indeed synthesize anthocyanins in the upper epicarp, accumulating them in diverse patterns, from spotted to totally flushed purple motifs, from the mature green stage on, and anthocyanins persist in the red ripe fruit peel (Fig. 1a-c).

The *Abg* phenotype is dominant and the anthocyanin pigmentation in fruits is similar in homozygous (*Abg*/*Abg*) and heterozygous (*Abg*/+) plants (Fig. 1d, e). Morphology, instead, is very different, with the homozygous fruits which expand less, reaching final sizes lower than those of the heterozygous fruits, and are seedless and often malformed (Fig. 1d).

**Fig. 2:**
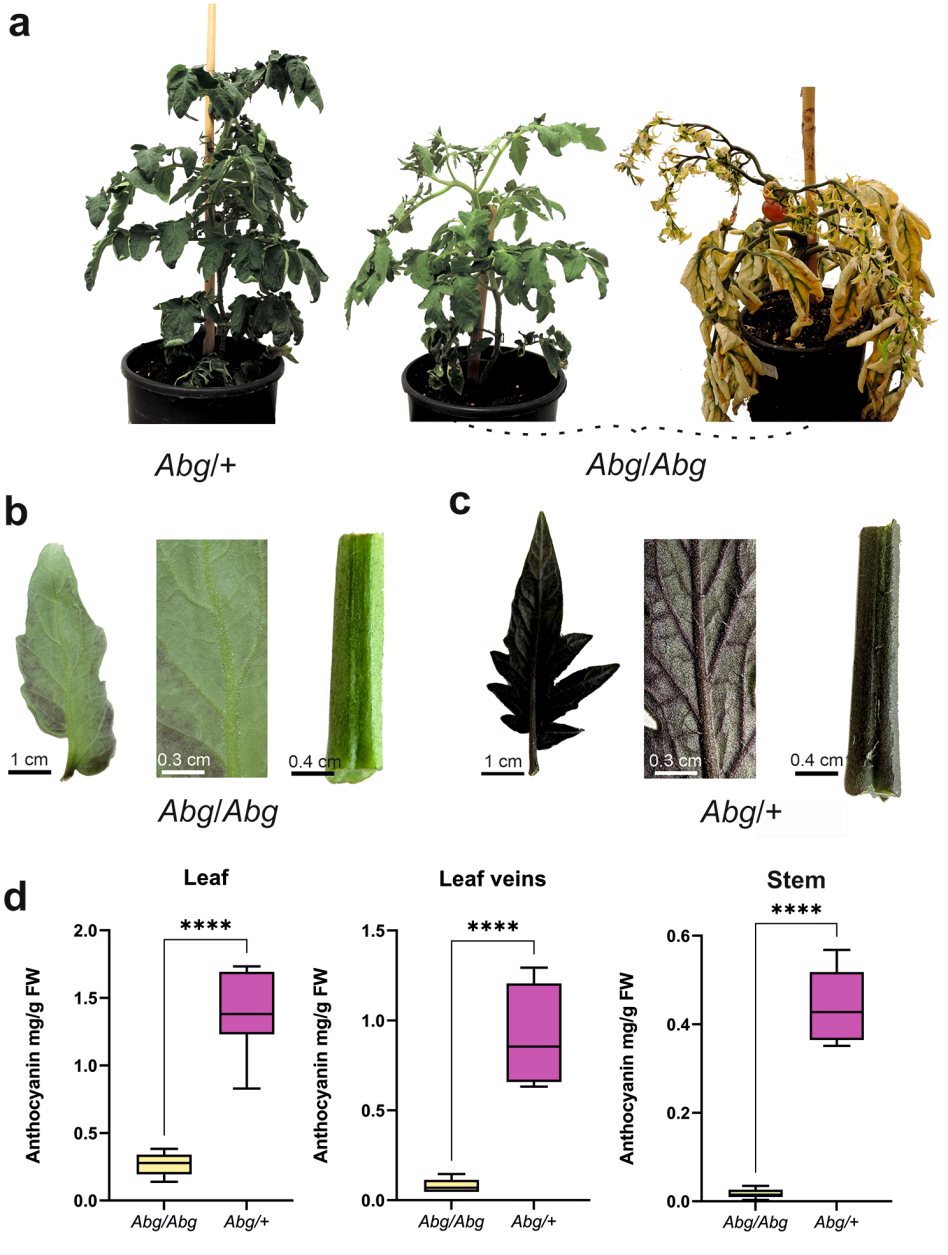
Anthocyanin phenotype of *Abg* plants. **a** Growth habits of 50-day-old *Abg* heterozygous (*Abg*/+) plants (left), 50-day-old *Abg* homozygous (*Abg*/*Abg*) plants (centre), and three-month-old *Abg*/*Abg* plants (right). Leaf abaxial side, magnification of the leaf veins in the abaxial side, and internode of the stem of an *Abg*/*Abg* (**b**) and an *Abg*/+ (**c**) plant. **d** Anthocyanin content measured in the leaf, leaf veins and stem of 50-day-old *Abg*/*Abg* and *Abg*/+ plants. Anthocyanins are expressed in mg petunidin-3-(p-coumaroyl-rutinoside)-5-glucoside g^− 1^ fresh weight (FW). Data are means of six biological replicates. Unpaired t-test was carried out and **** asterisks indicate significant difference (P ≤ 0.0001). Photographs are from the authors

The growth habits of the two genotypes are also quite different: both types of plants grow and develop normally in the first weeks after germination, but, later, the homozygous plants start to yellow, while producing flowers and fruits (Fig. 2a). The yellow leaves tend to roll and curl, starting from the oldest and moving to the youngest. When are still green, the homozygous plants show a very pale colour in their leaves, stems, and leaf veins compared to the heterozygotes (Fig. 2b, c); this is confirmed by the low levels of anthocyanins measured in these tissues (Fig. 2d). Whereas leaves can slowly accumulate anthocyanins over time, leaf veins and stems remain pale green (Fig. 2b).

### Analysis of ***Abg R2R3 MYB*** genes involved in anthocyanin synthesis

Given the peculiar phenotype in fruits and vegetative tissues, each of the *R2R3 MYB* genes located in the distal part of the long arm of chromosome 10 (where the genomic introgression from *S. lycopersicoides* was mapped [24, 25]) and known to be involved in regulation of the anthocyanin biosynthetic pathway in tomato [15, 16] was cloned and sequenced in *Abg* homozygous plants. The corresponding wild type (WT) tomato sequences [17], and the genomic sequences recently assembled for *S. lycopersicoides* [30] were used for comparison. Four genes orthologous to the four *R2R3 MYB* genes of tomato were identified (Fig. S1–S4). They showed several polynucleotide and single nucleotide polymorphisms (SNPs) compared to WT, and most of them, as expected, were also present in the wild nightshade corresponding genomic sequences (Fig. S5-S8).

Unexpectedly, a fifth and novel gene, very similar to the previous four, was additionally identified in *Abg*, and its presence was confirmed in *S. lycopersicoides* chromosome 10, contiguous to the other four *R2R3 MYB* genes (Fig. S9). Due to its similarity with the gene *MYB113* present in other *Solanaceae* (Fig. S10-S11), we called it *MYB113*^*Abg*^. A Blast search in the tomato genome, using *MYB113*^*Abg*^ as a query, revealed the existence of a similar sequence in the very distal part of chromosome 10, 8.2 kb far from the genomic sequence of *AN2like* (*Solyc10g086290*) (Fig. S12a, c). We cloned and sequenced this tomato genomic region: it resulted shorter than the 1340 nucleotide-*MYB113*^*Abg*^ open reading frame, matching its first 457 and last 540 nucleotides (Fig. S12b-S13). The first part of the tomato sequence (in forward orientation) well aligned with *MYB113*^*Abg*^, but the corresponding putative transcript contains a premature stop codon (Fig. S12b). This sequence is not annotated as a transcript in the tomato genomic database. On the contrary, from the final part of the genomic sequence of tomato aligning with *MYB113*^*Abg*^, in reverse orientation a putative cDNA, corresponding to the *Solyc10g086300.1.1* transcript, is annotated as “Unknown protein” (Fig. S12d, e).

Being not known the exact dimension of the introgressed region of *S. lycopersicoides* in *Abg*, we cloned another *R2R3 MYB* gene, *THM27*(*Solyc10g055410*), located in the long arm of chromosome 10 not far from the other five *R2R3 MYB* genes above described (Fig. S14a). Indeed, the THM27^WT^ protein shows some structural features which would indicate a possible involvement in the flavonoid metabolism as a negative regulator: a bHLH interaction motif in the R3 domain and an “ethylene-responsive element binding factor-associated amphiphilic repression” (EAR) motif in the C-terminal region (Fig. S14b). Again, *THM27*^*Abg*^ shared with the corresponding sequence of *S. lycopersicoides* most of the nucleotide polymorphisms towards the *THM27*^*WT*^ gene (Fig. S15), indicating its origin from the wild parental species.

To easily assess the genotype of the *Abg* plants, which were phenotypically very similar in the early stages of development, a Cleaved Amplified Polymorphic Sequence marker for the gene *AN2like*, allowing discrimination among wild type (+/+), *Abg*/+ and *Abg*/*Abg* genotypes, was designed (Fig. S17).

**Fig. 3:**
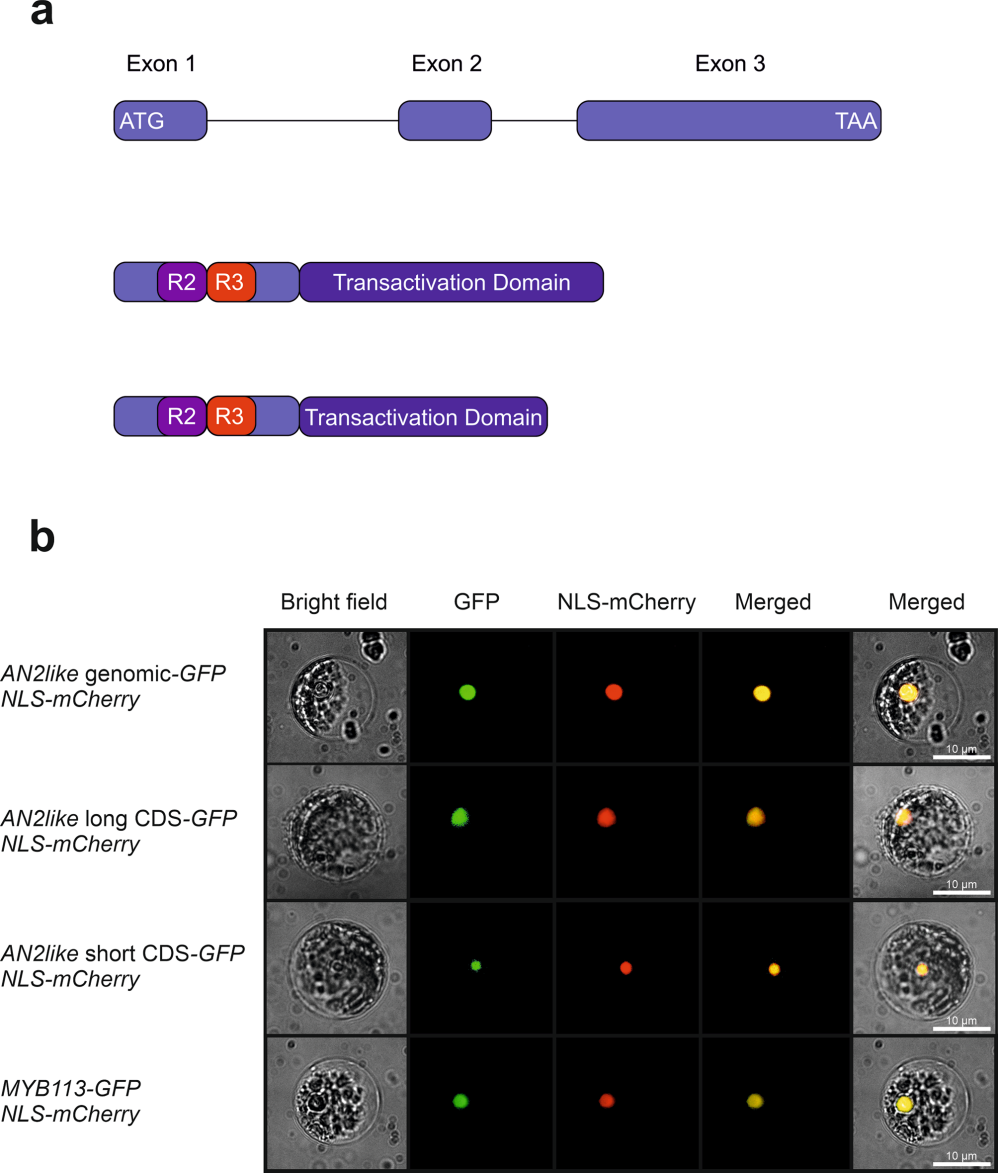
Alternative splicing of *AN2like*^*Abg*^ and cellular localization of the novel *Abg* R2R3 MYB proteins. **a** Structure of the gene *AN2like* of *Abg* plants, showing the start and stop codons, exons represented by boxes and introns represented by lines, and of the long and the short transcripts produced from the same gene with the positions of R2 and R3 MYB domains and of the transactivation domain. The different parts of the transcripts are proportionally scaled. **b** Cellular localization of total AN2like^Abg^-GFP, short AN2like^Abg^-GFP, long AN2like^Abg^-GFP and MYB113^Abg^-GFP fusion proteins, co-expressed with the Nuclear-Localization Signal (NLS)-mCherry protein in tomato protoplasts. Bright field, GFP, NLS-mCherry, and merged GFP + NLS-mCherry or bright field + GFP + NLS-mCherry images of protoplasts are shown

Alternative splicing and splicing mutations can crucially affect the activity of R2R3 MYB TFs involved in the regulation of anthocyanin synthesis [16, 21]. For this reason, we first inferred the cds of the *Abg MYB* genes identified in chromosome 10 from the relative gene sequences and predicted the corresponding polypeptide sequences, without finding out major mutations expected to affect their activity (Fig. S16). Then we cloned the transcripts of the novel *Abg* alleles of *AN2like* and *MYB113* from fruits and leaves, respectively, of *Abg* homozygous plants and compared them with the cds bioinformatically predicted. Whereas the cds of *MYB113*^*Abg*^ was identical with what expected from the canonical splicing of its gene (Fig. S9a), two different *AN2like* transcripts were isolated from the fruit epicarp. One, deriving from the combination of three exons (Fig. S19a, b), corresponded to the canonically spliced cds and well aligned with the *AN2like* mRNA expressed in *Aft* fruits (Fig. 3a; Fig. S19c) [16, 21]. The other transcript, shorter for the presence of an 81-nt deletion in the third exon, which was spliced as an additional intron, maintained the frameshift in the downstream sequence, thus producing a putative polypeptide 27 amino acids shorter than the one translated from the longer transcript (Fig. 3a; Fig. S19b, d).

### Functional analysis of the ***Abg*** R2R3 MYB proteins

To firstly ascertain their possible activities, we analysed the subcellular localization of AN2like^Abg^ and MYB113^Abg^ proteins. To do that, their genomic sequences fused with the *GFP* marker were co-transfected with the *Nuclear Localization Signal-mCherry* sequence in tomato protoplasts. As shown in Fig. 3b, both AN2like^Abg^ and MYB113^Abg^ proteins resulted clearly localized in the cell nuclei. With the same strategy, we checked the cellular localization of the two different cds produced from *AN2like*^*Abg*^, finding that both resulted nucleus-localized (Fig. 3b).

Then, in order to verify whether the R2R3 MYB proteins from the *Abg* locus were functional as TFs, a transactivation assay was carried out. The promoter of the *Dihydroflavonol 4-Reductase* (*DFR)* LBG, encoding the enzyme catalysing the first committed step in plant anthocyanin synthesis [6], driving the firefly *luciferase* reporter gene, was transfected in tomato protoplasts. At the same time, each single *AN2*^*Abg*^, *ANT1*^*Abg*^, *ANT1like*^*Abg*^, *AN2like*^*Abg*^ and *MYB113*^*Abg*^ gene sequence, in combination with *AN1*, encoding the bHLH partner taking part to the MBW complex of tomato [31], were co-expressed as effectors in the protoplasts. The corresponding WT and *Aft R2R3 MYB* alleles [16] were used for comparison.

**Fig. 4:**
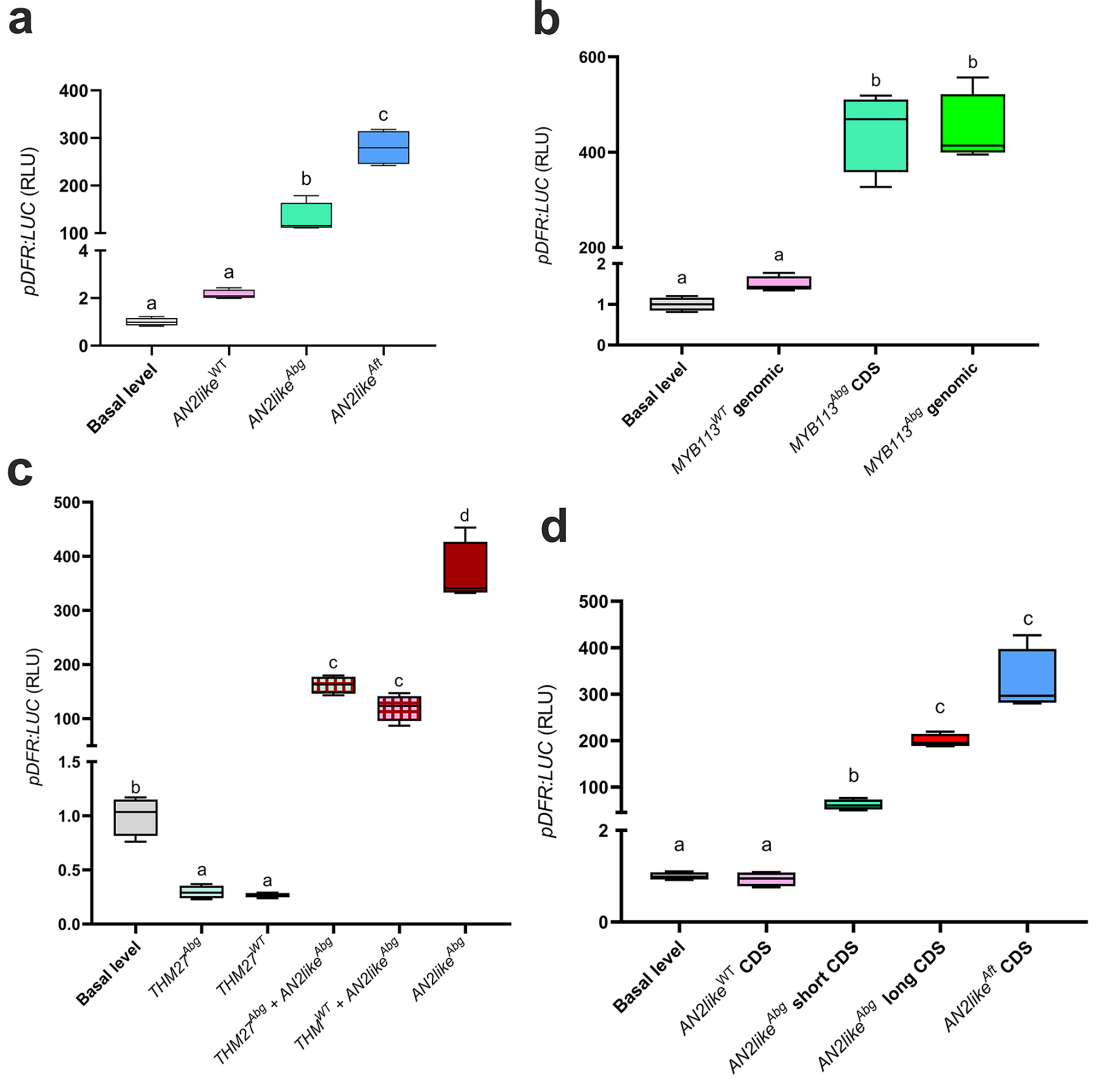
Transactivation activity of the novel *Abg* R2R3 MYB proteins in tomato protoplasts. *DFR* promoter driving firefly *luciferase* gene was transactivated by effector plasmids containing: the *AN2like* genomic sequences from wild type (WT), *Abg* or *Aft* plants **a**; the *MYB113* genomic sequences from WT and *Abg* plants and the *MYB113* cds from *Abg* **b**; the *THM27* genomic sequences from WT and *Abg* plants singularly and in combination with the *AN2like* genomic sequence from *Abg*, and the single *AN2like* genomic sequence from *Abg* **c**; and the *AN2like* cds from WT, *Abg* (both the short and the long transcripts) or *Aft* plants **d**. In all the transactivation assays, the effector *R2R3 MYB* plasmids were co-expressed with the effector plasmid containing the bHLH factor *AN1*. Data are expressed as relative luciferase activity (RLU) (Firefly_Luc/Renilla_Luc) with the value of the *DFR* promoter basal level set to 1 and are means of four biological replicates. One-way ANOVA with Tukey’s HSD post hoc test was performed. Different letters indicate significant differences at P ≤ 0.05

We found that all the *Abg* MYB proteins in the presence of the bHLH partner could transactivate the *DFR* promoter. Moreover, whereas the *AN2*^*Abg*^, *ANT1*^*Abg*^, and *ANT1like*^*Abg*^ sequences showed an efficiency very similar to the orthologous WT and *Aft* alleles (Fig. S18a-c), AN2like^Abg^ transactivated the *DFR* promoter with a little lower efficiency than AN2like^Aft^ (Fig. 4a). On the contrary, and as already known [16], the *AN2like*^*WT*^ allele did not produce a TF able to activate the *DFR* promoter (Fig. 4a).

MYB113^Abg^, differently to the corresponding WT protein, resulted functional in the transactivation assay, giving consistency to a putative role of R2R3 MYB TF able to induce anthocyanin synthesis (Fig. 4b).

Finally, THM27^Abg^, expressed in protoplasts, behaved as an anthocyanin negative regulatory protein because it could repress the activation of the *DFR* promoter induced by the co-expression of AN2like^Abg^ and AN1 (Fig. 4c). In this case, however, also the *THM27*^*WT*^ allele was functional, and the relative protein repressed the activation of the *DFR* promoter with a similar efficiency (Fig. 4c).

**Fig. 5:**
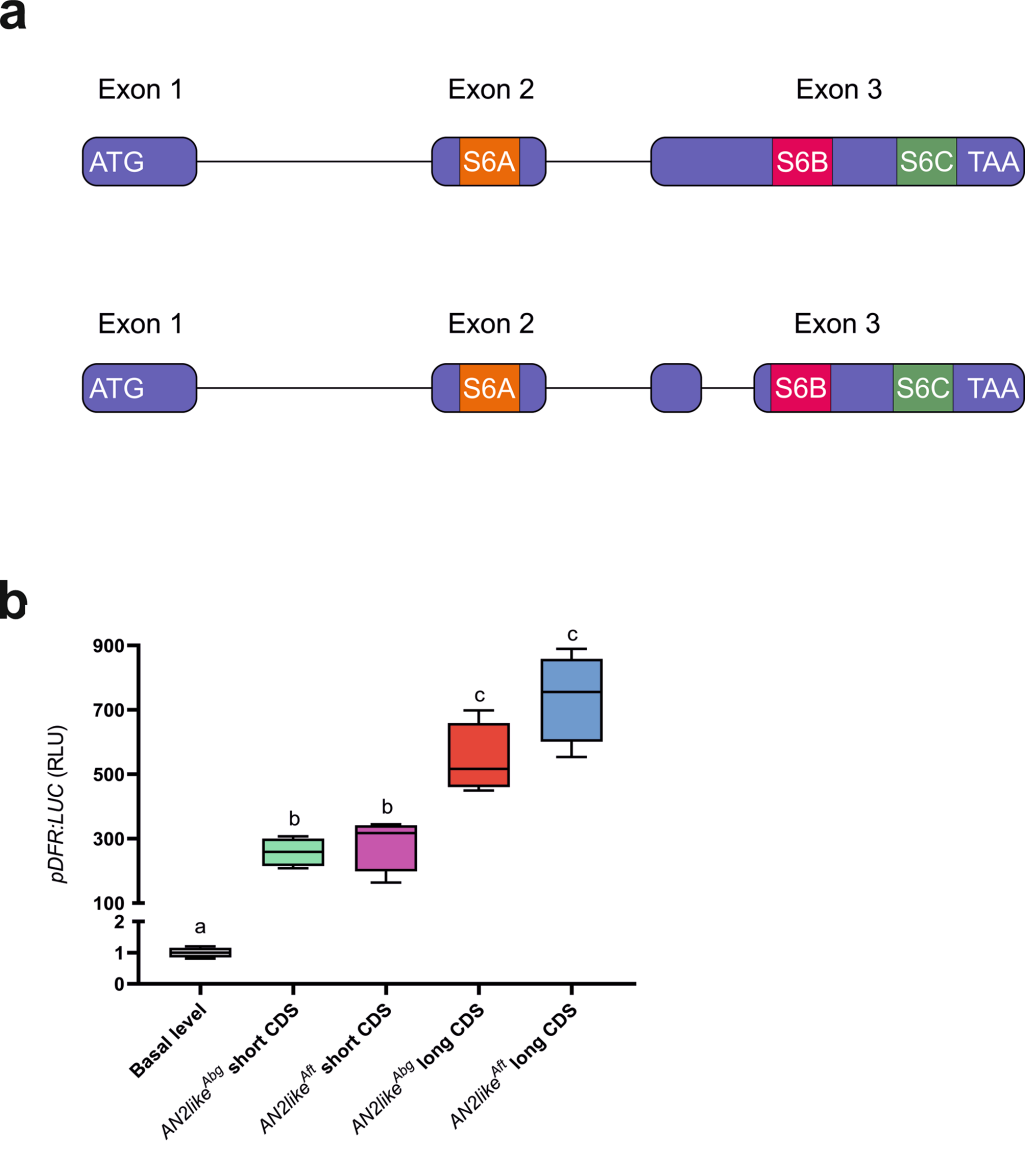
Conserved domains in the *AN2like*^*Abg*^ transcripts and analysis of the activity of the corresponding proteins. **a** Structure of *AN2like*^*Abg*^ showing the three exons and the two introns in the pre-mRNA giving origin to the long transcript (up), and the three introns and the four exons in the pre-mRNA giving origin to short transcript (down). ATG and TAA start and stop codons, S6A, S6B and S6C domains [47] are shown. The different parts of the transcripts are proportionally scaled. **b** Transactivation of the *DFR* promoter driving firefly *luciferase* in protoplasts by effector plasmids containing the long and short transcripts of *AN2like* produced from the genes of *Abg* or *Anthocyanin fruit* (*Aft*) plants. The short *Aft* transcript was produced artificially

Interestingly, in the transactivation assay with the *DFR* promoter, both the *AN2like*^*Abg*^ transcripts resulted functional when co-expressed with *AN1* (Fig. 4d), but the short transcript was less efficient than the long one (Fig. 4d). Thus, to verify if the deletion in the C-terminal part of the polypeptide, due to the splicing of the 81-nt additional intron adjacent to some conserved functional domains (Fig. 5a), was responsible of the lower efficiency, a corresponding 81-nt deletion was produced by mutagenesis in the *AN2like*^*Aft*^ cds. The transactivation assay with the *DFR* promoter was then carried out, and, as observed with the *Abg* transcripts, the *Aft* shorter cds resulted a less efficient effector than the longer one, and the *Abg* and *Aft* short *AN2like* cds resulted quite similar in their transactivation efficiency (Fig. 5b).

**Fig. 6:**
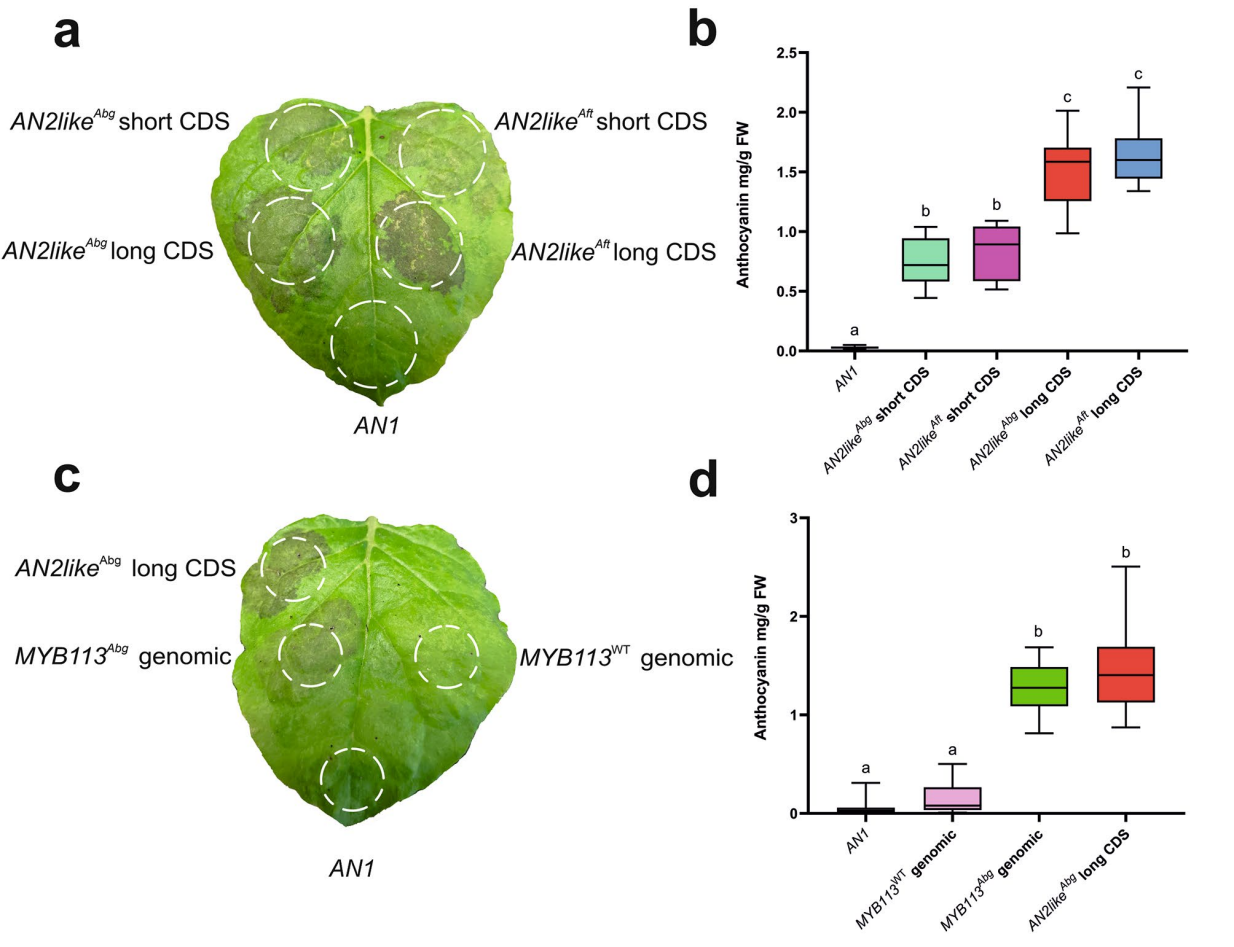
Functional in vivo analyses of AN2like^Abg^ and MYB113^Abg^. **a** Anthocyanin accumulation in leaves of *Nicotiana benthamiana* agroinfiltrated with plasmids containing the *AN2like* long and short transcript sequences of *Abg* or *Aft* co-expressed with the effector plasmid containing the bHLH factor *AN1*, also used alone as negative control. White dotted circles indicate the agroinfiltrated areas. **b** Quantification of the anthocyanins produced in the different areas of the leaves agroinfiltrated as described in a. **c** Anthocyanin accumulation in *N. benthamiana* leaves agroinfiltrated with plasmids containing the *MYB113* genomic sequences of *Abg* or wild type (WT) plants co-expressed with the effector plasmid containing the bHLH factor *AN1*, also used alone as negative control. As positive control, the *AN2like*^*Abg*^ long transcript was co-expressed with *AN1*. White dotted circles indicate the agroinfiltrated areas. **d** Quantification of the anthocyanins produced in the different areas of the leaves agroinfiltrated as described in c. In b and d anthocyanins are expressed in mg petunidin-3-(p-coumaroyl rutinoside)-5-glucoside g^− 1^ fresh weight (FW). Data are means of ten biological replicates. One-way ANOVA with Tukey’s HSD post hoc test was performed. Different letters indicate significant differences at P ≤ 0.05. Photographs are from the authors

To complete the functional characterization with an in vivo assay, *Nicotiana benthamiana* leaves were agroinfiltrated with vectors expressing the *AN2like*^*Abg*^ long or short transcripts or the *MYB113*^*Abg*^ cds compared with the corresponding *Aft* and WT counterparts. The bHLH AN1 was co-expressed with the MYB TFs to produce a partner for the formation of the MBW complex or alone as a negative control. Ectopic anthocyanin synthesis was effectively produced in all the leaves where both AN2like (either *Abg* or *Aft*) and AN1 were expressed, with a higher pigment accumulation in correspondence of the longer transcripts (Fig. 6a), as also demonstrated by the quantification of the anthocyanins isolated from the relative spots on Nicotiana leaves (Fig. 6b).

Anthocyanin production was also induced in leaves where MYB113^Abg^ and AN1 were co-expressed, but not when the *MYB113*^*WT*^ sequence was infiltrated with *AN1* (Fig. 6c, d).

### Expression studies of the ***R2R3 MYB*** genes in ***Abg*** plants

To characterize the process of anthocyanin synthesis *in planta*, an expression analysis of the main genes involved in the regulation of the pathway, including the novel *Abg R2R3 MYB* alleles, was carried out in the main tissues of *Abg* plants. For the analysis, the vegetative tissues were sampled from young plants, when the differences in terms of anthocyanin pigmentation between homozygous and heterozygous genotypes were more visible. Peel was sampled from fruits at the mature green stage, at the beginning of the anthocyanin synthesis.

**Fig. 7:**
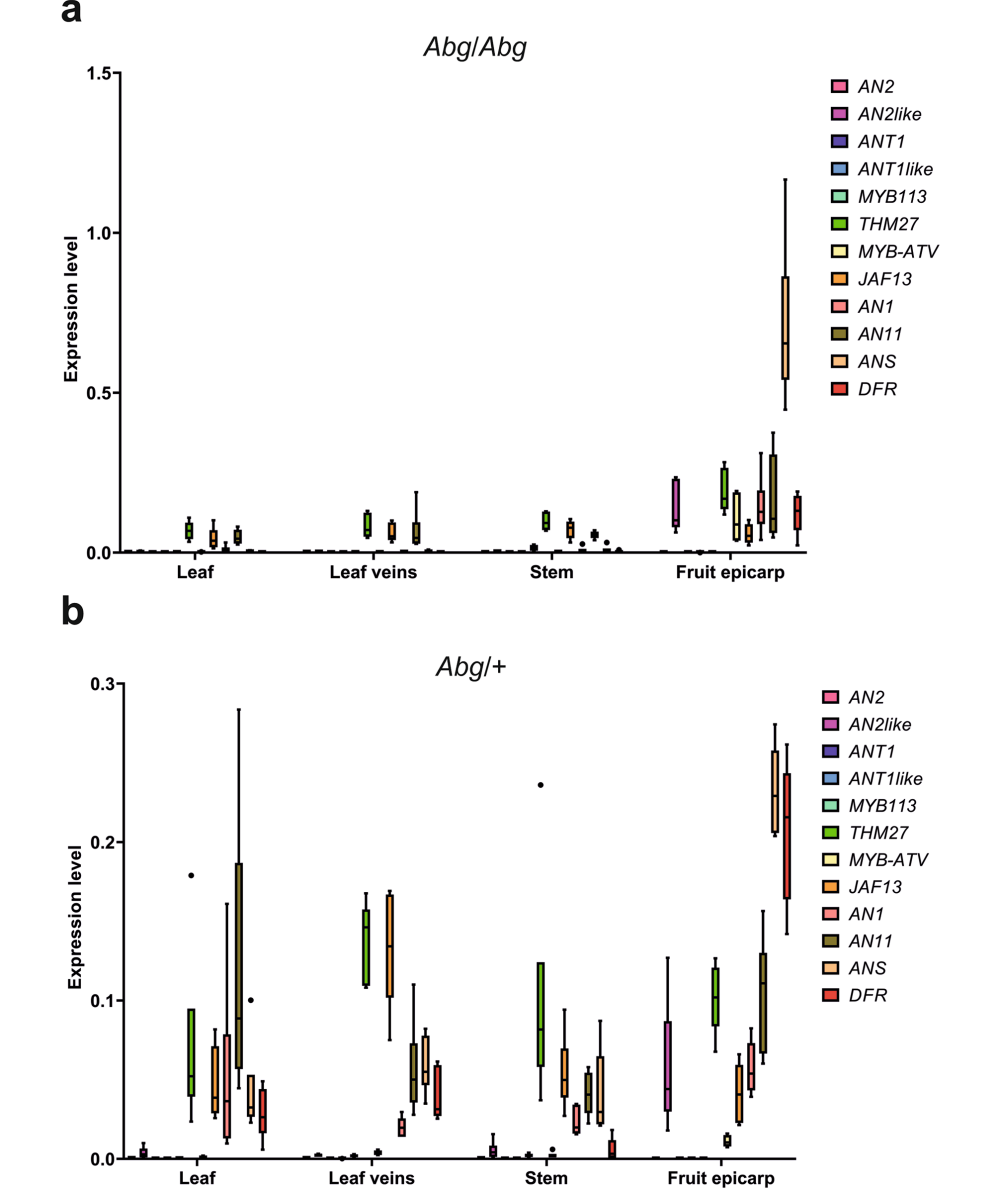
Expression analysis of regulatory and structural genes involved in anthocyanin synthesis in *Abg* plants. Expression levels of the regulatory *R2R3 MYB* (*AN2*, *AN2like*, *ANT1*, *ANT1like*, *MYB113*, *THM27*), *bHLH* (*JAF13*, *AN1*), *WDR* (*AN11*), *R3 MYB* (*MYBATV*), and of the structural late biosynthetic genes *DFR* and *ANS* were analysed by qPCR in leaves, leaf veins, stems and fruit peel from *Abg* homozygous plants (*Abg*/*Abg*) **a** and *Abg* heterozygous plants (*Abg*/+) **b**. Data are means of six biological replicates

By examining the expression levels of the *R2R3 MYB* genes in the vegetative tissues of the homozygous plants, all of them appeared transcribed at barely detectable levels, with *MYB113*^*Abg*^ showing a little bit higher expression in the stems (Fig. 7a). Furthermore, *AN2*^*Abg*^, *ANT1*^*Abg*^, *ANT1like*^*Abg*^ and *MYB113*^*Abg*^ resulted almost not transcribed in the fruit peel, where, on the contrary, *AN2like*^*Abg*^ resulted well expressed (Fig. 7a). The analysis of the other regulatory genes of the pathway showed that the bHLH *JAF13* and the WDR *AN11*, considered constitutively expressed in tomato [16], were expressed at similar good levels everywhere, whereas the bHLH *AN1* and the structural LBGs *DFR* and *ANS*, all regulated by the MBW complex and thus generally correlated with the accumulation of anthocyanins [16], resulted significantly expressed only in the fruit peel (Fig. 7a).

In the heterozygous *Abg* plants, the *Abg* alleles of the *R2R3 MYB* genes followed the same trend shown in the *Abg*/*Abg* plants: all of them resulted poorly transcribed in both vegetative tissues and fruits, with the only exception of *AN2like*, whose expression in the fruit epicarp was considerable (Fig. 7b). Also in these plants, *JAF13* and *AN11* were highly expressed in all the tissues, whereas *AN1*, *DFR* and *ANS* were transcribed not only in the fruit peel, as in the homozygotes, but also in the vegetative tissues (Fig. 7b). On the other hand, in the *Abg*/+ plants anthocyanins were accumulated also in leaves and stems (Fig. 2). Noteworthy, the expression of the *AN2*^*WT*^ allele in the heterozygous plants was significantly higher than the corresponding *Abg* allele in all the vegetative organs (Fig. S20a).

In both genotypes, the anthocyanin *R3 MYB* repressor *MYB-ATV*, known to be transcriptionally regulated by the MBW complex [31], basically followed the expression pattern of *AN1* and of the *LBGs*, whereas the novel *R2R3 MYB* repressor *THM27* showed a sustained expression in both the fruit peel and the vegetative tissues (Fig. 7). Remarkably, in the heterozygous plants, no big differences were observed in the expression levels of the two alleles of *THM27*, whereas the *AN2like*^*WT*^ allele was expressed in the fruit epicarp much less than the *Abg* allele (Fig. S20b, c).

Since *AN2like*^*Abg*^ resulted well expressed in the fruit epicarp, the expression levels of its two different transcripts were analysed and compared: the two isoforms were well expressed in the peel of the homozygous and heterozygous fruits, but in both cases the shorter transcript much less than the longer one (Fig. S21).

### Virus Induced Gene silencing of ***AN2like***^***Abg***^ inhibited pigmentation in ***Abg*** fruit peel

**Fig. 8:**
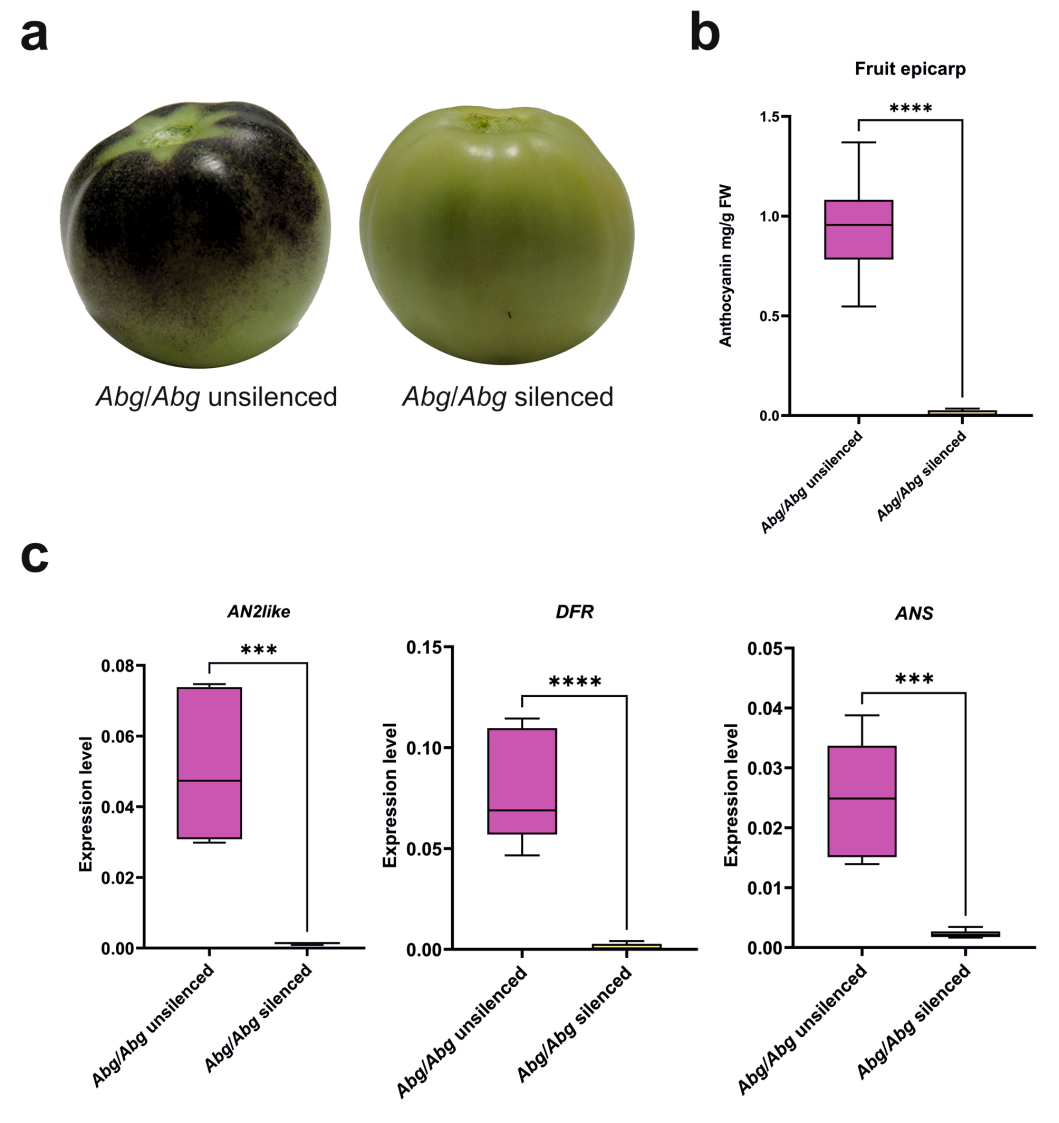
Virus Induced Gene Silencing (VIGS) of *AN2like* in *Abg* fruits. **a** Representative picture of a homozygous *Abg* (*Abg*/*Abg*) fruit that underwent VIGS with the empty TRV2 vector (left) and of a *Abg*/*Abg* fruit that underwent VIGS with the TRV2 vector carrying the silencing construct for the gene *AN2like*^*Abg*^ (right). Both fruits were collected at the mature green stage. **b** Anthocyanin quantification in the fruit peel of unsilenced (VIGS + empty TRV2 vector) and silenced (VIGS + TRV2 vector silencing the gene *AN2like*^*Abg*^) *Abg*/*Abg* fruits. Anthocyanins are expressed in mg petunidin-3-(p-coumaroyl rutinoside)-5-glucoside g^− 1^ fresh weight (FW). Data are means of six biological replicates. Unpaired t-test was carried out and **** asterisks indicate significant difference (P ≤ 0.0001). **c** qPCR analysis of the regulatory *R2R3 MYB* gene *AN2like* and of the structural late biosynthetic genes *DFR* and *ANS* in the peel of *Abg*/*Abg* unsilenced or silenced fruits. Data are means of six biological replicates. Unpaired t-test was carried out and *** asterisks indicate significant difference (P ≤ 0.001) and **** asterisks indicate significant difference (P ≤ 0.0001). Photographs are from the authors

To prove that the expression of *AN2like*^*Abg*^ in the fruit epicarp was indeed responsible of the accumulation of anthocyanins, a VIGS experiment was carried out. Immature green *Abg* fruits were bagged with a light-impermeable cover for two weeks, after which they were agroinfiltrated with a genetic construct designed to silence the two transcripts of *AN2like*^*Abg*^. Four days after the treatment, fruits were uncovered and left on the plant. Within the mature green stage, anthocyanin spots started to appear on the peel of the fruits infiltrated with empty vectors, representing the negative controls, but not in those infiltrated with the gene-silencing genetic cassette (Fig. 8a). Anthocyanin content was then measured on the fruit peel sampled from the negative controls and from the silenced fruits at mature green stage, and qPCR analysis for the genes involved in the anthocyanin pathway was performed, confirming the silencing of *AN2like*^*Abg*^ (but not that of the other *R2R3 MYB* genes – Fig. S22) and of the *LBGs* analysed, as well as the strong reduction in the anthocyanin content, in the silenced fruits (Fig. 8b, c).

### ***MYB113*** is mutated in several Solanum species

Finally, to better characterize the evolution of the novel *MYB113* gene, functional in *Abg* plants but completely disrupted in tomato, sequences orthologous were identified and analysed in other Solanum species and accessions. As shown in Fig. S23a, the large deletion in the second intron of *MYB113* identified in *S. lycopersicum* was also found in very close relatives, such as *S. lycopersicum* var. *cerasiforme* and *S. pimpinellifolium*, and in wild Solanum species located more distant in the evolutionary scale, such as *S. chilense* and *S. pennellii*. Furthermore, in all the species analysed, SNPs or short nucleotide deletions were present in the first or second exon leading to premature stop codons (Fig. S23b). *MYB113*^*Aft*^ showed similar mutations (Fig. S23b). Only one species, *Solanum sitiens*, among those analysed, maintained an intact *MYB113* genomic sequence as *Abg* and *S. lycopersicoides* (Fig. S23b, c).

## Discussion

Domestication and selection, mainly focused on yield increase, strongly reduced over time the genetic diversity of many crops, often at the expense of important disease resistance and nutritional traits [32]. To counteract this trend of “genetic erosion”, modern plant breeding often makes use of wild species which maintained in nature a wide genetic variability from which new alleles or genes can be transferred into the cultivated plants.

Tomato is a horticultural species among the most important worldwide, both for fresh market and processing industry [33]. The global interest towards this plant has recently increased thanks to the recognition of the nutraceutical qualities of its fruits [5, 34]. However, domestication and breeding caused a strong genetic erosion in tomato [35]. Consequently, modern tomato was genetically improved through guided crosses with wild relatives to increase resistance against biotic and abiotic stresses and to recover fruit nutritional quality, taste, and flavour [36]. Colour, important to guide consumer’s choice, is object of great interest and tomato varieties characterized by novel fruit pigmentation patterns have recently appeared on the market.

Novel colours may have been recovered from old or heirloom cultivars, exploiting mutations in the chlorophyll, carotenoid, or flavonoid biosynthetic pathways [28, 37, 38], or may have been created *ex novo*. Purple-skinned tomatoes, enriched of anthocyanins in their epicarp, have been repeatedly obtained in recent years through hybridization with wild Solanum species which, differently from tomato, can synthesize these compounds in their fruits. Allelic variants in positive and negative regulator-encoding genes of the anthocyanin pathway were recently identified at the bases of these novel genotypes [28]. One of them is *Aft*, an allelic variant of the gene *AN2like*, which may trigger the anthocyanin synthesis in fruit peel under high light [25], and was introgressed from *S. chilense*, one of the species of the “tomato clade” (corresponding to the Section Lycopersicon of the genus Solanum), which are frequently used as breeding material because are genetically close to *S. lycopersicum*, show colinear and homologous genomes [27], and result interfertile among them [29].

Wild Solanum species belonging to more distantly related lineages may offer new traits of potential interest to breeders. *S. lycopersicoides* is a very interesting wild relative of tomato, characterized by extreme resistance to many biotic and abiotic stresses and production of fruits which can naturally accumulate very high amounts of anthocyanins [27, 39]. This species belongs to the Section Lycopersicoides of the genus Solanum, which is more distantly related to the cultivated tomato than the species of the tomato clade [27]: its hybridization with *S. lycopersicum* is naturally limited by the existence of strong reproductive barriers and the common sterility of the hybrids [40]. However, in recent years *S. lycopersicoides* was artificially hybridized with tomato, obtaining introgression lines potentially very useful to increase its genetic variability [41–43].

### Is ***Abg*** allelic to ***Aft***? Finally, the answer

The *Abg* tomato accession introgressed from *S. lycopersicoides* the genetic locus on chromosome 10 linked with the synthesis of anthocyanins in the fruit epicarp under high light (Fig. 1). The possibility that *Abg* and *Aft* represent alleles of the same gene was early hypothesized [24, 25], but never proved so far. We started our analysis trying to understand how many and which of the *R2R3 MYB* genes lying in the long arm of chromosome 10 and known for their involvement in anthocyanin synthesis were inherited in *Abg* from the wild nightshade parental donor.

First, the presence of the entire *R2R3 MYB* cluster was confirmed in *Abg* plants (Fig. S1-S4). These genes showed significant genetic variability compared with tomato and resulted very similar to the sequences contained in the corresponding *S. lycopersicoides* genomic scaffolds [30] (Fig. S5-S8). This was a genetic confirmation of the origin of the *Abg* introgression from the wild nightshade and indicated that the entire *MYB* cluster derived from this species.

All these *Abg R2R3 MYB* genes showed the typical amino acidic signatures of positive anthocyanin MYB regulators: the [DE]Lx2[RK]x3Lx6Lx3R motif for interaction with bHLHs [44], and the ANDV sequence of Element 3 for regulation of anthocyanin synthesis [45] (Fig. S16b). A transactivation assay in protoplasts proved their ability to interact with the AN1 protein (the main bHLH partner of the R2R3 MYB TFs activating the anthocyanin synthesis in tomato [28, 46]) and to activate the promoter of *DFR*, a typical marker gene for anthocyanin synthesis (Fig. 4). For AN2, ANT1 and ANT1like no differences were observed between *Abg* and WT proteins in terms of activity (Fig. S18). As far as *AN2like* is concerned, the sequence in *Abg* was different from both WT and *Aft* but shared with the allele of *Aft* the SNP at the 5’ splice site of the second intron that was shown to be necessary for a correct splicing of the pre-mRNA to produce a functional TF [21] (Fig. S8). AN2like^Abg^ resulted indeed functional like AN2like^Aft^ in the transactivation assay of the promoter of *DFR* (Fig. 4a) and in the agroinfiltration assay in *N. benthamiana* leaves (Fig. 6a, b). We also found that *AN2like*^*Abg*^ was highly expressed in the fruit epicarp where anthocyanins were synthesized (Fig. 7), and, most importantly, its specific silencing via VIGS inhibited the anthocyanin pigmentation of the fruits (Fig. 8). All these results finally demonstrated that the “purple” phenotype of *Abg* and *Aft* fruits relies on the expression of functional alleles of the same gene, *AN2like*.

Very interestingly, we found the existence of an alternative splicing of the *AN2like*^*Abg*^ allele with the production of a shorter mRNA in addition to the canonically spliced functional transcript similar to the *Aft* one (Fig. S19). This short *AN2like*^*Abg*^ mRNA is translated in a protein which is functional but less efficient in the induction of anthocyanins than the longer one, both in vitro and in vivo assays (Figs. 5b and 6a and b). This is due to the lack of a 27-amino acidic sequence in a region of the C-terminus adjacent to the “S6B” motif (Fig. 5a), a sequence which is highly conserved in the R2R3 MYB TFs inducing anthocyanin synthesis and probably involved in protein-protein interactions [47]. Its artificial deletion in the AN2like^Aft^ TF produced a similar reduction in the capacity to induce anthocyanin synthesis (Figs. 5b and 6a and b), indicating that the sequence additionally spliced in the third exon of the gene did contain features important for enhancing the activity of the TF.

The long transcript appeared much more expressed than the short one in the mature green peel of the *Abg* fruits (Fig. S21). Alternative splicing strongly contributes to increase the diversity of the mRNAs expressed from a genome and the different isoforms produced from a single gene may show different biological functions [48]. It is therefore plausible that the alternative splicing of *AN2like*^*Abg*^ has a functional meaning and may contribute to the modulation of the production of anthocyanins in the fruit epicarp under environmental changes or at different times. This is the first evidence of the existence in tomato of mechanisms of alternative splicing possibly regulating the anthocyanin pigmentation of the fruits and further experiments are necessary to clarify this process.

### The ***Abg*** locus contains an additional ***R2R3 MYB*** gene

In *Abg* a novel *R2R3 MYB* gene, named *MYB113*^*Abg*^ for its similarity with other *Solanaceae* genes [49, 50], and encoding a protein carrying domains typical of MYB TFs involved in anthocyanin regulation, was identified (Fig. S9, S10). Its structural features, including the presence of the interaction domain with bHLHs and specific signatures conserved in activators of the anthocyanin pathway, are typical of the subgroup 6 of R2R3 MYBs [3]. MYB113^Abg^ resulted more similar to the TFs of the group of tomato AN2 and AN2like than to the group of ANT1 and ANT1like (Fig. S11). The presence of this gene was confirmed in *S. lycopersicoides* genome (Fig. S9): thus, also this fifth R2R3 MYB TF was introgressed in *Abg* from the wild parent. In a recent resequencing project of *S. lycopersicoides* genome, this gene was called *AN2-like2* for its similarity with *AN2like* [30].

In tomato a gene orthologous to *MYB113*^*Abg*^ is not known. However, a similar sequence was found in chromosome 10, close to *AN2like* and in a more distal position: it has not been annotated so far probably because its cds is very short: compared with *MYB113*^*Abg*^, this tomato genomic sequence showed indeed a large nucleotide deletion in the second intron and a SNP at the beginning of the third exon which created a premature stop codon (Fig. S12; Fig. S13). Actually, it was not able to transactivate the *DFR* promoter in vitro (Fig. 4b) or to activate anthocyanin synthesis (Fig. 6c, d). We thus concluded that the orthologous of MYB113^Abg^ was lost in tomato.

*MYB113*^*Abg*^ represents a novel gene involved in anthocyanin synthesis and its activity was demonstrated in the transactivation assay of the *DFR* promoter (Fig. 4b) and confirmed in vivo through agroinfiltration of *N. benthamiana* leaves (Fig. 6c, d). However, its expression was found negligible in *Abg* fruit peel (Fig. 7), indicating that it was not involved in fruit pigmentation. It resulted transcribed in vegetative tissues, but, excluding the stem in *Abg*/*Abg* plants, its expression level was very low (Fig. 7) and thus a functional role cannot be assigned at the moment. It would be interesting to understand if *MYB113* in *S. lycopersicoides* may be individually modulated according to different environmental or developmental factors. In eggplant, a TF with a sequence similar to MYB113^Abg^ can increase anthocyanin synthesis under low temperatures through interaction with C-repeat binding factors, which act as central regulators in cold response [50]. Due to the natural habitat of *S. lycopersicoides* plants, characterized by high light intensities and low temperatures [27], a similar function for MYB113^Abg^ might be hypothesized.

Summarising, the R2R3 MYB cluster in chromosome 10 contains five functional genes in *Abg*: this is distinctive from both WT tomato, where only three genes out of five can produce functional TFs (*AN2*, *ANT1* and *ANT1like*) (Fig. S18), and *Aft* plants, where *AN2like* is correctly spliced as in *Abg*, but *MYB113* is mutated and not functional as in tomato (Fig. S23c). Remarkably, significant rearrangements in *MYB113* were identified in all the other species of the tomato clade whose genomic sequences were available (Fig. S23). *MYB113* sequence resulted complete only in *S. lycopersicoides* and *S. sitiens*, both belonging to the Section Lycopersicoides [27]: this means that the loss of MYB113 activity probably occurred in the separation between the Sections Lycopersicoides and Lycopersicon of the genus Solanum, whereas the activity of AN2like, being it functional in both *Abg* and *Aft*, was maintained in the tomato clade, at least till the separation of the green-fruited species from the red ones [28].

### In ***Abg***/***Abg*** plants a positive regulator of the anthocyanin pathway is absent in vegetative tissues

As far as we know, the *Abg* accession originated from a spontaneous cross between *S. lycopersicum* and *S. lycopersicoides*, but the original tomato background is not known. The line has been maintained in collection as heterozygous plants, being the homozygous unstable and not fertile [25]. We found, as expected, that *Abg*/*Abg* plants were sterile, and produced small malformed and seedless fruits (Fig. 2d), whereas the heterozygotes were fertile. The genetic aspects were not the focus of our work, but homozygous and heterozygous plants captured our attention for their peculiar phenotype in terms of anthocyanin pigmentation in vegetative tissues. Leaves, leaf veins and stems in young *Abg*/*Abg* plants were pale green due to a strong deficiency of anthocyanins and this phenotype tended to persist, even if leaves could increase their anthocyanin content over time (Fig. 2). Such a phenotype may be consequence of two different scenarios: the expression of a strong repressor of the anthocyanin biosynthetic pathway in the *Abg*/*Abg* plants, probably deriving from the wild parental donor being more active when in homozygosis, or the presence of an activator of the pathway in the *Abg*/+ plants, deriving from the tomato parental donor and absent in the homozygotes.

We looked for a putative negative regulator of the pathway among the genes located in the long arm of chromosome 10 and focused our attention on the protein encoded by the *THM27* gene [51, 52], which shows the typical structural features of the R2R3 MYB repressors, included an EAR motif in the C-terminal domain (Fig. S14b), characteristics which are typical of subgroup 4 of R2R3 MYBs [3]. This gene in *Abg* was indeed introgressed from *S. lycopersicoides* (Fig. S15) and its protein could effectively repress the activation of the promoter of *DFR* in a transactivation assay, confirming its putative role of repressor (Fig. 4c). However, its activity did not result significantly different from that of the THM27^WT^ protein (Fig. 4c), and the expression level of the gene in vegetative tissues was similar in homozygous and heterozygous plants (Fig. 7). Furthermore, the *THM27*^*Abg*^ allele was expressed at levels similar to the *THM27*^*WT*^ allele in the heterozygotes (Fig. S20c). These results, even if very interesting because confirming for the first time the role of THM27 as a negative regulator of the anthocyanin pathway in tomato, did not allow us to correlate its activity with the peculiar vegetative phenotype shown by *Abg* homozygous plants.

In alternative, the lack of a WT dominant activator of the pathway might be responsible of the “anthocyanin-free” phenotype. Such an activator should be expressed in the heterozygous *Abg* plants because they maintain single copies of the WT alleles in the genomic region corresponding to the segment introgressed in chromosome 10. AN2 would be the favourite candidate, being the master anthocyanin positive regulator in tomato vegetative tissues [15]. Remarkably, whereas the capacity of the *Abg* allele of *AN2* to transactivate the promoter of *DFR* was identical to the capacity of the WT allele (Fig. S18a), their expression levels in the vegetative tissues of the heterozygous plants (where both the alleles are transcribed) appeared very different, with *AN2*^*WT*^ showing a good expression level and *AN2*^*Abg*^ being very little expressed (Fig. 7; Fig. S20a). Very low levels of *AN2*^*Abg*^ transcripts were measured also in the homozygous plants, where the WT allele is not present (Fig. 7). Thus, the lack of a significant expression of the gene *AN2* in the vegetative tissues might be at the basis of the scarce capacity of the homozygous *Abg* plants to synthesise anthocyanins in those organs. Differences in the promoter regions of the WT and *Abg* alleles in terms of cis-acting elements might be responsible for such a significant diversity in their response to the same environmental and developmental cues. Other studies are necessary to understand if in *S. lycopersicoides* R2R3 MYB TFs different from *AN2* and encoded by genes located outside of the genomic region introgressed in *Abg*, substitute it in its role of main activator of the anthocyanin pathway in vegetative tissues, or if AN2 is active in other developmental stages or environmental conditions.

## Conclusion

This study allowed us to genetically and functionally demonstrate that the anthocyanin-rich fruit phenotype of the *Abg* accession was due to the expression in the fruit epicarp of a functional allele of *AN2like*, which can be transcribed in a pre-mRNA correctly spliced in a TF taking part to the MBW complex inducing the anthocyanin biosynthetic pathway in fruits. This clearly resembles what already found in the *Aft* accession [16, 21]. In *Abg* plants, however, a further modulation of the pathway has been observed, since the *AN2like*^*Abg*^ allele can be alternatively spliced in two transcripts characterized by different length and activity, and this is the first report of a mechanism of alternative splicing in a R2R3 MYB TF gene which may fine tune the synthesis and the accumulation of these pigments in the fruit epicarp. Furthermore, the strong deficiency of anthocyanins observed in the vegetative tissues of *Abg* homozygous plants was due to failed expression of all the *R2R3 MYB* genes encoding positive activators of the pathway, and in particular of the gene *AN2*, which is the most important one in tomato [15]. An additional TF, MYB113^Abg^, was finally identified in *Abg* as produced from a novel gene belonging to the *R2R3 MYB* cluster, potentially able to act as a positive regulator of the anthocyanin pathway and lost in tomato and in its closest relatives. This gene might work in specific developmental stages or environmental conditions, giving to *Abg* plants a trait which is not present anymore in tomato, and which is, for this reason, worthy of further studies.

## Methods

### Plant materials and growth conditions

Seeds of *S. lycopersicum* cv. Ailsa Craig (LA2838A) and *Abg* (LA3668) were provided by the Tomato Genetic Resource Center (https://tgrc.ucdavis.edu/). Seeds were germinated in rock-wool plugs (Grodan) soaked in a nutritive solution [53]. Two-week-old seedlings were transplanted in pots containing a 70:30 soil/expanded clay mixture and placed in a growth chamber with 23 °C/20°C day/night temperature, 12 h photoperiod, 150 µmol photons m^− 2^ s^− 1^, and 40% relative humidity.

### Gene cloning and plasmid construction

Genes of *Abg* plants orthologous to tomato *Solyc10g086250* (*AN2*), *Solyc10g086260* (*ANT1*), *Solyc10g086270* (*ANT1like*), *Solyc10g086290* (*AN2like*), and *Solyc10g055410* (*THM27*) genes (SOL Genomics Network, https://sgn.cornell.edu), as well as the sequence of *MYB113*^*Abg*^ gene, were amplified by PCR starting from *Abg* +/+ genomic DNA using the “Phusion High-Fidelity DNA Polymerase” (Thermo Fisher Scientific) and the oligonucleotide primers reported in Table S1. Full length cds of *Abg AN2like* and *MYB113* were amplified from RNA extracted from leaves or fruit peel using the “Spectrum Plant Total RNA Kit” (Merck), treated with DNase and reverse-transcribed with the SuperScript IV Reverse Transcriptase (Thermo Fisher Scientific) using the same primers reported in Table S1. The sequences were individually cloned into pENTR/D-TOPO vector (Thermo Fisher Scientific) and sequenced (Eurofins Genomics). For mutagenesis of *AN2like*^*Aft*^ transcript, the entry vector containing the sequence, prepared in a previous study [16], was amplified by overlap extension PCR using the pair of primers listed in Table S2, and the shorter *AN2like*^*Aft*^ cds was cloned as described above. The entry clones were recombined with different destination vectors, as described below, via Invitrogen™ Gateway™ recombination cloning technology (Thermo Fisher Scientific). Multiple sequence alignments were performed using ClustalW (https://www.ebi.ac.uk/Tools/msa/clustalo) sequence analysis software.

### Cleaved amplified polymorphic sequences analysis

Amplified DNA fragments with specific oligonucleotide primers (Table S1), producing a 444 nt product from WT and a 439 nt product from *Abg*, were digested with the restriction endonuclease Spe I, whose site is present only in the *AN2like*^*WT*^ sequence, to display by gel electrophoresis different patterns according to genotypes.

### Phylogenetic analysis

The analysis was performed on the Phylogeny.fr platform [54] using default programs and parameters. MUSCLE was used for multiple alignment and PhyML for phylogeny.

### Anthocyanin quantification

Anthocyanins were extracted and quantified as described in [31], and finally expressed as mg petunidin-3-(p-coumaroyl rutinoside)-5-glucoside g^− 1^ fresh weight [15].

### Transient transformation of tomato protoplasts

Leaf protoplasts were isolated following the protocol in [55] from 3-week-old Micro-Tom plants, grown as reported above. Polyethylene glycol-mediated protoplast transformation was carried out as in [56].

### Transactivation assays in tomato protoplasts

Transactivation assays by dual-luciferase system were performed with the *Renilla reniformis* (Renilla) and *Photinus pyralis* (Firefly) luciferase (Luc) enzymes. The effector constructs *35S:AN2*, *35S:ANT1*, *35S:ANT1like*, *35S:AN2like*, *35S:MYB113*, and *35S:THM27* were produced as reported in [31] with the *R2R3 MYB* sequences cloned from *Abg* and Ailsa Craig DNA. The effector constructs *35S:AN2like_*long transcript and *35S:AN2like_*short transcript were produced with the two transcripts of the *AN2like* allele isolated from *Abg* fruit peel. All the other effector plasmids containing Ailsa Craig or *Aft MYB* sequences, the effector construct *35S:AN1*, and the *promoter_DFR:Firefly_Luc* reporter construct were produced in a previous study [31]. A *35S:Renilla_Luc* vector was used to normalize luminescence values detected in protoplasts [57]. Effector and reporter plasmids were co-transfected in protoplasts (5 µg for each effector plasmid, 5 µg for the *promoter_DFR:Firefly_Luc *reporter construct and 2.5 µg for the *35S:Renilla_Luc* vector) and luminescence relative levels were measured as described in [15]. In each assay, data were expressed as relative luciferase activity (Firefly_Luc/Renilla_Luc).

### Agro-infiltration assay

Transient expression assay was performed using *Nicotiana benthamiana* plants cultivated in a growth chamber with 16 h daylight, 100 µmol photons m^− 2^ s^− 1^, 23 °C/20°C day/night temperature. Overexpression vectors were generated by recombining the entry clones containing the sequences of *AN2like*, *MYB113*, and *AN1* with the Gateway™ compatible binary vector pK7WG2 [58]. *Agrobacterium tumefaciens* GV3101 (MP90) strains hosting the different constructs were infiltrated in Nicotiana leaves following the protocol of [59]. Each leaf was infiltrated in four or five points with different constructs. pK7WG2 vectors recombined with the sequence of *AN1* were singularly used as negative controls. Anthocyanins were quantified in single portions sampled from leaves in relation to the infiltrated areas at five days after infiltration.

### RNA isolation, cDNA synthesis, and quantitative RT-PCR analysis (qPCR)

Total RNA, extracted from leaves, leaf veins, stems and fruit peel with the “Spectrum™ Plant Total RNA Kit” (Merck) was subjected to DNase treatment and then reverse transcribed into cDNA using the “Maxima First Strand cDNA Synthesis Kit for RT-qPCR, with dsDNase” (Thermo Fisher Scientific). Quantitative RT-PCR was performed with a QuantStudio 3 Real-Time PCR system (Applied Biosystems) using the “PowerUp™ SYBR® Green Master Mix” (Thermo Fisher Scientific) and the primers listed in Table S3 and in [15]. Expression levels relative to the reference gene *Elongation Factor 1-alpha* (*EF1A*) were quantified for each target gene.

### Cellular localization of MYB proteins

The *Abg AN2like* and *MYB113* entry vectors were recombined with the Gateway™ compatible destination vector p2FGW7 [58]. Protoplasts were isolated as described, transformed with 5 µg DNA for each plasmid, and incubated in the dark at 25 °C for 16 h before subsequent analysis. Fluorescence for GFP and RFP was imaged with a Nikon Eclipse Ti-5 video-confocal microscope (https://www.nikon.it) using suitable filters.

### Virus Induced Gene silencing (VIGS)

TRV-based T-DNA binary vectors pTRV1, pTRV2 and pTRV2/GATEWAY are from [60]. A fragment of the cDNA of *AN2like*^*Abg*^ was amplified using the oligonucleotide primers listed in Table S1. The amplified sequence was cloned into pENTR/D-TOPO vector (Thermo Fisher Scientific) and sequenced (Eurofins Genomics). The entry clone was then recombined into the Gateway compatible pTRV2 vector. Agrobacterium cultures were grown as described in [61], and cell concentration in the infiltration media was adjusted to an OD of 0.05. *Abg* fruit peel, from fruits bagged with a light-impermeable cover for two weeks starting from the immature green stage, was agroinfiltrated with a 1:1 (v/v) mixture of two *A. tumefaciens* GV3101 strains, containing the pTRV1 and the pTRV2 binary vectors with the silencing fragment (or the empty pTRV2 as control), respectively. Infiltrated fruits were kept in the dark for other 4 days and then grown under light to promote anthocyanins accumulation. Fruits were photographed and peel sampled at the mature green stage. The RNA was extracted from silenced and unsilenced fruits and the expression levels of regulatory and target genes were measured as described above.

### Statistics

Statistical analyses were performed with GraphPad Prism 9.00 (www.graphpad.com/scientific-software/prism/). Data were analysed by t-test or one-way ANOVA with differences measured using the Tukey’s honest significant difference (HSD) multiple comparisons test.

## Electronic supplementary material

Below is the link to the electronic supplementary material.

**Additional File 1: Fig. S1**. Sequence of the gene *AN2* in *Abg*, *S. lycopersicoides* and WT plants. **Fig. S2.** Sequence of the gene *ANT1* in *Abg*, *S. lycopersicoides* and WT plants. **Fig. S3.** Sequence of the gene *ANT1like* in *Abg*, *S. lycopersicoides* and WT plants. **Fig. S4.** Sequence of the gene *AN2like* in *Abg*, *S. lycopersicoides*, WT and *Aft* plants. **Fig. S5.** ClustalW alignment of the gene *AN2* sequenced in *Abg*, *S. lycopersicoides* and WT plants. **Fig. S6.** ClustalW alignment of the gene *ANT1* sequenced in *Abg*, *S. lycopersicoides* and WT plants. **Fig. S7.** ClustalW alignment of the gene *ANT1like* sequenced in *Abg*, *S. lycopersicoides* and WT plants. **Fig. S8.** ClustalW alignment of the gene *AN2like* sequenced in *Abg*, *S. lycopersicoides*, WT and *Aft* plants. **Fig. S9.** Sequence analysis of the gene *MYB113* in *Abg* and *S. lycopersicoides*. **Fig. S10.** MUSCLE 3.8.31 alignment of the sequences of *Solanaceae* R2R3 MYB proteins similar to MYB113 identified in *Abg*. **Fig. S11.** Phylogenetic tree of MYB113^*Abg*^ and other *Solanaceae* R2R3 MYB proteins. **Fig. S12.** Analysis of the gene *MYB113* identified in *Abg* in WT plants. **Fig. S13.** ClustalW alignment of the gene *MYB113* sequenced in *Abg*, *S. lycopersicoides* and WT plants. **Fig. S14.** Sequence analysis of the gene and protein THM27 in *Abg*, *S. lycopersicoides* and WT plants. **Fig. S15.** ClustalW alignment of the gene *THM27* sequenced in *Abg*, *S. lycopersicoides* and WT plants. **Fig. S16.** cds and protein sequences of the R2R3 MYB transcription factors belonging to the cluster of chromosome 10 cloned in *Abg* plants. **Fig. S17.** CAPS marker for the gene *AN2like*. **Fig. S18.** Dual-Luc assay of the genes *AN2*, *ANT1* and *ANT1like*. **Fig. S19.** *AN2like* transcripts and relative proteins in *Abg* plants. **Fig. S20.** Expression levels of the WT and *Abg* alleles of the regulatory *R2R3 MYB AN2*, *AN2like*, and *THM27* genes in *Abg* heterozygous plants. **Fig. S21.** Expression levels of the total transcripts (long transcript + short transcript) and the short transcripts of the *AN2like*^*Abg*^ gene analysed by qPCR in the fruit peel of *Abg* homozygous and heterozygous plants. **Fig. S22.** qPCR analysis of the regulatory *R2R3 MYB* gene *AN2*, *ANT1* and *ANT1like* in the peel of *Abg*/*Abg* unsilenced or silenced fruits. **Fig. S23.** Sequence analysis of the gene *MYB113* from different tomato accessions and wild Solanum species.

**Additional File 2: Table S1.** List of oligonucleotide primers used for gene cloning, CAPS analysis and as VIGS guides. **Table S2.** List of oligonucleotide primers used for mutagenesis of *AN2like*^*Aft*^. **Table S3.** List of oligonucleotide primers used for qPCR analysis.

## Data Availability

The data used to support the findings of this study are available from the corresponding author upon request. The sequences of the *Abg R2R3 MYB* genes and transcripts are now being deposited in GenBank (https://www.ncbi.nlm.nih.gov/genbank): *ANT1like*^*Abg*^ gene (OP094090); *ANT1*^*Abg*^ gene (OP094091); *AN2like*^*Abg*^ gene (OP094092); *AN2*^*Abg*^ gene (OP094093); *MYB113*^*Abg*^ gene (OP094094); *THM27*^*Abg*^ gene (OP094098); *ANT1*^*Abg*^ mRNA (OP094102); *AN2like*^*Abg*^ long form mRNA (OP094099); *AN2like*^*Abg*^ short form mRNA (OP094100); *MYB113*^*Abg*^ mRNA (OP094101). The following sequences were also deposited: *MYB113*^*Aft*^ gene (OP094096); *Solanum chilense MYB113* gene (OP094097); *MYB113*^*WT*^ gene (OP094095).

## List of abbreviations

*Aft*: *Anthocyanin fruit*
*Abg*: *Aubergine*
TF: transcription factor
MYB: MYeloBlastosis
MBW: MYB-bHLH-WDR
bHLH: basic Helix-Loop-Helix
WDR: WD40-repeat
SNP: single nucleotide polymorphism
nt: nucleotide
VIGS: Virus Induced Gene Silencing
WT: wild type
