# Supplementary material for: Novel R2R3 MYB transcription factors regulate anthocyanin synthesis in Aubergine tomato plants
Source: BMC Plant Biol. 2023 Mar 20;23:148. doi: 10.1186/s12870-023-04153-7 (PMC10026432; doi:10.1186/s12870-023-04153-7)
Supplement: Supplementary file 1 — Additional File 1: Fig. S1. Sequence of the gene AN2 in Abg, S. lycopersicoides and WT plants. Fig. S2. Sequence of the gene ANT1 in Abg, S. lycopersicoides and WT plants. Fig. S3. Sequence of the gene ANT1like in Abg, S. lycopersicoides and WT plants. Fig. S4. Sequence of the gene AN2like in Abg, S. lycopersicoides, WT and Aft plants. Fig. S5. ClustalW alignment of the gene AN2 sequenced in Abg, S. lycopersicoides and WT plants. Fig. S6. ClustalW alignment of the gene ANT1 sequenced in Abg, S. lycopersicoides and WT plants. Fig. S7. ClustalW alignment of the gene ANT1like sequenced in Abg, S. lycopersicoides and WT plants. Fig. S8. ClustalW alignment of the gene AN2like sequenced in Abg, S. lycopersicoides, WT and Aft plants. Fig. S9. Sequence analysis of the gene MYB113 in Abg and S. lycopersicoides. Fig. S10. MUSCLE 3.8.31 alignment of the sequences of Solanaceae R2R3 MYB proteins similar to MYB113 identified in Abg. Fig. S11. Phylogenetic tree of MYB113Abg and other Solanaceae R2R3 MYB proteins. Fig. S12. Analysis of the gene MYB113 identified in Abg in WT plants. Fig. S13. ClustalW alignment of the gene MYB113 sequenced in Abg, S. lycopersicoides and WT plants. Fig. S14. Sequence analysis of the gene and protein THM27 in Abg, S. lycopersicoides and WT plants. Fig. S15. ClustalW alignment of the gene THM27 sequenced in Abg, S. lycopersicoides and WT plants. Fig. S16. cds and protein sequences of the R2R3 MYB transcription factors belonging to the cluster of chromosome 10 cloned in Abg plants. Fig. S17. CAPS marker for the gene AN2like. Fig. S18. Dual-Luc assay of the genes AN2, ANT1 and ANT1like. Fig. S19. AN2like transcripts and relative proteins in Abg plants. Fig. S20. Expression levels of the WT and Abg alleles of the regulatory R2R3 MYB AN2, AN2like, and THM27 genes in Abg heterozygous plants. Fig. S21. Expression levels of the total transcripts (long transcript + short transcript) and the short transcripts of the AN2likeAbg gene analysed by qPCR in the fru [file 12870_2023_4153_MOESM1_ESM.pdf]

Additional File 1

Novel R2R3 MYB transcription factors regulate anthocyanin synthesis in *Aubergine* tomato plants

Authors: Jacopo Menconi, Pierdomenico Perata and Silvia Gonzali*

Affiliations: PlantLab, Center of Plant Sciences, Scuola Superiore Sant'Anna, Piazza Martiri della Libertà 33, 56127 Pisa, Italy

*Author for correspondence: s.gonzali@santannapisa.it (Silvia Gonzali)

This file includes: Figure S1 to S23

>*AN2*^{Abg}

ATGAATACTCCTATGTGTGCATCGTTGGGAGTTAGGAAAGGTTCATGGACTGAACAAGAAGATTTTCTTTTAAGAAATTGCATTCAAAAATATGGTGAAG
GAAAGTGGCATCTTGTTCCTGCTAGAGCTGGTATTACATTTATTTATTTTTAGTTCGTTTAAAAAAAATATATTTTTATATTTAAAATAATTTATTAAAC
TTTAAATTTTGATTTGACTTCTAATAACATAATTAATAGTTTTATGATTTATTTTAAATCATAAGTTTTATACTCCCTTAATTTAATGTGAATATGTGTA
GGTTTGAATCGATGTCGAAAGAGTTGCAGACTAAGGTGGCTAAATTATCTAAGGCCACATATCAAGAGAGGTGACTTTGCTCCAGATGAAGTAGATCTCA
TCTTGAGACTTCATAAACTCTTAGGCAATAGGTAAGTCTATGTATTTAGTGGATTGCAATCATTCCATTCAAAAGCTTTTAATATTACAAAGTTATGATA
TACTCCGTCTGTTTTTTAAAAAAAGATCTCATTTCTTTTTCAGTCTGTTTAAAAAAGAATAAAAAAATTTTTTGACAAAACTTTAATTTTAACTTTTCAC
GTGACATGTTTAAGACCACAAGATTAAAAGACATTTTAATACATTTGACATAACTTTAATTTAGAACCACAAGATAAAAAAATCTTATTTCTTTTCTTAA
ACTTCGTTCCAAGTCAAACTAAACCATTTTTTTTAAAACAGAGGGAGTAATTTTCTTTATATTATATTATATATATATATGTATAACCATATCTTAATAT
GTGTAATTATTTCATTTAAAAGTTACGCTATTTTGTGTAGGTGGTCACTTATTGCTGGTAGACTTCCAGGAAGGACTGCAAATGATGTTAAGAACTATTG
GAACACTCACTTTCATAAGAAGTTAAATATTATTGCTCCTCATCTTCATCCTCGTCCTCGTCCTCATCCTCATCTACAGATTAAGCATAAGAGCATCGCG
GTTACTAAGAATGAAATAATAAAACCTCAACCTCGGAACTTCTCAAACGTTAATAAGAATATTTCTCATTGGTGCAACAACAACAAAAGTATGATCACAA
ACACATTAGACAAAGATGACAAACGTTGCAAGGAGATGGTAGTAAATATTTGTGAGAAGCCAATAGGAGAAAATACATCGTCGATAGACGATGGAGTTGA
ATGGTGGACAAATTTACTGGTAAATTGCAATGAAATTGAAGAAGAAGCTGTTGTTACAAATTTTGAAAAGACACCAACAATGTTGTTAAATGAGGAAATA
TCACCACCGTTAATAATTAATGGTGAAGGCAACTCCATGCAACAAGGACAAACTCATGATAATTGGGATGACTTTTCAACTGATATTGACTTATGGAATC
TACTTAATTAA

>*AN2*^{Sly}

ATGAATACTCCTATGTGTGCATCGTTGGGAGTTAGGAAAGGTTCATGGACTGAACAAGAAGATTTTCTTTTAAGAAATTGCATTCAAAAATATGGTGAAG
GGAAGTGGCATCTTGTTCCTGCTAGAGCTGGTATTACATTTTTTTCTTTTTTAGTTCATTTTAAAAAAAATATTTTTACATATAAAAATAATTTATTAAA
CTTTAAATTTTGATTTTACTTTTAATAACATGATTTATAGTTTTATGATTTGTTTTAAATCATAAGTTTTATAATATTTTAATTTAATGTGAATATGTGT
AGGTTTAAATCGATGTCGAAAAAGTTGCAGACTAAGGTGGCTAAATTATCTAAGGCCACATATCAAGAGAGGTGACTTTGCTCCGGATGAAGTTGATCTC
ATCTTGAGACTTCATAAACTCTTAGGCAATAGGTAAGTCTATGTATTCAGTGGATTGCAATCATTTCATTCAAAAGCTTTTAATATTAAAAAAATATGAT
ATATTCTCTTCGTTTTAAAAAAAATAATTTCATTTTCTTTTTAATCTGTTTAAAAAAGAATGACATTTTTTCTTTTTTGGCAAAACTTTAATTTTAACTT
TTCACGTGGCATATTTAAGACCACAAGATTAAAAGATATTTTAATACATTTGACATAACTTTAATTAAGAATCACAAGATAAAAAAATTCTTATTTCTTT
TCTTAAACCCCGTTCCAACTCAAACTAGACCATTCTTTTTGAAACGGAGAGAGTAATTTTCTTTATATTATATTTTATATATATATATGTATAACCATAT
CTTAATACGTGTAATTATTTCATTTAAAAGTTACGCTATTTTGTGTAGGTGGTCACTTATTGCTGGTAGACTTCCAGGAAGGACTGCAAATGATGTGAAG
AACTATTGGAACACTCACTTTCATAAGAAGTTAAGTATTATTGCTCCTCATCTTCATCCTCGTCCTCGTCCTCGTCCTCATCCTCATCTACAGATTAATC
ATAAGAGCATCGCGGTTACTAAGAATGAAATAATAAAACCTCAACCTCGGAACTTCTCAAACATTAATAAGAATATTTCTCATTGGTGCAACAACAACAA
AAGTATGATCACAAACACATTAGACAAAGATGACAAACGTTGCAAGGAGATGGTAGTAAATATTAGTGAGAAGCCAATAGGAGAAAATACATCGTCGATA
GACGATGGAGTTGAATGGTGGACAAATTTACTGGAAAATTGCAATGAAATTGAAGAAGAAGCAGTTGTTACAAATTTTGAAAAAACACCAACAATGTTGT
TACATGAGGAAATATCACCACCGTTAATAATTAATGGTGAAGGCAACTCCATGCAACAAGGACAAACTCATGATAATTGGGATGACTTTTCAACTGATAT
TGACTTATGGAATCTACTTAATTAA

>*AN2*^{WT}

ATGAATACTCCTATGTGTGCATCGTTGGGAGTTAGGAAAGGTTCATGGACTGAACAAGAAGATTCTCTTTTAAGAGATTGCATTCAAAAATATGGTGAAG
GAAAGTGGCATCTTGTTCCTGCTAGAGCTGGTATTACTTTTGTACTTTTTCTAATTTGTTTTAAAAAATAATGTATTTTATATATTTATAAATGATTTAC
TAAATTCTAAGTTTTGATTTTACTTTCTAATAACATAATTTATAGTTTTATGATTTGTTTTAAATCATAAATTTTTGTAATATTTTATATTTATTTATTA
ATTTTGAATGTTATGTGAACATGTATAGGTTTAAATCGATGTCGAAAGAGTTGCAGACTAAGGTGGCTAAACTATCTAAGGCCACATATCAAGAGAGGTG
ACTTTGCTCCAGATGAAGTGGATCTCATCTTGAGACTTCACAAACTCTTAGGCAATAGGTAAGTCTATGCTGCAATTATTTCATTCAAAAGTTTTTGATG
TTATATTTTTTTTTATATTGTATTGGTCAAATATATAAGTAGATTTTTAAATTTGTTGGATTTTCTTCCTCAGATATTTCAATTACGTCATTTTTCTGTA
TAATCATTGAACTCCCATAATTTGTTCCATTTAAACAATCATCGTTGTCTGATGTGGATCAAGATTTGTGAGGGTATTGTTTCACAAATCATAGTCCAAT
TGATAGTGAAAATGTTTGAGAATAATTGTGTTTTACTGAAGTCATTTTGAACACATTAGAAAACAAATGAGACTAGCACGGTTTGTCTTCATTCCTTCTA
TCAAAATTATTACAACTCAAACACAATCGAAGACATTTACAATAGAAAAGCTGATCAAAAGTGTTTTAAAAAAACAAATTATGGGTGGTTCAATGATTCA
ATTGAAAAATGACGTAATTGAGGTACCTGAGAAAAAAAAAACCTAAACATATGTGTAATTATTTCATTTAAAAGTTAGTTATTTTGTATAGGTGGTCACTT
ATTGCTGGTAGACTTCCAGGAAGGACAGCAAACGATGTGAAGAACTATTGGAATACACACTTTCACAAGAAGTTAAGTATTATTGCTCCTCATCTACATC
CTCATTCTCGTCCTCGTTCTCATCCTCGTCTACAGATTAAGCATAAGAGCATCGCGGTTACTAAGAATGAAATAATAAGACCTCAACCTCGGAACTTCTC
AAACGTTAAGAAGAATGATTCTCATTGGTGCAACAACAAAAGTATGATCACAAACACATTAGACAAAGATGACAAACGTTGCAACGAAATCGTTGTAAAT
ATTTGTGAGAAGCCAATAGGAGAAAATACATCGTCGATAGACGATGGAGTTGAATGGTGGACAAATTTACTGGAAAATTGCATTGAAATTGAAGAAGAAA
CAGCTAATACAAATTTTGGAAAAACACCAACAATGTTGTTACATGAGGAAATATCACCACCGTTAGTTAATGGTGAAGACAACTCCATGCAACAAGGACC
AACTAATAATTGGGATGACTTTTCAACTGATATTGACTTATGGAATCTACTTAATTAA

Fig. S1. Sequence of the gene *AN2* in *Aubergine* (Abg), *S. lycopersicoides* (Sly) and wild type (WT) plants. Exons are highlighted in grey.

>*ANT1*^{Abg}

ATGAACAGTACATCTATGTCTTCATTGGGAGTGAGAAAAGGTTCTTGGACTGATGAAGAAGATTTTCTTTTAAGAAAATGTATTAACAAGTATGGTGAAG
GAAAATGGCATCTTGTTCCCATGAGAGCTGGTAATTAAATTAACTATCGCATTATTTTATCTGTCTGTCTCATTTTATATGATGTTATTTAAAAAATTAT
GTGAAAATGTATGTGCAGGTCTGAATAGATGTCGGAAAAGTTGTAGATTGAGGTGGCTGAATTATCTAAGGCCACATATCAAAAGAGGTGACTTTGAACA
AGATGAAGTGGATCTCATTTTGAGGCTTCATAAGCTCTTAGGCAACAGGCATGCAATTTTATGTTTTGACAAATTTGATTAATATAATATATATATTTGT
GTGACTATTTCATCTAAACGTTACGTTATTTTATGCAGATGGTCACTTATTGCAGGTAGACTTCCAGGAAGGACAGCTAACGACATAAAAAACTATTGGA
ACACTAACCTTCTAAGGAAGTTAAATACTAGTAAAATTGTTCCTCGTGAAAAGATTAACAATAAGTGTGGAGAAATTAGTACTAAGATTGAAATAATAAA
ACCTCAACCTAGGAAGTATTTCTCAAGCACAAAGATGAATATTGTAATTTTTGATGAGGAGGAACATTGCAAGGAAATAATAAGTGAGAAGCAAACTCCA
GATGCATCGATGGACAACGTAGATCAATGGTGGACAAATTTACTGGAAAATTGCAATGACGATGTTGAAGAAGATGAAGAGGTTGTAATTAATTATGAAA
AAACACTAACAAGTTTGTTACATGAAGAAATATCACCACCATTAAATGGTGAAGGTAACTCTATAATGCAACAAGGACAAACAAGTCATGATAGTTGGGG
TGACTTTTCTCTTAATTTACCACCCATGCAACAAGGAGTACAAAATGATGATTGGGATGATTTTTCTGCTGAAATTGACTTATGGAATCTACTTGATTAA

>*ANT1*^{Sly}

ATGAACAGTACATCTATGTCTTCATTGGGAGTGAGAAAAGGTTCTTGGACTGATGAAGAAGATTTTCTTTTAAGAAAATGTATTAACAAGTATGGTGAAG
GAAAATGGCATCTTGTTCCCATGAGAGCTGGTAATTAAATTAACTATCGCATTATTTTATCTGTCTGTCTCATTTTATATGATGTTATTTAAAAAATTAT
GTGAAAATGTATGTGCAGGTCTGAATAGATGTCGGAAAAGTCGTAGATTGAGGTGGCTGAATTATCTAAGGCCACATATCAAGAGAGGTGACTTTGAACA
AGATGAAGTGGATCTCATTTTGAGGCTTCATAAGCTCTTAGGCAACAGGCATGCAATTTTATGTTTTGACAAATTTGATTAATATAATATATATATATGT
GTGACTATTTCATCTAAACTTTACGTTATGTTATGCAGATGGTCACTTATTGCAGGTAGACTTCCAGGAAGGACAGCTAACGACATAAAAAACTATTGGA
ACACTAACCTTCTAAGGAAGTTAAATACTAGTAAAATTGTTCCTCGTGAAAAGATTAACAATAAGTGTGGAGAAATTAGTACTAAGATTGAAATAATAAA
ACCTCAACCTAGGAAGTATTTCTCAAGCACAAAGACGAATATTGTAATTTTGGATGAGGAGGAACATTGCAAGGAAATAATAAGTGAGAAGCAAACTCCA
GATGCATCGATGGACAACGTAGATCAATGGTGGACAAATTTAGTGGAAAATTGCAATGACGATGTTGAAGAAGATGAAGAGGTTGTAATTAATTATGAAA
AAACACTAACAAGTTTGTTACATGAAGAAATATCACCACCATTAAATGGTGAAGGTAACTCTATGCAACAAGGACAAACAAGTCATGATAGTTGGGGTGA
CTTTTCTCTTAATTTACCACCCATGCAACAAGGAGTACAAAATGATGATTGGGATGATTTTTCTGCTGAAATTGACTTATGGAATCTACTTGATTAA

>*ANT1*^{WT}

ATGAACAGTACATCTATGTCTTCATTGGGAGTGAGAAAAGGTTCATGGACTGATGAAGAAGATTTTCTTCTAAGAAAATGTATTGATAAGTATGGTGAAG
GAAAATGGCATCTTGTTCCCATAAGAGCTGGTAACCTATTAAATTAACTATCACGTTATTTTTATTTGTCTTTCTGTCTCATTTTATTTGACGTTATTAC
GAATATCATCTGAAAATGTACGTGCAGGTCTGAATAGATGTCGGAAAAGTTGTAGATTGAGGTGGCTGAATTATCTAAGGCCACATATCAAGAGAGGTGA
CTTTGAACAAGATGAAGTGGATCTCATTTTGAGGCTTCATAAGCTCTTAGGCAACAGGCATGCAAGTTTATGTTTTGACAAAATTTGATTAGTATATATT
ATATATACGTGTGACTATTTCATCTAAATGTTACGTTATTTTACGTAGATGGTCACTTATTGCTGGTAGACTTCCCGGAAGGACAGCTAACGATGTGAAA
AACTATTGGAACACTAATCTTCTAAGGAAGTTAAATACTACTAAAATTGTTCCTCGCGAAAAGATTAACAATAAGTGTGGAGAAATTAGTACTAAGATTG
AAATTATAAAACCTCAACGACGCAAGTATTTCTCAAGCACAATGAAGAATGTTACAAACAATAATGTAATTTTGGACGAGGAGGAACATTGCAAGGAAAT
AATAAGTGAGAAACAAACTCCAGATGCATCGATGGACAACGTAGATCCATGGTGGATAAATTTACTGGAAAATTGCAATGACGATATTGAAGAAGATGAA
GAGGTTGTAATTAATTATGAAAAAACACTAACAAGTTTGTTACATGAAGAAATATCACCACCATTAAATATTGGTGAAGGTAACTCCATGCAACAAGGAC
AAATAAGTCATGAAAATTGGGGTGAATTTTCTCTTAATTTACCACCCATGCAACAAGGAGTACAAAATGATGATTTTTCTGCTGAAATTGACTTATGGAA
TCTACTTGATTAA

Fig. S2. Sequence of the gene *ANT1* in *Aubergine* (Abg), *S. lycopersicoides* (Sly) and wild type (WT) plants. Exons are highlighted in grey.

>*ANT1like*[Abg]

```
ATGAACAGTACATCTATGTCTTCTTTGGGAATAAGAAAAGGTTCATGGACTGAAGAAGAAGATTTCCTCTTAAGGAAATGTATCAACAAGTATGGTGAAG
GAAAGTGGCATCTTGTTCCCATAAGAGCTGGTAATTAAACTAACTATTTTGTCTGTCTGTATCATTTTATGTGACATATTTTATAAAATCATGTGAAAAT
GTACGTGCAGGTCTGAATAGATGTCGGAAAAGTTGTAGACTGAGGTGGCTGAATTATCTAAGGCCACATATCAAGAGAGGTGACTTTGAACAAGATGAAG
TGGATCTCATTTTGAGGCTTCATAAGCTCTTAGGCAACAGGTATATATATATATATGCAAGTTTATGTTTTAACATGTGACTATTTCATCTAAACGTTAC
GTTTATTTTACGTAGATGGTCACTTATTGCTGGTAGACTTCCAGGAAGGACAGCAAACGATGTGAAAAACTATTGGAACACAAACCTTCTAAGGAAGTTA
AATACTACTAAAATTGTTGCTCGTGAAAAGATTAAGAGTAAGCGTGGAGAAATTAGTGATAAGATTGAAATAATAAAACCTCAACCTAGGAAGTTCATAT
CGAACACAAAGAAGAATATTACAAGCAATATTGTAATTGAAGACAAAGAGGAACAATGTAAGGAAATAACAAGTGAGAAGCAAACTAGAGATGCATCGAT
AGACAACGGAGATGAATGGTGGGAAAATTTACTGGAAAATTGCAACGACGATGTTGAAGAAGATGAAGAGGTTGTAATTAATTATGAAAAAACACTAACA
AGTTTGTTACATGAGGAAATAACACCACCATTAAATGGTGGAGGTAACTTCATGCAACAAGGACAAAGTGATGGTTGGGATGATTTTTTTGTTGATATTG
ATATATGGGATTTACTTAATTAA
```

>*ANT1like*[Sly]

```
ATGAACAGTACATCTATGTCTTCTTTGGGAATAAGAAAAGGTTCATGGACTGAAGAAGAAGATTTCCTCTTGAGGAAATGTATCAACAAGTATGGTGAAG
GAAAGTGGCATCTTGTTCCCGTAAGAGCTGGTAATTAAACTAACTATTTTGTCTGTCTGTCTCATTTTATGTGACATATTTTATAAAATCATGTGAAAAT
GTACGTGCAGGTCTGAATAGATGTCGGAAAAGTTGTAGACTGAGGTGGCTAAATTATCTAAGGCCACATATCAAGAGAGGTGACTTCGCTTCGGATGAAA
TTGATCTCATTTTGAGACTTCATAAGCTCTTAGGCAACATGTATATATATATATATATATATATATATATATATATATATATATATATATATGCAAGTTT
ATGTTTTAACATGTGACTATTTCATCTAAAGGTTACGTTTATTTTATGCAGATGGTCACTTATTGCTGGTAGACTTCCAGGAAGGACATCTAACGATGTG
AAAAACTATTGGAACACAAACCTTCTAAGGAAGTTAAATACTAGTAAAATTGTTCCTCGTGAAAAGATTAACAATAAGTGTGGAGAAATTAGTACTAAGA
TTGAAATAATAAAACCTCAACCTAGGAAGTTCATATCGAACACAAAGAAGAATATTACAAACAATATTGTAATTGAAGACAAAGAGGAACAATGTAAGGG
AATAATAAGTGAGAAGCAAACTAGAGATTCATCGATAGACAACGGAGATGAATGGTGGGAAAATTTACTGGAAAATTGCAACGACGAAGTTGAAGAAGAT
GAAGAGGTTGTAATTAATTATGAAAAAACACTAACAAGTTTGTTACATGAGGAAACAACACCACCATTAAATGGTGGAGGTAACTTCATGCAACAAGGAC
AAAGTGATGGTTGGGATGATTTTTTTGTTGATAATGATATATGGGATTTACTTAATTAA
```

>*ANT1like*[WT]

```
ATGAACAGTACATCTATGTCTTCTTTGGGAGTAAGAAAAGGTTCATGGACTGAACAAGAAGATCTCCTCTTGAGGAAATGTATCAACAAGTATGGTGAAG
GAAAGTGGCATCTTGTTCCCATAAGAGCTGGTAATTAAACTAACTATCACGCTATTTTATCTGTCTGTCTTATTTTATGTGACATATTTTATAAAATCAC
GTGAAAATATACTTGCAGGTCTGAATAGATGTCGGAAAAGTTGTAGACTAAGGTGGCTAAATTATCTAAGGCCACATATAAAGAGAGGTGACTTCGCTTC
GGATGAAATAGATCTCATTTTGAGGCTTCATAAACTTTTAGGCAACAGGTATATATATATATGCAATTTTTATGTTTTAACATATATATATATGTGATAT
TTCATCCAAATGTTACTTTATTTTATGTAGATGGTCACTTATTGCTGGTAGACTTCCAGGAAGGACAGCAAACGATGTGAAAAACTATTGGAACACAAAC
CTTCTAAGGAAGGTAAATATTACTAAAATTGTTCCACGTGAAAAGTTTAAGAGTAAACGAGGAGAAATTAGTACTAAGATTGAAATAATAAAACCTCAAG
CTAGGAAGTTCATATCGAACACAGAGAAGAATATTACAAACAATATTGTAATTGTAGACAAAGAGGAAGAATGTAAGGAAATAATAAGTGAGAAGCAAAC
TAGAGATGCATCGATAGACAACGGAGATGAATGGTGGGCAAATTTACTGGAAAATTGCAACGACGACGTTGTTGAAGAAGAAGAAGGAGGAGGAGGAGGT
GTAACTGATTATGGAAAAACAATAACAAGTTTGTTACATGAGGAAATAACACCACCATTAAATGGTGGAGGTAACATCATGCAACAAGAACAAAGTGATG
GTTGGGATGATTTTTTTGTTGATATTGATATATGGGATTTACTTAATTAA
```

Fig. S3. Sequence of the gene *ANT1like* in *Aubergine* (Abg), *S. lycopersicoides* (Sly) and wild type (WT) plants. Exons are highlighted in grey.

>*AN2like*^{Abg}

```
ATGAATATTGCCAAGACATTGGGAGTAAGAAAAGGTTCATGGACTGAAGAAGAAGATACTCTTTTGAGGAAATGTATTAACAAGTATGGAGAAGGAAAGT
GGCATCTTGTTCCTTCTAGAGCTGGTAAAGTGAAATTACGATTTTAATTTTATGAATTTTAAATTTTATGATAATAACTAAGTTTTAAATTTATGTAGAT
ATTAAGTAATAATTTGTTAATGCAAAAAATACTATTTAGACAAAATCTATTAGATTATACTAAATTTCCTTTTTAAGAAAAGAGAAACTTAACCTTATGT
TGTGATAGTGGCGTACGAACTCACAACTCTGGCATGAATAGCATTTCATGCCTCCTTTCTTATTACTGAGTCGTCAATCATTTTCGTTAGGAGTTTACAA
GTTAATATAGACATATATTTACTTAATTTTTTAGTCCATATACAATATCTACGAAAAAGTTACTTGATCTGTTCAATCCACAAATCCCACTTACTATTTC
ATACGAATATATGCAGGTCTAAATCGATGTCGAAAGAGTTGTAGACTGAGGTGGTTGAATTATCTAAGGCCACATATCAAGAGAGGTGACTTTGCTCCGG
ATGAAATAGATCTCATTTTAAGACTTCACAAGCTTCTAGGCAATAGGCAAGTCAAAAAATTCGTTAAAAAGTATTCAAAAATTATTATACATATATATTT
AGGAAAAGTAATTTTTGACATATAAATCTGAGTACATACTTCTCTCTCAAATATACTATAGTAAGTGTCTTTGATGCACTATTTTATTTTATTTTTTGGT
TTAGGTGGTCACTTATTGCTGGGAGACTTCCAGGAAGAACAGCAAACGATGTGAAGAACTATTGGAACACACACCTACACAAGAAGTTATTAATAACTCC
TCAGATACAAGAGAATAAGTACAATAATACCCTCAAGATTATCACTGAAAGCACTATACTACGACCACGACCTCGAACCTTTTCAAGTGAAAATAATATT
TCTTGGTGCACTAACAATAGTATGATCACAAACACATTATACAAAGATGACGAACAACGTAACAAAGAAATCGTAGTAAATATTTGTGAGAAGCCAACAA
GAGAAACTCCGTCATCGTCTATAGACGATGACGGAGTTAAATGGTGTACAAATTTACTGGAAAATTGGAAAGAATTTGAGGAAGCAGCAGCAGCAGTATT
GAGCTTTGAGGAAGAAAATAATTTGTTACCAAATTTGTTGAATGAGGGAAATAATTCAACGACCATGCAACATGGAGAAAATGATGACTTTTCAGTTGAT
ATTGACCTATGGAATCTATTTAATTAG
```

>*AN2like*^{Sly}

```
ATGAATATTGCTAAGACATTGGGAGTAAGAAAAGGTTCATGGACTGAAGAAGAAGATTTTCTTTTGAGGAAATGTATTGACAAGTATGGAGAAGGAAAGT
GGCATCTTGTTCCTTCTAGAGCTGGTAAAGTGAAATTACGATTTCAATTTTATGAATTTTAAATTTTATGATAATAACTAAGTTTTAAATTTATGTAGAT
ATTAAGTAATAATTTGTTAATGCAAAAAATACTATTTAGACAAAATCTATTAGATTATACTAAATTTCCTTTTTAAGAAAAGAGAAACTTAACCTTATGT
TGTGATAGTGGCGTACGAACTCACAACTCTAGCATGAATAGCATTTCATGTCTCCTTTCTTATTACTGAGTCGTCAATCATTTTCGTTAGGAGTTTACAA
GTTAATATAGACATATATTTGCTTAATTTTTTAGTCCATATACAATATCTACGAAAAAATTACTGAATCCGTTCAACCCACAAATCCCCACTTACCATTT
CATACAAATATATGCAGGTCTAAATAGATGTCGAAAGAGTTGTAGACTGAGGTGGTTGAATTATCTAAGGCCACATATCAAGAGAGGTGACTTTGCTCCG
GATGAAATAGATCTCATTTTAAGACTTCACAAGCTTCTAGGCAATAGGCAAGTCAAAAATTTCGTTAAAAAGTATTCAAAAATTATTATACATATATATT
TAGGAAAAGTAATTTTTGACATATAAATCTGTGTACATACTTCTCTCTCGAATATACTATAGTAAGTGTCTTTGATGCAATATTTTATTTTATTTTTTGG
TTTAGGTGGTCACTTATTGCTGGGAGACTTCCAGGAAGAACAGCAAACGATGTGAAAAACTATTGGAACACACACCTACACAAGAAGTTAATAACTCCCC
CTCTGATACAAGAGAATAAGTACAATAATACCCTCAAGATTATCACTGAAAGCACTATACTACGACCACGACCACGACCAGGACCTCGACCTCGAACCTT
TTCAAGTGAAAATAATATTTCTTGGTGCACTAACAATAGTATGATCACAAACACATTAGACAAAGATGACGAACAACGTAACAAAGAAATCGTAGTAAAT
ATTTGTGAGAAGCCAACAAGAGAAACTCCGTCATCGTATATAGACGATGACGGAGTTAAATGGTGGACAAATTTACAGGAAAATTGGAAAGAATTTGAGG
AAGAAGCAGCAGCAGTATTGAGCTTTGAGGAAGAAAATAATAAGTTGTTACCAAATTTGTTGTATGAGGGAAATAATTCAACGACCATGCAACATGGAGA
AAATGATGACTTTTCAGTTGATATTGACCTATGGAATCTATTTAATTAG
```

>*AN2like*^{WT}

```
ATGAATATTGCCAAGACATTGGGAGTGAGAAAAGGTTCATGGACTGAAGATGAAGATATTCTTTTGAGGAAATGTATTGACAAGTATGGAGAAGGAAAGT
GGCATCTTGTTCCTTTTAGAGCTGGTAAAGCGAAATTAAGATTTTAGTTTTATAAATTTTAAATTTTATGATAATAAATAAGTTCTAAATTTATGTAGAT
ATTAAGTAATAATTTGTTAATGCAAAAATAATATTTAGGCAAAATCTATTAGATTATACTAAATTTCCTTTTTAAGAAAAGAGAAACTTAACCTTTTGTT
GTGATAGTGGCGTCCCAACCTATAACTCTAGCATGAATAGCATTTCATGCCTCCTTTTTTATTACCGAGTCATAAATCAATTTCGTTAGGAGTTTACAAA
TTAATATACACATATATTTAGTTAAATTTTTTTAGTTCATATATAACATCTACCAAAAAAATTACTGGATTCGTTCAATCCACAAATCCCCACTTACTAT
TATTTCATGTGAATATATGCAGGTCTAAATAGATGTCGAAAGAGTTGTAGACTGAGGTGGTTGAATTATCTAAGGCCACATATCAAGAGAGGTGACTTTG
CTCTGGATGAAATAGATCTCATTTTGAGACTTCACAAGCTTCTAGGCAATAGGCAAATCAAAAATTTCGTTAAAAAATATTTAAAAATTATTGTACATAT
ATATATTCACGAAAAGTAATTTTTGACATATAAATTTACGTACATACTAGTCTCTCGAATATATTATAGTAAGTTATCTTTGATGCCATATTTTTATATT
TTTTTGGTTTAGATGGTCACTTATTGCTGGGAGACTTCCTGGAAGAACAGCAAACGATGTGAAAAACTATTGGAACACACACCTACACAAGAAGTTATTA
ATAACTCCTCCTCAGATACAAGAGAATAAGTACAATAATACCCTCAAGATTATCACTGAAAGCACTATACTACGACCACGACCAAGACCAGGACCTCAAC
CTCGAACCTTCTCAAGTGAAAATAATATTTCTTGGTGCACTAACAATAGTATGATCACAAACACATTAGACAAAGATGACGAACAACACAACAAAGAAAT
CGCAGTAAATATTTGTGAGAAGCCAACAAAAAAAAACACCGTCATCGTCTATAGACGATGATGGAGTTCAATGGTGGACAAATTTACTGGAAAATTGGAAA
GAATTTGAGGAAGAAGCAACAGCAGTATTGAACTTTGAGGAAGAAAATAAGTTGTTGCCAAATTTGTTGTATGAGGAACATAATTCAACAACCATGCAAC
ATGGAGAAAATGATGACTTTTCAGTTGATATTGACCTATGGAATCTATTTAATTAG
```

>*AN2like*^{Aft}

```
ATGAATATTGCCAAGACATTGGGAGTGAGAAAAGGTTCATGGACTGAAGATGAAGATATTCTTTTGAGGAAATGTATTGACAAGTATGGAGAAGGAAAGT
GGCATCTTGTTCCTTTTAGAGCTGGTAAAGCAAAATTAAGATTTTAATTTTATGTATTTTAAATTTTATGATAATAATTAAGTTTTAAATTTATGTAGAT
TTTAAGTAAAATTTGTTAATGCAAAAATACTATTTAGGCAAAATCTGTTAGATTATACTAAATTTCCTTTTTAAGAAAAGAGAAACTTACCTTTTGTTGT
GATAGTGGCGTCCCAACCTATAACTCTAGCATGAATAGCATTTCATGCCTCCTTTTTTATTACTGAGTCGTAAATTAATTTTGGTAGGAGTTTACAAGTT
AATATATATATATATTTGATTAATTTTTTTAGTTTATATACAATATCTATGAAAAAATTACTAGGTTCGTTCAACCCACAAATCCCCACTTACTATTATT
TCACGTGATTATATGCAGGTCTAAATAGATGTCGAAAGAGTTGTAGACTGAGGTGGTTGAATTATCTAAGGCCACATATCAAGAGAGGTGACTTTGCTAT
GGATGAAATAGATCTCATTTTGAGACTTCACAAGCTTCTAGGCAATAGGCAAGTCAGAAATTTAGTTAAAAGAAATTCAAAAATTATTGTACATATATAT
TCACGAAAAGAAACTTTTGACATACAAATTTGTGTACATACTAGTCTTCCGTATATATTATAGTAAGTTGTCTTTGATGCCATATTTTTATTTTCTTTTG
GTTTAGATGGTCACTTATTGCTGGGAGACTTCCGGGAAGAACAGCAAACGATGTGAAAAACTATTGGAACACACACCTACACAAGAAGTTATTAATAACT
CCTCAGATACAAGAGAATAAGTACAATAAAACCCTCAAGATTATCACTGAAAGCACTATACTACGACCACGACCAAGACCTCGACCTCGAACATTCTCAA
GTGAAAATAATATTTCTTGGTGCACTAACAATAGTATGATCACAAACACATTAGACAAAGATGACGAACAACGCAACAAAGAAATCGCAGTAAATATTTG
TGAGAAGCCAACAAGAGAAACACCGTCATCGTCTATAGACGATGATGGAGTTAAATGGTGGACAAATTTACTGGAAAATTGGAAAGAATTTGAGGAAGAA
GCAACAGCAGTATTGAACTTTGAGGAAGAAAATAAGTTGTTACCAAATTTGTTGTGTGAGGAACATAATTCAACAACCATGCAACATGGAGAAAATGATG
ACTTTTCAGTTGATATTGACCTATGGAATCTATTTAATTAG
```

Fig. S4. Sequence of the gene *AN2like* in *Aubergine* (Abg), *S. lycopersicoides* (Sly), wild type (WT) and *Anthocyanin fruit* (Aft) plants. Exons are highlighted in grey.

```
AN2WT         ATGAATACTCCTATGTGTGCATCGTTGGGAGTTAGGAAAGGTTCATGGACTGAACAAGAA      60
AN2Sly        ATGAATACTCCTATGTGTGCATCGTTGGGAGTTAGGAAAGGTTCATGGACTGAACAAGAA      60
AN2Abg        ATGAATACTCCTATGTGTGCATCGTTGGGAGTTAGGAAAGGTTCATGGACTGAACAAGAA      60
              ************************************************************

AN2WT         GATTCTCTTTTAAGAGATTGCATTCAAAAATATGGTGAAGGAAAGTGGCATCTTGTTCCT      120
AN2Sly        GATTTTCTTTTAAGAAATTGCATTCAAAAATATGGTGAAGGGAAGTGGCATCTTGTTCCT      120
AN2Abg        GATTTTCTTTTAAGAAATTGCATTCAAAAATATGGTGAAGGAAAGTGGCATCTTGTTCCT      120
              **** ********** ************************* ******************

AN2AC         GCTAGAGCTGGTATTACTTTTGTACTTTTTCTAATTTGTTTTAAAAAATAATGTATTTTA      180
AN2Sly        GCTAGAGCTGGTATTACATTTTTTTCTTTTTTAGTTCATTTTAAAAAAAATATTTTTACA      180
AN2Abg        GCTAGAGCTGGTATTACATTTATTTATTTT-TAGTTCGTTTAAAAAAAATATATTTTTAT      179
              ***************** *** *   **** ** **  *** ******     * **

AN2WT         TATATTTATAAATGATTTACTAAATTCTAAGTTTTGATTTTACTTTCTAATAACATAATT      240
AN2Sly        TAT----AAAAATAATTTATTAAACTTTAAATTTTGATTT-TACTTTTAATAACATGATT      235
AN2Abg        ATT----TAAAATAATTTATTAAACTTTAAATTTTGATTT-GACTTCTAATAACATAATT      234
                *      **** ***** **** * *** *********    ** ********* ***

AN2WT         TATAGTTTTATGATTTGTTTTAAATCATAAATTTTTGTAATATTTTATATTTATTTATTA      300
AN2Sly        TATAGTTTTATGATTTGTTTTAAATCATAAGTTT---------------------TATAA      274
AN2Abg        AATAGTTTTATGATTTATTTTAAATCATAAGTTT---------------------TATAC      273
               *************** ************* ***                     ***

AN2AC         ATTTTGAATGTTATGTGAACATGTATAGGTTTAAATCGATGTCGAAAGAGTTGCAGACTA      360
AN2Sly        TATTTTAATTTAATGTGAATATGTGTAGGTTTAAATCGATGTCGAAAAAGTTGCAGACTA      334
AN2Abg        TCCCTTAATTTAATGTGAATATGTGTAGGTTTGAATCGATGTCGAAAGAGTTGCAGACTA      333
                  * *** * ******* **** ******* ************** ************

AN2WT         AGGTGGCTAAACTATCTAAGGCCACATATCAAGAGAGGTGACTTTGCTCCAGATGAAGTG      420
AN2Sly        AGGTGGCTAAATTATCTAAGGCCACATATCAAGAGAGGTGACTTTGCTCCGGATGAAGTT      394
AN2Abg        AGGTGGCTAAATTATCTAAGGCCACATATCAAGAGAGGTGACTTTGCTCCAGATGAAGTA      393
              *********** ************************************** ********

AN2WT         GATCTCATCTTGAGACTTCACAAACTCTTAGGCAATAGGTAAGTCTATGCTGCAATTATT      480
AN2Sly        GATCTCATCTTGAGACTTCATAAACTCTTAGGCAATAGGTAAGTCTATGTATTCAGTGGA      454
AN2Abg        GATCTCATCTTGAGACTTCATAAACTCTTAGGCAATAGGTAAGTCTATGTATTTAGTGGA      453
              ******************** ****************************     * *

AN2WT         TCATTCAAAAGTTTTTGATGTTATATTTTTTTTTATATTGTATTGGTCAAATATATAAGT      540
AN2Sly        TTGCAATCATTTCATTCAAAAGCTTTTAATATT------AAAAAAATATGATATATTCTC      508
AN2Abg        TTGCAATCATTCCATTCAAAAGCTTTTAATATT------ACAAAGTTATGATATACTCCG      507
              *       *     ** *     * **  * **        *    *   *****

AN2WT         AGATTTTTAAATTTGTTGGATTTTCTTCCTCAGATATTTCAATTACGTCATTTTTCTGTA      600
AN2Sly        TTCGTTTT------------------AAAAAAAATAATTTCATTTTCTTTTTAATCTGTT      550
AN2Abg        TCTGTTTT------------------TTAAAAAAAGATCTCATTTCTTTTTCAGTCTGTT      549
                  ****                       * *   *   ***   *  *   *****

AN2WT         TAATCATTGAACTCCCATAATTTGTTCCATTTAAACAATCATCGTTGTCTGATGTGGATC      660
AN2Sly        TAAAAAAG--AATGACATT---TTTTCTTTTTTGGCAAAACT------------------      587
AN2Abg        TAAAAAAG--AATAAA-------AAAATTTTTTGACAAAACT------------------      582
              ***  *    * *                ***   ***   *

AN2WT         AAGATTTGTGAGGGTATTGTTTCACAAATCATAGTCCAATTGATAGTGAAAATGTTTGAG      720
AN2Sly        -------TTAATTTTAACTTTTCACGTGGCATATTTAA------GACCACAAGATTAAAA      634
AN2Abg        -------TTAATTTTAACTTTTCACGTGACATGTTTAA------GACCACAAGATTAAAA      629
                      * *   **   ******    ***  *  *          * **  **  *

AN2WT         AATAATTGTGTTTTACTGAAGTCATTTTGAACACATTAGAAAACAAATGAGACTAGCACG      780
AN2Sly        GATATTTTAATACATTTGACATAACTTTAA-----TTAAGAATCACAAGATAA--AAA--      685
AN2Abg        GACATTTTAATACATTTGACATAACTTTAA-----TTTAGAACCACAAGATAA--AAA--      680
               * * **   *     ***  * * *** *     **   ** ** * ** *     *

AN2WT         GTTTGTCTTCATTCCTTCTATCAAAATTATTACAACTCAAACACAATCGAAGACATTTAC      840
AN2Sly        --AATTCTTATTTCTTTTCTTAAACCCCGTTCCAACTCAAACTAGACC------A-TTCT      736
AN2Abg        --A-ATCTTATTTCTTTTCTTAAACTTCGTTCCAAGTCAAACTAAACC------A-TTTT      730
                   ****  *** **   * **     ** *** ******   * *      * **

AN2WT         AATAGAAAAGCTGATCAAAAGTGTTTTAAAAAAACAAATTATGGGTGGTTCAATGATTCA      900
AN2Sly        TTTTGAAACGGAGAGAGTAATT----------------------TTCTTTATATTAT---      771
AN2Abg        TTTTAAAACAGAGGGAGTAATT----------------------TTCTTTATATTAT---      765
                *  ***    *     ** *                       *  **  ** **

AN2WT         ATTGAAAAATGACGTAATTGAGGTACCTGAGAAAAAAAAACCTAAACATATGTGTAATTA      960
AN2Sly        -----------ATTTTATATATATAT---ATGTATAACCATATCTTAATACGTGTAATTA      817
AN2Abg        -----------ATTA--TATATATAT---ATGTATAACCATATCTTAATATGTGTAATTA      809
                         *     *  *  **    *   * **  *  *    *** *********
```

```
AN2^WT     TTTCA-TTTAAAAGTTAGTTATTTTGTATAGGTGGTCACTTATTGCTGGTAGACTTCCAG      1019
AN2^Sly    TTTCATTTAAAAGTTACGCTATTTTGTGTAGGTGGTCACTTATTGCTGGTAGACTTCCAG      877
AN2^Abg    TTTCATTTAAAAGTTACGCTATTTTGTGTAGGTGGTCACTTATTGCTGGTAGACTTCCAG      869
           ***** ** ***  *  * ******** ********************************

AN2^WT     GAAGGACAGCAAACGATGTGAAGAACTATTGGAATACACACTTTCACAAGAAGTTAAGTA      1079
AN2^Sly    GAAGGACTGCAAATGATGTGAAGAACTATTGGAACACTCACTTTCATAAGAAGTTAAGTA      937
AN2^Abg    GAAGGACTGCAAATGATGTTAAGAACTATTGGAACACTCACTTTCATAAGAAGTTAAATA      929
           ******* ***** ***** ************** ** ******** ********** **

AN2^WT     TTATTGCTCCTCATCTACATCCTCATTCTCGTCCTCGTTCTCATCCTCGTCTACAGATTA      1139
AN2^Sly    TTATTGCTCCTCATCTTCATCCTCGTCCTCGTCCTCGTCCTCATCCTCATCTACAGATTA      997
AN2^Abg    TTATTGCTCCTCATCTTCATCCTCGTCCT------CGTCCTCATCCTCATCTACAGATTA      983
           **************** ******* * **      *** ********* ***********

AN2^WT     AGCATAAGAGCATCGCGGTTACTAAGAATGAAATAATAAGACCTCAACCTCGGAACTTCT      1199
AN2^Sly    ATCATAAGAGCATCGCGGTTACTAAGAATGAAATAATAAAACCTCAACCTCGGAACTTCT      1057
AN2^Abg    AGCATAAGAGCATCGCGGTTACTAAGAATGAAATAATAAAACCTCAACCTCGGAACTTCT      1043
           * ************************************* ********************

AN2^WT     CAAACGTTAAGAAGAATGATTCTCATTGGTGCAAC---AACAAAAGTATGATCACAAACA      1256
AN2^Sly    CAAACATTAATAAGAATATTTCTCATTGGTGCAACAACAACAAAAGTATGATCACAAACA      1117
AN2^Abg    CAAACGTTAATAAGAATATTTCTCATTGGTGCAACAACAACAAAAGTATGATCACAAACA      1103
           ***** **** ******  ****************   **********************

AN2^WT     CATTAGACAAAGATGACAAACGTTGCAACGAAATCGTTGTAAATATTTGTGAGAAGCCAA      1316
AN2^Sly    CATTAGACAAAGATGACAAACGTTGCAAGGAGATGGTAGTAAATATTAGTGAGAAGCCAA      1177
AN2^Abg    CATTAGACAAAGATGACAAACGTTGCAAGGAGATGGTAGTAAATATTTGTGAGAAGCCAA      1163
           **************************** ** ** ** ********* ************

AN2^WT     TAGGAGAAAATACATCGTCGATAGACGATGGAGTTGAATGGTGGACAAATTTACTGGAAA      1376
AN2^Sly    TAGGAGAAAATACATCGTCGATAGACGATGGAGTTGAATGGTGGACAAATTTACTGGAAA      1237
AN2^Abg    TAGGAGAAAATACATCGTCGATAGACGATGGAGTTGAATGGTGGACAAATTTACTGGTAA      1223
           ********************************************************* **

AN2^WT     ATTGCATTGAAATTGAAGAAGAAACAGCTAATACAAATTTTGGAAAAACACCAACAATGT      1436
AN2^Sly    ATTGCAATGAAATTGAAGAAGAAGCAGTTGTTACAAATTTTGAAAAAACACCAACAATGT      1297
AN2^Abg    ATTGCAATGAAATTGAAGAAGAAGCTGTTGTTACAAATTTTGAAAAGACACCAACAATGT      1283
           ****** **************** * * *  *********** *** *************

AN2^WT     TGTTACATGAGGAAATATCACCACCGTTAGTTAAT---GGTGAAGACAACTCCATGCAAC      1493
AN2^Sly    TGTTACATGAGGAAATATCACCACCGTTAATAATTAATGGTGAAGGCAACTCCATGCAAC      1357
AN2^Abg    TGTTAAATGAGGAAATATCACCACCGTTAATAATTAATGGTGAAGGCAACTCCATGCAAC      1343
           ***** *********************** * * *   ******* **************

AN2^WT     AAGGACCA---ACTAATAATTGGGATGACTTTTCAACTGATATTGACTTATGGAATCTAC      1550
AN2^Sly    AAGGACAAACTCATGATAATTGGGATGACTTTTCAACTGATATTGACTTATGGAATCTAC      1417
AN2^Abg    AAGGACAAACTCATGATAATTGGGATGACTTTTCAACTGATATTGACTTATGGAATCTAC      1403
           ****** *     * *********************************************

AN2^WT     TTAATTAA 1558
AN2^Sly    TTAATTAA 1425
AN2^Abg    TTAATTAA 1411
           ********
```

Fig. S5. ClustalW alignment of the gene *AN2* sequenced in *Aubergine* (Abg), *S. lycopersicoides* (Sly) and wild type (WT). Exons are highlighted in grey. The symbol * indicates perfect alignment.

```
ANT1^WT     ATGAACAGTACATCTATGTCTTCATTGGGAGTGAGAAAAGGTTCATGGACTGATGAAGAA     60
ANT1^Sly    ATGAACAGTACATCTATGTCTTCATTGGGAGTGAGAAAAGGTTCTTGGACTGATGAAGAA     60
ANT1^Abg    ATGAACAGTACATCTATGTCTTCATTGGGAGTGAGAAAAGGTTCTTGGACTGATGAAGAA     60
            ******************************************** ***************

ANT1^WT     GATTTTCTTCTAAGAAAATGTATTGATAAGTATGGTGAAGGAAAATGGCATCTTGTTCCC     120
ANT1^Sly    GATTTTCTTTTAAGAAAATGTATTAACAAGTATGGTGAAGGAAAATGGCATCTTGTTCCC     120
ANT1^Abg    GATTTTCTTTTAAGAAAATGTATTAACAAGTATGGTGAAGGAAAATGGCATCTTGTTCCC     120
            ********* ************** * *********************************

ANT1^WT     ATAAGAGCTGGTAACCTATTAAATTAACTATCACGTTATTTTTATTTGTCTTTCTGTCTC     180
ANT1^Sly    ATGAGAGCTGGTAATTAAATTAACTATC---------GCATTATTTTATCTGTCTGTCTC     171
ANT1^Abg    ATGAGAGCTGGTAATTAAATTAACTATC---------GCATTATTTTATCTGTCTGTCTC     171
            ** ***********   * * ** ** *            **  *** *** ********

ANT1^WT     ATTTTATTTGACGTTATTACGAATATCATCTGAAAATGTACGTGCAGGTCTGAATAGATG     240
ANT1^Sly    ATTTTATATGATGTTATTTAAAAAATTATGTGAAAATGTATGTGCAGGTCTGAATAGATG     231
ANT1^Abg    ATTTTATATGATGTTATTTAAAAAATTATGTGAAAATGTATGTGCAGGTCTGAATAGATG     231
            ******* *** ******   ** ** ** ********** *******************

ANT1^WT     TCGGAAAAGTTGTAGATTGAGGTGGCTGAATTATCTAAGGCCACATATCAAGAGAGGTGA     300
ANT1^Sly    TCGGAAAAGTCGTAGATTGAGGTGGCTGAATTATCTAAGGCCACATATCAAGAGAGGTGA     291
ANT1^Abg    TCGGAAAAGTTGTAGATTGAGGTGGCTGAATTATCTAAGGCCACATATCAAAAGAGGTGA     291
            ********** ***************************************** ********

ANT1^WT     CTTTGAACAAGATGAAGTGGATCTCATTTTGAGGCTTCATAAGCTCTTAGGCAACAGGCA     360
ANT1^Sly    CTTTGAACAAGATGAAGTGGATCTCATTTTGAGGCTTCATAAGCTCTTAGGCAACAGGCA     351
ANT1^Abg    CTTTGAACAAGATGAAGTGGATCTCATTTTGAGGCTTCATAAGCTCTTAGGCAACAGGCA     351
            ************************************************************

ANT1^WT     TGCAAGTTTATGTTTTGACAAAATTTGATTAGTATATATTATATATACGTGTGACTATTT     420
ANT1^Sly    TGCAATTTTATGTTTTGACAAA-TTTGATTAATATAATATATATATATGTGTGACTATTT     410
ANT1^Abg    TGCAATTTTATGTTTTGACAAA-TTTGATTAATATAATATATATATTTGTGTGACTATTT     410
            ***** **************** ******** ****   *******  ************

ANT1^WT     CATCTAAATGTTACGTTATTTTACGTAGATGGTCACTTATTGCTGGTAGACTTCCCGGAA     480
ANT1^Sly    CATCTAAACTTTACGTTATGTTATGCAGATGGTCACTTATTGCAGGTAGACTTCCAGGAA     470
ANT1^Abg    CATCTAAACGTTACGTTATTTTATGCAGATGGTCACTTATTGCAGGTAGACTTCCAGGAA     470
            ********  ********* *** * ***************** *********** ****

ANT1^WT     GGACAGCTAACGATGTGAAAAACTATTGGAACACTAATCTTCTAAGGAAGTTAAATACTA     540
ANT1^Sly    GGACAGCTAACGACATAAAAAACTATTGGAACACTAACCTTCTAAGGAAGTTAAATACTA     530
ANT1^Abg    GGACAGCTAACGACATAAAAAACTATTGGAACACTAACCTTCTAAGGAAGTTAAATACTA     530
            *************  * ******************** **********************

ANT1^WT     CTAAAATTGTTCCTCGCGAAAAGATTAACAATAAGTGTGGAGAAATTAGTACTAAGATTG     600
ANT1^Sly    GTAAAATTGTTCCTCGTGAAAAGATTAACAATAAGTGTGGAGAAATTAGTACTAAGATTG     590
ANT1^Abg    GTAAAATTGTTCCTCGTGAAAAGATTAACAATAAGTGTGGAGAAATTAGTACTAAGATTG     590
             *************** *******************************************

ANT1^WT     AAATTATAAAACCTCAACGACGCAAGTATTTCTCAAGCACAATGAAGAATGTTACAAACA     660
ANT1^Sly    AAATAATAAAACCTCAACCTAGGAAGTATTTCTCAAGCACAAAGACGAA-----------     639
ANT1^Abg    AAATAATAAAACCTCAACCTAGGAAGTATTTCTCAAGCACAAAGATGAA-----------     639
            **** *************   * ******************* ** ***

ANT1^WT     ATAATGTAATTTTGGACGAGGAGGAACATTGCAAGGAAATAATAAGTGAGAAACAAACTC     720
ANT1^Sly    -TATTGTAATTTTGGATGAGGAGGAACATTGCAAGGAAATAATAAGTGAGAAGCAAACTC     698
ANT1^Abg    -TATTGTAATTTTTGATGAGGAGGAACATTGCAAGGAAATAATAAGTGAGAAGCAAACTC     698
             ** ********* ** *********************************** *******

ANT1^WT     CAGATGCATCGATGGACAACGTAGATCCATGGTGGATAAATTTACTGGAAAATTGCAATG     780
ANT1^Sly    CAGATGCATCGATGGACAACGTAGATCAATGGTGGACAAATTTAGTGGAAAATTGCAATG     758
ANT1^Abg    CAGATGCATCGATGGACAACGTAGATCAATGGTGGACAAATTTACTGGAAAATTGCAATG     758
            *************************** ******** ******* ***************

ANT1^WT     ACGATATTGAAGAAGATGAAGAGGTTGTAATTAATTATGAAAAAACACTAACAAGTTTGT     840
ANT1^Sly    ACGATGTTGAAGAAGATGAAGAGGTTGTAATTAATTATGAAAAAACACTAACAAGTTTGT     818
ANT1^Abg    ACGATGTTGAAGAAGATGAAGAGGTTGTAATTAATTATGAAAAAACACTAACAAGTTTGT     818
            ***** ******************************************************

ANT1^WT     TACATGAAGAAATATCACCACCATTAAATATTGGTGAAGGTAACTCCATGCAACAAGGAC     900
ANT1^Sly    TACATGAAGAAATATCACCACCATTAAATGGTGAAGGTAACTCTATG---CAACAAGGAC     875
ANT1^Abg    TACATGAAGAAATATCACCACCATTAAATGGTGAAGGTAACTCTATAATGCAACAAGGAC     878
            *****************************  **  *              **********

ANT1^WT     AAATAAGTCATGAAAATTGGGGTGAATTTTCTCTTAATTTACCACCCATGCAACAAGGAG     960
ANT1^Sly    AAACAAGTCATGATAGTTGGGGTGACTTTTCTCTTAATTTACCACCCATGCAACAAGGAG     935
ANT1^Abg    AAACAAGTCATGATAGTTGGGGTGACTTTTCTCTTAATTTACCACCCATGCAACAAGGAG     938
            *** ********* * ********* **********************************
```

```
ANT1^WT    TACAAAATGATGATT---------TTTCTGCTGAAATTGACTTATGGAATCTACTTGATT    1011
ANT1^Sly   TACAAAATGATGATTGGGATGATTTTTCTGCTGAAATTGACTTATGGAATCTACTTGATT    995
ANT1^Abg   TACAAAATGATGATTGGGATGATTTTTCTGCTGAAATTGACTTATGGAATCTACTTGATT    998
           ***************         ************************************

ANT1^WT    AA             1013
ANT1^Sly   AA              997
ANT1^Abg   AA             1000
           **
```

Fig. S6. ClustalW alignment of the gene *ANT1* sequenced in *Aubergine* (Abg), *S. lycopersicoides* (Sly) and wild type (WT) plants. Exons are highlighted in grey. The symbol * indicates perfect alignment.

```
ANT1likeWT    ATGAACAGTACATCTATGTCTTCTTTGGGAGTAAGAAAAGGTTCATGGACTGAACAAGAA  60
ANT1likeSly   ATGAACAGTACATCTATGTCTTCTTTGGGAATAAGAAAAGGTTCATGGACTGAAGAAGAA  60
ANT1likeAbg   ATGAACAGTACATCTATGTCTTCTTTGGGAATAAGAAAAGGTTCATGGACTGAAGAAGAA  60
              ****************************** *********************** *****

ANT1likeWT    GATCTCCTCTTGAGGAAATGTATCAACAAGTATGGTGAAGGAAAGTGGCATCTTGTTCCC  120
ANT1likeSly   GATTTCCTCTTGAGGAAATGTATCAACAAGTATGGTGAAGGAAAGTGGCATCTTGTTCCC  120
ANT1likeAbg   GATTTCCTCTTAAGGAAATGTATCAACAAGTATGGTGAAGGAAAGTGGCATCTTGTTCCC  120
              *** ******* ************************************************

ANT1likeWT    ATAAGAGCTGGTAATTAAACTAACTATCACGCTATTTTATCTGTCTGTCTTATTTTATGT  180
ANT1likeSly   GTAAGAGCTGGTAATTAAACTAACT--------ATTTTGTCTGTCTGTCTCATTTTATGT  172
ANT1likeAbg   ATAAGAGCTGGTAATTAAACTAACT--------ATTTTGTCTGTCTGTATCATTTTATGT  172
               ************************        ***** ********* * *********

ANT1likeWT    GACATATTTTATAAAATCACGTGAAAATATACTTGCAGGTCTGAATAGATGTCGGAAAAG  240
ANT1likeSly   GACATATTTTATAAAATCATGTGAAAATGTACGTGCAGGTCTGAATAGATGTCGGAAAAG  232
ANT1likeAbg   GACATATTTTATAAAATCATGTGAAAATGTACGTGCAGGTCTGAATAGATGTCGGAAAAG  232
              ******************* ******** *** ***************************

ANT1likeWT    TTGTAGACTAAGGTGGCTAAATTATCTAAGGCCACATATAAAGAGAGGTGACTTCGCTTC  300
ANT1likeSly   TTGTAGACTGAGGTGGCTAAATTATCTAAGGCCACATATCAAGAGAGGTGACTTCGCTTC  292
ANT1likeAbg   TTGTAGACTGAGGTGGCTGAATTATCTAAGGCCACATATCAAGAGAGGTGACTTTGAACA  292
              ********* ******** ******************** ************** *

ANT1likeWT    GGATGAAATAGATCTCATTTTGAGGCTTCATAAACTTTTAGGCAACAGGTATATATATAT  360
ANT1likeSly   GGATGAAATTGATCTCATTTTGAGACTTCATAAGCTCTTAGGCAACATGTATATATATAT  352
ANT1likeAbg   AGATGAAGTGGATCTCATTTTGAGGCTTCATAAGCTCTTAGGCAACAGGTATATATA---  349
               ****** * ************** ******** ** ********** *********

ANT1likeWT    ATGCAATTTTTATGTTTTA-----ACATATATA------------------------TAT  391
ANT1likeSly   ATATATATATATATATATATATATATATATATATATATATATGCAAGTTTATGTTTTAACAT  412
ANT1likeAbg   ---------------------------------TATATATGCAAGTTTATGTTTTAACAT  376
                                                                        **

ANT1likeWT    ATGTGATATTTCATCCAAATGTTACTTTATTTTATGTAGATGGTCACTTATTGCTGGTAG  451
ANT1likeSly   GTGACTATTTCATCTAAAGGTTACGTTTATTTTATGCAGATGGTCACTTATTGCTGGTAG  472
ANT1likeAbg   GTGACTATTTCATCTAAACGTTACGTTTATTTTACGTAGATGGTCACTTATTGCTGGTAG  436
               **     **      **   *   ********* * ***********************

ANT1likeWT    ACTTCCAGGAAGGACAGCAAACGATGTGAAAAACTATTGGAACACAAACCTTCTAAGGAA  511
ANT1likeSly   ACTTCCAGGAAGGACATCTAACGATGTGAAAAACTATTGGAACACAAACCTTCTAAGGAA  532
ANT1likeAbg   ACTTCCAGGAAGGACAGCAAACGATGTGAAAAACTATTGGAACACAAACCTTCTAAGGAA  496
              **************** * *****************************************

ANT1likeWT    GGTAAATATTACTAAAATTGTTCCACGTGAAAAGTTTAAGAGTAAACGAGGAGAAATTAG  571
ANT1likeSly   GTTAAATACTAGTAAAATTGTTCCTCGTGAAAAGATTAACAATAAGTGTGGAGAAATTAG  592
ANT1likeAbg   GTTAAATACTACTAAAATTGTTGCTCGTGAAAAGATTAAGAGTAAGCGTGGAGAAATTAG  556
              * ****** ** ********** * ********* **** * ***  * ***********

ANT1likeWT    TACTAAGATTGAAATAATAAAACCTCAAGCTAGGAAGTTCATATCGAACACAGAGAAGAA  631
ANT1likeSly   TACTAAGATTGAAATAATAAAACCTCAACCTAGGAAGTTCATATCGAACACAAAGAAGAA  652
ANT1likeAbg   TGATAAGATTGAAATAATAAAACCTCAACCTAGGAAGTTCATATCGAACACAAAGAAGAA  616
              *  ************************* *********************** *******

ANT1likeWT    TATTACAAACAATATTGTAATTGTAGACAAAGAGGAAGAATGTAAGGAAATAATAAGTGA  691
ANT1likeSly   TATTACAAACAATATTGTAATTGAAGACAAAGAGGAACAATGTAAGGGAATAATAAGTGA  712
ANT1likeAbg   TATTACAAGCAATATTGTAATTGAAGACAAAGAGGAACAATGTAAGGAAATAACAAGTGA  676
              ******** ************** ************* ********* ***** ******

ANT1likeWT    GAAGCAAACTAGAGATGCATCGATAGACAACGGAGATGAATGGTGGGCAAATTTACTGGA  751
ANT1likeSly   GAAGCAAACTAGAGATTCATCGATAGACAACGGAGATGAATGGTGGGAAAATTTACTGGA  772
ANT1likeAbg   GAAGCAAACTAGAGATGCATCGATAGACAACGGAGATGAATGGTGGGAAAATTTACTGGA  736
              **************** ****************************** ************

ANT1likeWT    AAATTGCAACGACGACGTTGTTGAAGAAGAAGAAGGAGGAGGAGGAGGTGTAACTGATTA  811
ANT1likeSly   AAATTGCAACGACGAAGTTGAAGAAGATGA------------AGAGGTTGTAATTAATTA  820
ANT1likeAbg   AAATTGCAACGACGATGTTGAAGAAGATGA------------AGAGGTTGTAATTAATTA  784
              *************** ****  ***** **            **  * ***** * ****

ANT1likeWT    TGGAAAAACAATAACAAGTTTGTTACATGAGGAAATAACACCACCATTAAATGGTGGAGG  871
ANT1likeSly   TGAAAAAACACTAACAAGTTTGTTACATGAGGAAACAACACCACCATTAAATGGTGGAGG  880
ANT1likeAbg   TGAAAAAACACTAACAAGTTTGTTACATGAGGAAATAACACCACCATTAAATGGTGGAGG  844
              ** ******* ************************ ************************
```

```
ANT1like^WT     TAACATCATGCAACAAGAACAAAGTGATGGTTGGGATGATTTTTTTGTTGATATTGATAT  931
ANT1like^Sly    TAACTTCATGCAACAAGGACAAAGTGATGGTTGGGATGATTTTTTTGTTGATAATGATAT  940
ANT1like^Abg    TAACTTCATGCAACAAGGACAAAGTGATGGTTGGGATGATTTTTTTGTTGATATTGATAT  904
                **** ************ *********************************** ******

ANT1like^WT     ATGGGATTTACTTAATTAA    950
ANT1like^Sly    ATGGGATTTACTTAATTAA    959
ANT1like^Abg    ATGGGATTTACTTAATTAA    923
                *******************
```

Fig. S7. ClustalW alignment of the gene *ANT1like* sequenced in *Aubergine* (Abg), *S. lycopersicoides* (Sly) and wild type (WT) plants. Exons are highlighted in grey. The symbol * indicates perfect alignment.

```
AN2like^WT     ATGAATATTGCCAAGACATTGGGAGTGAGAAAAGGTTCATGGACTGAAGATGAAGATATT     60
AN2like^Aft    ATGAATATTGCCAAGACATTGGGAGTGAGAAAAGGTTCATGGACTGAAGATGAAGATATT     60
AN2like^Abg    ATGAATATTGCCAAGACATTGGGAGTAAGAAAAGGTTCATGGACTGAAGAAGAAGATACT     60
AN2like^Sly    ATGAATATTGCTAAGACATTGGGAGTAAGAAAAGGTTCATGGACTGAAGAAGAAGATTTT     60
               *********** ************** *********************** ******  *

AN2like^WT     CTTTTGAGGAAATGTATTGACAAGTATGGAGAAGGAAAGTGGCATCTTGTTCCTTTTAGA     120
AN2like^Aft    CTTTTGAGGAAATGTATTGACAAGTATGGAGAAGGAAAGTGGCATCTTGTTCCTTTTAGA     120
AN2like^Abg    CTTTTGAGGAAATGTATTAACAAGTATGGAGAAGGAAAGTGGCATCTTGTTCCTTCTAGA     120
AN2like^Sly    CTTTTGAGGAAATGTATTGACAAGTATGGAGAAGGAAAGTGGCATCTTGTTCCTTCTAGA     120
               ****************** ************************************ ****

AN2like^WT     GCTGGTAAAGCGAAATTAAGATTTTAGTTTTATAAATTTTAAATTTTATGATAATAAATA     180
AN2like^Aft    GCTGGTAAAGCAAAATTAAGATTTTAATTTTATGTATTTTAAATTTTATGATAATAATTA     180
AN2like^Abg    GCTGGTAAAGTGAAATTACGATTTTAATTTTATGAATTTTAAATTTTATGATAATAACTA     180
AN2like^Sly    GCTGGTAAAGTGAAATTACGATTTCAATTTTATGAATTTTAAATTTTATGATAATAACTA     180
               **********  ****** ***** * ******  ********************** **

AN2like^WT     AGTTCTAAATTTATGTAGATATTAAGTAATAATTTGTTAATGCAAAAA-TAATATTTAGG     239
AN2like^Aft    AGTTTTAAATTTATGTAGATTTTAAGTAA-AATTTGTTAATGCAAAAA-TACTATTTAGG     238
AN2like^Abg    AGTTTTAAATTTATGTAGATATTAAGTAATAATTTGTTAATGCAAAAAATACTATTTAGA     240
AN2like^Sly    AGTTTTAAATTTATGTAGATATTAAGTAATAATTTGTTAATGCAAAAAATACTATTTAGA     240
               **** *************** ******** ****************** ** ******* 

AN2like^WT     CAAAATCTATTAGATTATACTAAATTTCCTTTTTAAGAAAAGAGAAACTTAACCTTTTGT     299
AN2like^Aft    CAAAATCTGTTAGATTATACTAAATTTCCTTTTTAAGAAAAGAGAAACTTA-CCTTTTGT     297
AN2like^Abg    CAAAATCTATTAGATTATACTAAATTTCCTTTTTAAGAAAAGAGAAACTTAACCTTATGT     300
AN2like^Sly    CAAAATCTATTAGATTATACTAAATTTCCTTTTTAAGAAAAGAGAAACTTAACCTTATGT     300
               ******** ****************************************** **** ***

AN2like^WT     TGTGATAGTGGCGTCCCAACCTATAACTCTAGCATGAATAGCATTTCATGCCTCCTTTTT     359
AN2like^Aft    TGTGATAGTGGCGTCCCAACCTATAACTCTAGCATGAATAGCATTTCATGCCTCCTTTTT     357
AN2like^Abg    TGTGATAGTGGCGTACGAACTCACAACTCTGGCATGAATAGCATTTCATGCCTCCTTTCT     360
AN2like^Sly    TGTGATAGTGGCGTACGAACTCACAACTCTAGCATGAATAGCATTTCATGTCTCCTTTCT     360
               ************** * ***  * ****** ******************* ******* *

AN2like^WT     TATTACCGAGTCATAAATCAATTTCGTTAGGAGTTTACAAATTAATATACACATATATTT     419
AN2like^Aft    TATTACTGAGTCGTAAATTAATTTTGGTAGGAGTTTACAAGTTAATATATATATATATTT     417
AN2like^Abg    TATTACTGAGTCGTCAATCATTTTCGTTAGGAGTTTACAAGTTAATATAGACATATATTT     420
AN2like^Sly    TATTACTGAGTCGTCAATCATTTTCGTTAGGAGTTTACAAGTTAATATAGACATATATTT     420
               ****** ***** * *** * *** * ************* ******** * ********

AN2like^WT     AGTTAAATTTTTTTAGTTCATATATAACATCTACCAAAAAAATTACTGGATTCGTTCAAT     479
AN2like^Aft    GATTAA-TTTTTTTAGTTTATATACAATATCTAT-GAAAAAATTACTAGGTTCGTTCAAC     475
AN2like^Abg    ACTTAA--TTTTTTAGTCCATATACAATATCTAC-GAAAAAGTTACTTGATCTGTTCAAT     477
AN2like^Sly    GCTTAA--TTTTTTAGTCCATATACAATATCTAC-GAAAAAATTACTGAATCCGTTCAAC     477
                 ****  *********  ***** ** *****   ***** *****   *  ****** 

AN2like^WT     CCACAAATCCCCACTTACTATTATTTCATGTGAATATATGCAGGTCTAAATAGATGTCGA     539
AN2like^Aft    CCACAAATCCCCACTTACTATTATTTCACGTGATTATATGCAGGTCTAAATAGATGTCGA     535
AN2like^Abg    CCACAAATCCC-ACTTACTA---TTTCATACGAATATATGCAGGTCTAAATCGATGTCGA     533
AN2like^Sly    CCACAAATCCCCACTTACCA---TTTCATACAAATATATGCAGGTCTAAATAGATGTCGA     534
               *********** ****** *   *****    * ***************** ********

AN2like^WT     AAGAGTTGTAGACTGAGGTGGTTGAATTATCTAAGGCCACATATCAAGAGAGGTGACTTT     599
AN2like^Aft    AAGAGTTGTAGACTGAGGTGGTTGAATTATCTAAGGCCACATATCAAGAGAGGTGACTTT     595
AN2like^Abg    AAGAGTTGTAGACTGAGGTGGTTGAATTATCTAAGGCCACATATCAAGAGAGGTGACTTT     593
AN2like^Sly    AAGAGTTGTAGACTGAGGTGGTTGAATTATCTAAGGCCACATATCAAGAGAGGTGACTTT     594
               ************************************************************

AN2like^WT     GCTCTGGATGAAATAGATCTCATTTTGAGACTTCACAAGCTTCTAGGCAATAGGCAAATC     659
AN2like^Aft    GCTATGGATGAAATAGATCTCATTTTGAGACTTCACAAGCTTCTAGGCAATAGGCAAGTC     655
AN2like^Abg    GCTCCGGATGAAATAGATCTCATTTTAAGACTTCACAAGCTTCTAGGCAATAGGCAAGTC     653
AN2like^Sly    GCTCCGGATGAAATAGATCTCATTTTAAGACTTCACAAGCTTCTAGGCAATAGGCAAGTC     654
               ***  ********************* ****************************** **

AN2like^WT     AAAAATTTCGTTAAAAAATATTTAAAAATTATTGTACATATATATATTCACGAAAAGTAA     719
AN2like^Aft    AGAAATTTAGTTAAAAGAAATTCAAAAATTATTGTACATATATAT--TCACGAAAAGAAA     713
AN2like^Abg    AAAAAATTCGTTAAAAAGTATTCAAAAATTATTATACATATATAT--TTAGGAAAAGTAA     711
AN2like^Sly    AAAAATTTCGTTAAAAAGTATTCAAAAATTATTATACATATATAT--TTAGGAAAAGTAA     712
               * *** ** *******   *** ********** ***********  * * ****** **

AN2like^WT     TTTTTGACATATAAATTTACGTACATACTAGTCTCTCGAATATATTATAGTAAGTTATCT     779
AN2like^Aft    CTTTTGACATACAAATTTGTGTACATACTAGTCTTCCGTATATATTATAGTAAGTTGTCT     773
AN2like^Abg    TTTTTGACATATAAATCTGAGTACATACTTCTCTCTCAAATATACTATAGTAAGT-GTCT     770
AN2like^Sly    TTTTTGACATATAAATCTGTGTACATACTTCTCTCTCGAATATACTATAGTAAGT-GTCT     771
                ********** **** *  *********  ***  *  ***** **********  ***
```

```
AN2likeWT     TTGATGCCATATTTTTATAT-TTTTTTGGTTTAGATGGTCACTTATTGCTGGGAGACTTC    838
AN2likeAft    TTGATGCCATATTTTTATTT-TCTTTTGGTTTAGATGGTCACTTATTGCTGGGAGACTTC    832
AN2likeAbg    TTGATGCACTATTTTATTTTATTTTTTGGTTTAGGTGGTCACTTATTGCTGGGAGACTTC    830
AN2likeSly    TTGATGCAATATTTTATTTTATTTTTTGGTTTAGGTGGTCACTTATTGCTGGGAGACTTC    831
              *******  ******  * * * *********** *************************

AN2likeWT     CTGGAAGAACAGCAAACGATGTGAAAAACTATTGGAACACACACCTACACAAGAAGTTAT    898
AN2likeAft    CGGGAAGAACAGCAAACGATGTGAAAAACTATTGGAACACACACCTACACAAGAAGTTAT    892
AN2likeAbg    CAGGAAGAACAGCAAACGATGTGAAGAACTATTGGAACACACACCTACACAAGAAGTTAT    890
AN2likeSly    CAGGAAGAACAGCAAACGATGTGAAAAACTATTGGAACACACACCTACACAAGAAGTTAA    891
              * *********************** ********************************* 

AN2likeWT     TAATAACTCCTCCTCAGATACAAGAGAATAAGTACAATAATACCCTCAAGATTATCACTG    958
AN2likeAft    TAATAACTCCTC---AGATACAAGAGAATAAGTACAATAAAACCCTCAAGATTATCACTG    949
AN2likeAbg    TAATAACTCCTC---AGATACAAGAGAATAAGTACAATAATACCCTCAAGATTATCACTG    947
AN2likeSly    TAACTCCCCCTC---TGATACAAGAGAATAAGTACAATAATACCCTCAAGATTATCACTG    948
              ***   * ****    ************************ *******************

AN2likeWT     AAAGCACTATACTACGACCACGACCAAGACCAGGACCTCAACCTCGAACCTTCTCAAGTG    1018
AN2likeAft    AAAGCACTATACTACGACCACGACCAAGACC------TCGACCTCGAACATTCTCAAGTG    1003
AN2likeAbg    AAAGCACTATACTACGACCACGACC------------------TCGAACCTTTTCAAGTG     989
AN2likeSly    AAAGCACTATACTACGACCACGACCACGACCAGGACCTCGACCTCGAACCTTTTCAAGTG    1008
              *************************                  ****** ** *******

AN2likeWT     AAAATAATATTTCTTGGTGCACTAACAATAGTATGATCACAAACACATTAGACAAAGATG    1078
AN2likeAft    AAAATAATATTTCTTGGTGCACTAACAATAGTATGATCACAAACACATTAGACAAAGATG    1063
AN2likeAbg    AAAATAATATTTCTTGGTGCACTAACAATAGTATGATCACAAACACATTATACAAAGATG    1049
AN2likeSly    AAAATAATATTTCTTGGTGCACTAACAATAGTATGATCACAAACACATTAGACAAAGATG    1068
              ************************************************** *********

AN2likeWT     ACGAACAACACAACAAAGAATCGCAGTAAATATTTGTGAGAAGCCAACAAAAAAAACAC    1138
AN2likeAft    ACGAACAACGCAACAAAGAATCGCAGTAAATATTTGTGAGAAGCCAACAAGAGAAACAC    1123
AN2likeAbg    ACGAACAACGTAACAAAGAATCGTAGTAAATATTTGTGAGAAGCCAACAAGAGAAACTC    1109
AN2likeSly    ACGAACAACGTAACAAAGAATCGTAGTAAATATTTGTGAGAAGCCAACAAGAGAAACTC    1128
              *********  ************ *************************** * **** *

AN2likeWT     CGTCATCGTCTATAGACGATGATGGAGTTCAATGGTGGACAAATTTACTGGAAAATTGGA    1198
AN2likeAft    CGTCATCGTCTATAGACGATGATGGAGTTAAATGGTGGACAAATTTACTGGAAAATTGGA    1183
AN2likeAbg    CGTCATCGTCTATAGACGATGACGGAGTTAAATGGTGTACAAATTTACTGGAAAATTGGA    1169
AN2likeSly    CGTCATCGTATATAGACGATGACGGAGTTAAATGGTGGACAAATTTACAGGAAAATTGGA    1188
              ********* ************ ****** ******* ********** ***********

AN2likeWT     AAGAATTTGAGGAAGAAGCAACAGCAGTATTGAACTTTGAGGAAGAAAATAAG---TTGT    1255
AN2likeAft    AAGAATTTGAGGAAGAAGCAACAGCAGTATTGAACTTTGAGGAAGAAAATAAG---TTGT    1240
AN2likeAbg    AAGAATTTGAGGAAGCAGCAGCAGCAGTATTGAGCTTTGAGGAAGAAAATAAT---TTGT    1226
AN2likeSly    AAGAATTTGAGGAAGAAGCAGCAGCAGTATTGAGCTTTGAGGAAGAAAATAATAAGTTGT    1248
              *************** **** ************ ******************    ****

AN2likeWT     TGCCAAATTTGTTGTATGAGGAACATAATTCAACAACCATGCAACATGGAGAAAATGATG    1315
AN2likeAft    TACCAAATTTGTTGTGTGAGGAACATAATTCAACAACCATGCAACATGGAGAAAATGATG    1300
AN2likeAbg    TACCAAATTTGTTGAATGAGGGAAATAATTCAACGACCATGCAACATGGAGAAAATGATG    1286
AN2likeSly    TACCAAATTTGTTGTATGAGGGAAATAATTCAACGACCATGCAACATGGAGAAAATGATG    1308
              * ************  ***** * ********** *************************

AN2likeWT     ACTTTTCAGTTGATATTGACCTATGGAATCTATTTAATTAG        1356
AN2likeAft    ACTTTTCAGTTGATATTGACCTATGGAATCTATTTAATTAG        1341
AN2likeAbg    ACTTTTCAGTTGATATTGACCTATGGAATCTATTTAATTAG        1327
AN2likeSly    ACTTTTCAGTTGATATTGACCTATGGAATCTATTTAATTAG        1349
              *****************************************
```

Fig. S8. ClustalW alignment of the gene *AN2like* sequenced in *Aubergine* (Abg), *S. lycopersicoides* (Sly), wild type (WT), and *Anthocyanin fruit* (Aft) plants. Exons are highlighted in grey. The symbol * indicates perfect alignment. In red and green are highlighted, respectively, the nucleotides responsible for the alternative splicing or the canonical splicing of the pre-mRNA of the gene [20].

a

>*MYB113*[Abg]

ATGAATACTCCTATGTGTACATCATTAGGAGTAATTAGGAAAGGTTCATGGACTGAACAAGAAGATTTTCACTTGAGAAAATGCATTCAAAAATATGGTG
AAGGAAAGTGGAATCTTGTTCCTTCTAGAGCTGGTAATTAAACGTTATAATACAACTACTATTTTCCGATTCATCGTTCTTTCTTTCTTAAATTTCATGT
TAATACATGCAGGTTTAAATAGATGTCGGAAAAGCTGTAGACTGAGATGGCTAAATTATCTAAGACCACATATCAAAAGAGGTGACTTTGAACCAGATGA
AGTGGATCTCATTTTGAGGCTTCATAAGCTCTTAGGCAACAGGCAATTAATTAGTACTTTATAATATTTAATACTTTAATTTCATATATATTAATTACAC
TCAATTACGTAAATTATATTACTATGTATTACTGTCTATGTCCCTATTTAGTTGTCCACTTTAAGAAATGATACACGTATTAAAATAGCAACAATTAACA
TAGTGAAGTTACAATTTTATCCTTATTAATTATGGTTTCAAAAAGTTTGAATTAAAACTTGAAAATTTTCAAGAAGTTTAAATAAGGGTATAATAGATTA
TTTTTTTTGTCTTTTCTTAATTTGTCAAAATGGACAAGTAAATAGGGACATGTAAAATAGGAAATATGGACAAATAAATAGGAACAAATGGAGTGTATAA
CATGTCCACTCATGTAGTCATGCGGTACTATATGTTTGTAAAATGTGTAATCATATCATCTAAAAATTACGTTATTTTGTGTAGATGGTCACTTGTTGCT
GGTAGACTTCCAGGAAGGACTTGTAAAATGTGTGTAATCATATCATCTAAAAATTACGTTATTTTGTGTAGGTGGTCACTTATTGCTGGTAGACTTCCAG
GAAGGACAGCAAACGATGTGAAAAATTACTGGAATACTCGTCTTCTAAGGAAGTTAAATACTAATATTACAAAGAATGAAATAATAAAACCTCAACCTCG
GACCTTATCATCAAATGCAAAGAATGTTTCTTGGTGCAACAACAAAAGTATGATCACAAACACATTAGACAAAGATGACAAACAACGGAACAAAGAAATC
GTAGTAAATATCTGTGAGAAGCCAACATCATCGTCTATAGACGATGACGGAGTTAAATGGTGGACAAGTTTACTGGAAAATTGTAATGAAATTGAGGATG
AAGAAGCAGCAGTATTGAGCTTTGAAGAAGAAAATAAGTTGTTACCAAATTTGTTGCATGAGGAAAATAATTCACCAATCATGCTACAAGGAGAAAGTGA
TGGTTGGGATATTGACTTATTATGGAATCTACTTAATTAA

>*MYB113*[Sly]

ATGAATACTCCTATGTGTACATCATTGGGAGTAATTAGGAAAGGTTCATGGACTGAACAAGAAGATTTTCACTTGAGAAAATGCATTCAAAAATATGGTG
AAGGAAAGTGGAATCTTGTTCCTTCTAGAGCTGGTAATTAACGTTATAATACAACTACTATTTTCCGATTCATCGTTCTTTCTTTCTTAAATTTCATGTT
AATACATGCAGGTCTAAATAGATGTCGAAAAAGCTGTAGACTGAGATGGCTAAATTATCTAAGACCACATATCAAAAGAGGTGACTTTGAACCAGATGAA
GTGGATCTCATTTTGAGGCTTCATAAGCTCTTAGGCAACAGGCAATTAATTAGTACTTTATAATATTTAATACTTTAATTTCATATATATTAATTACACT
CAATTACGTAAATTATATTACTATGTATTACTCCCTATGTTCCTATTTAGTTGTCCACTTTAAGAAATGACACACGTATTAAAATAGCAATAATTAACAT
AGTGAAGTTACAATTTTATCCTTATTAATTATGGTTTCAAAAAGGATGAATTAAAACTTAAAAATTTTCAAGAAGTTTAATTAGGGTATAATAGAAAAAA
AAATTGTCTTTTTTTTATTTGTTAAAATGAACAAGTAAATAGAGACAGCTAAAAGAGGAAACATGGACAAGTAAATAGGGACAGATGGAGTATATAACAT
GTCCACTCATGCGGTACTATTATATGTTGTGAAATGTGTAATCATATCATCTAAAAATTACGTTATTTTGTGTAGATGGTCACTTATTGCTGATAGACTT
CCAGGAAGGACTTGTAAAATGTGTAATCATATCATCTAAAAATTACGTTATTTTGTGTAGGTGGTCACTTATTGCTGGTAGACTTCCAGGAAGGACAGCA
AACGATGTGAAAAATTACTGGAATACTCGTCTTCTAAGGAAGTTAAATACTAATATTACAAAGAATGAAATAATAAAACCTCAACCTCGGACCTTATCAT
CAAATGCAAAGAATGTTTCTTGGTGCAACAACAAAAGTATGATCACAAACACATTAGACAAAGATGACGAACAACGCAACAAAGAAATCGTAGTAAGTAT
TTGTGAGAAGCCAACATCATCGTCTATAGACGATGACGGAGTTGAATGGTGGACAAGTTTACTGGAAAATTGTAATGAAATTGAGGATGAAGAAGCAGCA
GTATTGAGATTTGAAGAAGAAAATAAGTCGTTACCAAATTTGTTGCATGAGGAAAGTAATTCACCAATCATGCTACAAGGAGAAAGTGATGGTTGGGATA
TTGACTTATTATGGAATCTACTTAATTAA

b

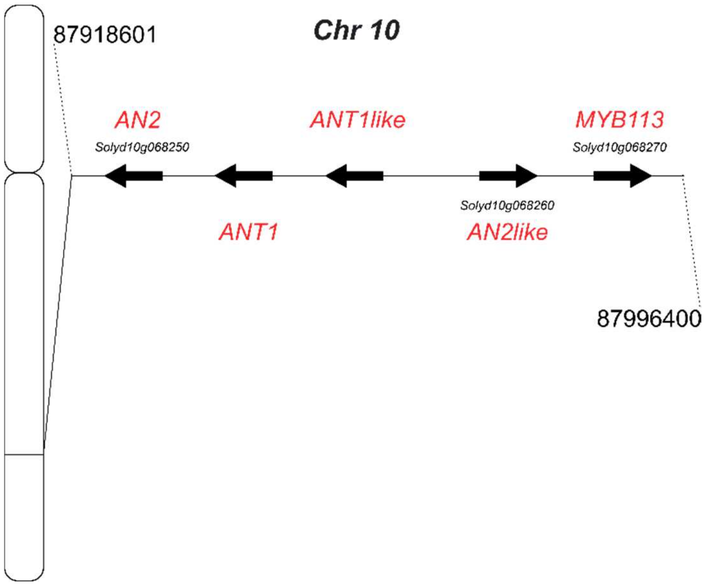

Fig. S9. Sequence analysis of the gene *MYB113* in *Aubergine* (*Abg*) and *S. lycopersicoides* (*Sly*). **a** Sequence of the gene *MYB113* in *Aubergine* (Abg) and *S. lycopersicoides* (Sly). Exons are highlighted in grey. **b** Position of the gene *MYB113* in the distal part of the long arm of chromosome 10 in *S. lycopersicoides* genome compared to the positions of the other four *R2R3 MYB* genes.

```
ANT1_S.lycopersicum          MNsT-smsSLGV-RKGSWTdEEDFLLRKCIdKYGEGKWHLVPIRAGLNRCRKSCRLRWLN
ANT1_Abg                     MNsT-smsSLGV-RKGSWTdEEDFLLRKCInKYGEGKWHLVPMRAGLNRCRKSCRLRWLN
ANT1like_S.lycopersicum      MNsT-smsSLGV-RKGSWTEqEDlLLRKCInKYGEGKWHLVPIRAGLNRCRKSCRLRWLN
ANT1like_Abg                 MNsT-smsSLGi-RKGSWTEEEDFLLRKCInKYGEGKWHLVPIRAGLNRCRKSCRLRWLN
PHZ_P.hybrida                MNTT-IpKSsGlVRKGaWTEEEDvLLRKCIEKfGEGKWHqVPVRAGLNRCRKSCRLRWLN
MYB113like_N.attenuata_      MNTTtISKSsGV-RKGaWTEEEDlLLRrCvEmYGEGKWHkVPIRAGLNRCRKSCRLRWLN
Anthocyanin2_C.chinense      MNTaIIAKSsGV-RKGaWTEEEDFLLRKCIQnYGEGKWHLVPIRAGLNRCRKSCRLRWLN
MYB113like_C.annuum          MNTaIIAKSsGV-RKGaWTEEEDFLLRKCIQnYGEGKWHLVPIRAGLNRCRKSCRLRWLN
AN2_S.lycopersicum           MNTp-McaSLGV-RKGSWTEqEDsLLRdCIQKYGEGKWHLVPaRAGLNRCRKSCRLRWLN
AN2_Abg                      MNTp-McaSLGV-RKGSWTEqEDFLLRnCIQKYGEGKWHLVPaRAGLNRCRKSCRLRWLN
MYB113like_S.tuberosum       MNTp-MctSLGV-kKGSWTEEEDFhLRKCIQKYGEGKWHLVPsRAGLNRCRKSCRLRWLN
MYB113_Abg                   MNTp-MctSLGVIRKGSWTEqEDFhLRKCIQKYGEGKWnLVPsRAGiNRCRKSCRLRWLN
MYB113_S.tuberosum           MN---IAKSLGV-RKGSWTkEEDiLLRKCIdKYGEGKWHLVPsRAGLNRCRKSCRLRWLN
AN2like_S.lycopersicum       MN---IAKtLGV-RKGSWTEdEDiLLRKCIdKYGEGKWHLVPfRAGLNRCRKSCRLRWLN
AN2like_Abg                  MN---IAKtLGV-RKGSWTEEEDtLLRKCInKYGEGKWHLVPsRAGLNRCRKSCRLRWLN
                                                      R2

ANT1_S.lycopersicum          YLRPHIKRGDFeqDEVDLILRLHKLLGNRWSLIAGRLPGRTANDVKNYWNTnLLRKLN--
ANT1_Abg                     YLRPHIKRGDFeqDEVDLILRLHKLLGNRWSLIAGRLPGRTANDiKNYWNTnLLRKLN--
ANT1like_S.lycopersicum      YLRPHIKRGDFAsDEiDLILRLHKLLGNRWSLIAGRLPGRTANDVKNYWNTnLLRKvN--
ANT1like_Abg                 YLRPHIKRGDFeqDEVDLILRLHKLLGNRWSLIAGRLPGRTANDVKNYWNTnLLRKLN--
PHZ_P.hybrida                YLRPHIKRGDFSeDEVDLIfRLHKLLGNRWSLIAGRLPGRTANDVKNYWNTHLqRKLi--
MYB113like_N.attenuata_      YLRPHIKRGDFSsDEiDLILRLHKLLGNRWSLIAGRLPGRTANDVKNYWNTHLqRKLi--
Anthocyanin2_C.chinense      YLRPHIKRGDFgwDEiDLILRLHKLLGNRWSLIAGRLPGRTANDVKNYWNsHLqkKLi--
MYB113like_C.annuum          YLRPHIKRGDFgwDEiDLILRLHKLLGNRWSLIAGRLPGRTANDVKNYWNsHLqkKLi--
AN2_S.lycopersicum           YLRPHIKRGDFApDEVDLILRLHKLLGNRWSLIAGRLPGRTANDVKNYWNTHfhkKLSII
AN2_Abg                      YLRPHIKRGDFApDEVDLILRLHKLLGNRWSLIAGRLPGRTANDVKNYWNTHfhkKLNII
MYB113like_S.tuberosum       YLRPHIKRGDFepDEVDLILRLHKLLGNRWSLIAGRLPGRTANDVKNYWNTHLLRKLN--
MYB113_Abg                   YLRPHIKRGDFepDEVDLILRLHKLLGNRWSLIAGRLPGRTANDVKNYWNTrLLRKLN--
MYB113_S.tuberosum           YLRPHIKRGDFApDEiDLILRLHKLLGNRWSLIAGRLPGRTANDVKNYWNTHLhRKLi--
AN2like_S.lycopersicum       YLRPHIKRGDFAlDEiDLILRLHKLLGNRWSLIAGRLPGRTANDVKNYWNTHLhkKLl--
AN2like_Abg                  YLRPHIKRGDFApDEiDLILRLHKLLGNRWSLIAGRLPGRTANDVKNYWNTHLhkKLl--
                                                      R3

ANT1_S.lycopersicum          ------TTKi--vprEKinNKcgeIsTKiEIIkPQ------rRkyfSS---------tmK
ANT1_Abg                     ------TSKi--vprEKinNKcgeIsTKiEIIkPQ------PRkyfSStK----------
ANT1like_S.lycopersicum      ------iTKi--vprEKfKsKrgeIsTKiEIIkPQ------aRkFiSn---------teK
ANT1like_Abg                 ------TTKi--varEKiKsKrgeIsdKiEIIkPQ------PRkFiSn---------tkK
PHZ_P.hybrida                ------a--P--arQEirKcRalK-ITeNnIVRPr------PRTFSnSAqNIS-WC-sNK
MYB113like_N.attenuata_      ---------P--lqrqdrKcRvlK-ITeNtIVRPr------PRTFSS-AKNVS-lC-sNK
Anthocyanin2_C.chinense       ------Ta-P--hrQERkystalK-ITKknvlRPr------PRTFSSSAKNnisWCTNks
MYB113like_C.annuum          ------Ta-P--hrQEKkyNtalK-ITtknvlRPr------PRTFSSSAKNnisWCTNks
AN2_S.lycopersicum           APHLHPhSRPrshprlQiKhKsia-VTKNEIIRPQ------PRnFSnvkKNdSHWC-NNK
AN2_Abg                      APHLHPrpRP--hphlQiKhKsia-VTKNEIIkPQ------PRnFSnvnKNISHWCnNNK
MYB113like_S.tuberosum       ------Tn-----------------ITKNEIIkPQ------PRTlSSnAKNVS-WC-NNK
MYB113_Abg                   ------Tn-----------------ITKNEIIkPQ------PRTlSSnAKNVS-WC-NNK
MYB113_S.tuberosum           ------Tpph--eiQEnkyNntlK-ITeStIlRPrPR----PRTFSS-AnNIS-WC-tNn
AN2like_S.lycopersicum       ------iTpP--qiQEnkyNntlKIITeStIlRPrPRpgpqPRTFSS-enNIS-WC-tNn
AN2like_Abg                  ------iT-P--qiQEnkyNntlKIITeStIlRPr------PRTFSS-enNIS-WC-tNn

ANT1_S.lycopersicum          nvtnNnvilDeEehCKEI---IsEKqTp-----daSmD---nvdpWWiNLLENCNDdIEE
ANT1_Abg                     ---mNivifDeEehCKEI---IsEKqTp-----daSmD---nvdQWWTNLLENCNDdVEE
ANT1like_S.lycopersicum      nitnNivivDkEeeCKEI---IsEKqTr-----daSID---nGdEWWaNLLENCNDdVvE
ANT1like_Abg                 nitsNivieDkEeqCKEI---tsEKqTr-----daSID---nGdEWWeNLLENCNDdVEE
PHZ_P.hybrida                SitnsTiDtDg--snnEcIrindkKPma-----evSrD---DGVQWWTsLLaNCN-EndE
MYB113like_N.attenuata_      S-ITkTihKeDgsKen----NICEKPig-----daptD---hGIQWWTsLLdNCN-EIEE
Anthocyanin2_C.chinense      tvITNTLDKDe--RdKEIglNICqKlTsET---SStID---DGVhWWTsLLENCk-EIEE
MYB113like_C.annuum          tvITNTLDKDe--RdKEIglNICqKlTsET---SStID---DGVQWWTsLLENCk-EIEE
AN2_S.lycopersicum           SMITNTLDKDDk-RCnEIVVNICEKPigEn---tSSID---DGVEWWTNLLENCi-EIEE
AN2_Abg                      SMITNTLDKDDk-RCKEmVVNICEKPigEn---tSSID---DGVEWWTNLLvNCN-EIEE
MYB113like_S.tuberosum       SMITNTLDKDDEQRnKEIVVNICEKPTgETttlSSSIDD--DrVEWWTsLLENCN-EIEd
MYB113_Abg                   SMITNTLDKDDkQRnKEIVVNICEKPT------SSSIDD--DGVkWWTsLLENCN-EIEd
MYB113_S.tuberosum           SMITNTLDKDDEQRnKEIVVNICEKPTrETt--SSSIDDdgDGVkWWTdLLENwk-EfEE
AN2like_S.lycopersicum       SMITNTLDKDDEQhnKEIaVNICEKPTkkTP--SSSIDD--DGVQWWTNLLENwk-EfEE
AN2like_Abg                  SMITNTLyKDDEQRnKEIVVNICEKPTrETP--SSSIDD--DGVkWcTNLLENwk-EfEE
```

```
ANT1_S.lycopersicum          Dee----vViNyEkT---LtSLLHEEIsPPL-NiGeGNsMQQGQiSHenWGEFSLNLPPM
ANT1_Abg                     Dee----vViNyEkT---LtSLLHEEIsPPL-NgegnsiMQQGQtSHDsWGDFSLNLPPM
ANT1like_S.lycopersicum      EeeggggVtdygkT---ItSLLHEEItPPL--NGgGNiMQQeQS--DGW----------
ANT1like_Abg                 Dee----vViNyEkT---LtSLLHEEItPPL--NGgGNfMQQGQS--DGW----------
PHZ_P.hybrida                pAv----enmsydk----LPSLLHEEIsPPM-NgGisNcMQeGQS---GW----------
MYB113like_N.attenuata_      aAA---vgsiNlEEnNKLL-SLLHEEIsPPI--NGvsNcMQeGQS--gnW----------
Anthocyanin2_C.chinense      DvA----AVgiFEEkNKLvPSLLHdEINslt--------MQQGQS--DGW----------
MYB113like_C.annuum          DvA----AVgiFEEkNKLvPSLLHdEINslt--------MQQGQS--DGW----------
AN2_S.lycopersicum           Et-----AntNFgkT---ptmLLHEEIsPPL-vNGedNsMQQGpt--nnW----------
AN2_Abg                      EA-----vVtNFEkT---ptmLLnEEIsPPLiiNGeGNsMQQGQt-HDnW----------
MYB113like_S.tuberosum       EAA----vVlsFEEeNKLLPnLLHqEnNsPI--------MlQGeS---------------
MYB113_Abg                   EeA----AVlsFEEeNKLLPnLLHEEnNsPI--------MlQGeS---------------
MYB113_S.tuberosum           EAA----AVlsFEEeNKLLPnLLyEEhNstt--------MQhGen--DGW----------
AN2like_S.lycopersicum       EAt----AVlNFEEeNKLLPnLLyEEhNstt--------MQhGen---------------
AN2like_Abg                  aAA----AVlsFEEeNnLLPnLLnEgnNstt--------MQhGen---------------
```

```
ANT1_S.lycopersicum          QQGVQNDD---FSaeID-LWNLLd
ANT1_Abg                     QQGVQNDDwddFSaeID-LWNLLd
ANT1like_S.lycopersicum      ------DD---FfVDID-iWdLLN
ANT1like_Abg                 ------DD---FfVDID-iWdLLN
PHZ_P.hybrida                ------DD---FSVDIDhLWNLLN
MYB113like_N.attenuata_      ------DD---FSVDID-LWdLLN
Anthocyanin2_C.chinense      ------DD---FSaDID-LWNLLN
MYB113like_C.annuum          ------DD---FSaDID-LWNLLN
AN2_S.lycopersicum           ------DD---FStDID-LWNLLN
AN2_Abg                      ------DD---FStDID-LWNLLN
MYB113like_S.tuberosum       ------Dg---wgIDl--LWNLLN
MYB113_Abg                   -------Dg---wdIDl-LWNLLN
MYB113_S.tuberosum           ------DD---FSVDID-LWNLfN
AN2like_S.lycopersicum       ------DD---FSVDID-LWNLfN
AN2like_Abg                  ------DD---FSVDID-LWNLfN
```

Fig. S10. MUSCLE 3.8.31 alignment (www.phylogeny.fr) [54] of the sequences of *Solanaceae* R2R3 MYB proteins similar to MYB113 identified in *Aubergine* (*Abg*). Similar residues are colored as the most conserved one (according to BLOSUM62). Average BLOSUM62 score: Max: 3.0, Mid: 1.5, Low: 0.5. R2 and R3 MYB domains are underlined. In each alignment the [DE]Lx2[RK]x3Lx6Lx3R motif containing the bHLH-binding site [45] is boxed in blue, the aminoacidic signature [A/S/G]NDV typical of dicot R2R3 MYBs promoting anthocyanin [46] is boxed in green and the KPRPR[ST]F motif, conserved in Arabidopsis MYBs involved in anthocyanin synthesis [3], is boxed in red. GenBank and SolGenomics accession numbers: MYB113like_*S. tuberosum* (XP_006349525.1), MYB113_*S. tuberosum* (ALA13583.1), Anthocyanin2_*C. chinense* (QLC27705.1), MYB113like_*C. annuum* (NP_001311547.1), PHZ_*P. hybrida* (ADQ00389.1), MYB113like_*N. attenuata* (XP_019240653.1), AN2_*S. lycopersicum* (*Solyc10g086250*), AN2_*Abg* (OP094093), ANT1_*S. lycopersicum* (*Solyc10g086260*), ANT1_*Abg* (OP094102), ANT1like_*S. lycopersicum* (*Solyc10g086270*), ANT1like_*Abg* (OP094090), AN2like_*S. lycopersicum* (*Solyc10g086290*), AN2like_*Abg* (OP094099), MYB113_*Abg* (OP094101).

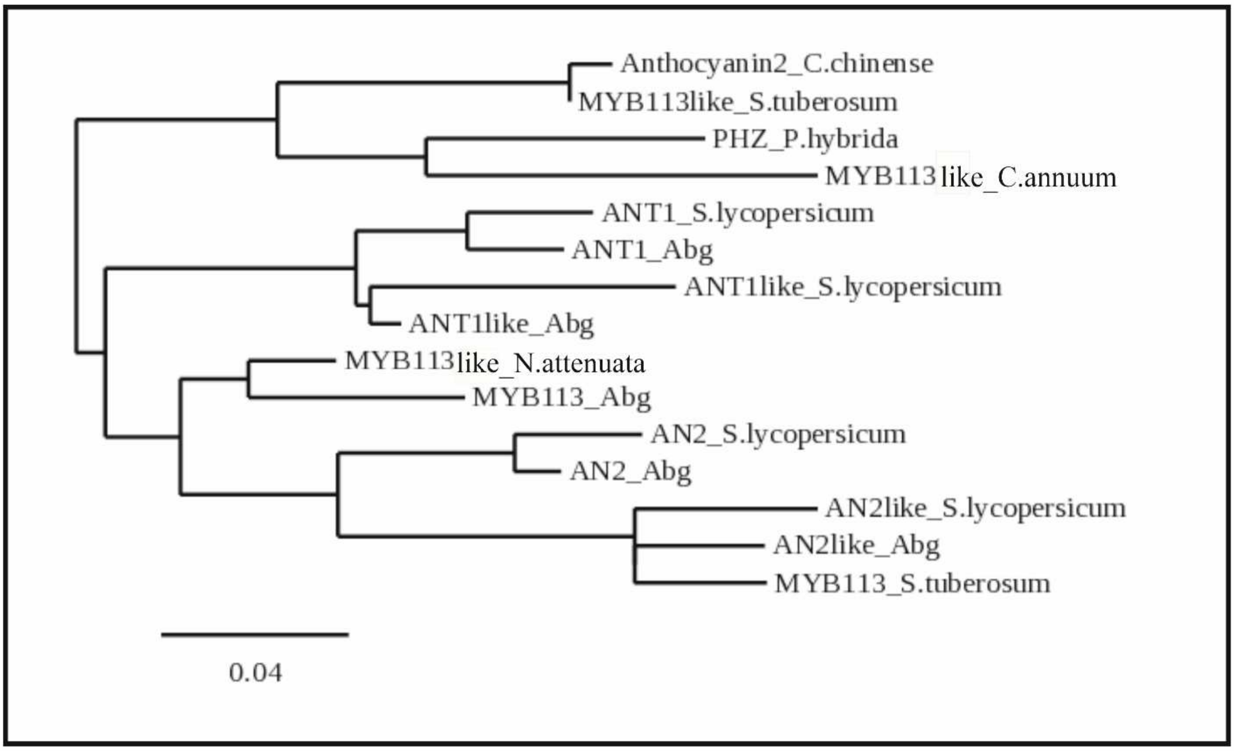

Fig. S11. Phylogenetic tree of MYB113^{Abg} and other *Solanaceae* R2R3 MYB proteins. The analysis was performed on the phylogeny.fr platform [54]. GenBank and SolGenomics accession numbers: MYB113like_*S. tuberosum* (XP_006349525.1), MYB113_*S. tuberosum* (ALA13583.1), Anthocyanin2_*C. chinense* (QLC27705.1), MYB113like_*C. annuum* (NP_001311547.1), PHZ_*P. hybrida* (ADQ00389.1), MYB113like_*N. attenuata* (XP_019240653.1), AN2_*S. lycopersicum* (*Solyc10g086250*), AN2_*Abg* (OP094093), ANT1_*S. lycopersicum* (*Solyc10g086260*), ANT1_*Abg* (OP094102), ANT1like_*S. lycopersicum* (*Solyc10g086270*), ANT1like_*Abg* (OP094090), AN2like_*S. lycopersicum* (*Solyc10g086290*), AN2like_*Abg* (OP094099), MYB113_*Abg* (OP094101).

a

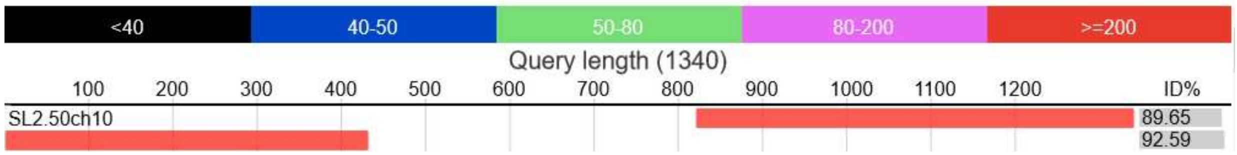


```
>SL2.50ch10
Length=65527505

 Score = 667 bits (361),  Expect = 0.0
 Identities = 485/541 (90%), Gaps = 24/541 (4%)
 Strand=Plus/Plus

Query  821       TTGTAAAATGTGTGTAATCATATCATCTAAAAATTACGTTATTTTGTGTAGGTGGTCACT  880
                 ||||||||  ||| ||| ||||||||| ||| |||| ||||||| |||||||||| ||||
Sbjct  65176901  TTGTAAAA--TGTATAACCATATCATCGAAAGATTATGTTATTTCGTGTAGGTGGACACT  65176958

Query  881       TATTGCTGGTAGACTTCCAGGAAGGACAGCAAACGATGTGAAAAATTACTGGAATACTCG  940
                 |||||||||||||||||| |||||||||||||||||| | ||||| ||||||||||||||
Sbjct  65176959  TATTGCTGGTAGACTTCCGGGAAGGACAGCAAACGATATAAAAAACTACTGGAATACTCG  65177018

Query  941       TCTTCTAAGGAAGTTAAATACTAATATTACAAAGAATGAAATAATAAAACCTCAACCTCG  1000
                 |||||||| ||||||||||||||||||||| || |||||||||| |||||||||||||| 
Sbjct  65177019  TCTTCTAA-GAAGTTAAATACTAATATTACGAATAATGAAATAACAAAACCTCAACCTCA  65177077

Query  1001      GACCTTATCATCAAATGCAAAGAATGTTTCTTGGTGCAACAACAAAAGTATGATCACAAA  1060
                 ||| |||||||||||||||||||||||||||| |||||||||||||||||||||||||||
Sbjct  65177078  GACGTTATCATCAAATGCAAAGAATGTTTCTTTGTGCAACAACAAAAGTATGATCACAAA  65177137

Query  1061      CACATTAGACAAAGATGACAAACAACGGAACAAAGAAATCGTAGTAAATATCTGTGAGAA  1120
                 ||||||||| ||||||||  ||||||| |||||||||||||||||||||| || |||||
Sbjct  65177138  CACATTAGATAAAGATGATGAACAACGCAACAAAGAAATCGTAGTAAATATTTGGGAGAA  65177197

Query  1121      GCCAAC---A------------T---CATCGTCTATAGACGATGACGGAGTTAAATGGTG  1162
                 ||||||   |            |   |||||||||||||||||||| ||||| |||||||
Sbjct  65177198  GCCAACGGGAGAAACAACAACATTGCCATCGTCTATAGACGATGACAGAGTTGAATGGTG  65177257

Query  1163      GACAAGTTTACTGGAAAATTGTAATGAAATTGAGGATGAAGAAGCAGCAGTATTGAGCTT  1222
                 |||||| ||||||||||||||||| |||||||||||||||||||||| ||||||||||||
Sbjct  65177258  GACAAGCTTACTGGAAAATTGTAAAGAAATTGAGGATGAAGAAGCAGTAGTATTGAGCTT  65177317

Query  1223      TGAAGAAGAAAATAAGTTGTTACCAAATTTGTTGCATGAGGAA---AATAATTCACCAAT  1279
                 ||||||||||| |||||| ||| ||||||||||||||||||||   ||||||||||||||
Sbjct  65177318  TGAAGAAGAAACTAAGTTATTATCAAATTTGTTGCATGAGGAAAATAATAATTCACCAAT  65177377

Query  1280      CATGCTACAAGGAGAAAGTGATGGTTGGGATATTGACTTATTATGGAATCTACTTAATTA  1339
                 ||||||||||||||||||| ||||||||||||||||||||||||||||||||||||||||
Sbjct  65177378  CATGCTACAAGGAGAAAGTCATGGTTGGGATATTGACTTATTATGGAATCTACTTAATTA  65177437

Query  1340      A  1340
                 |
Sbjct  65177438  A  65177438


Score = 617 bits (334),  Expect = 1e-174
 Identities = 400/432 (93%), Gaps = 4/432 (1%)
 Strand=Plus/Plus

Query  1         ATGAATACTCCTATGTGTACATCATTAGGAGTAATTAGGAAAGGTTCATGGACTGAACAA  60
                 ||||||||||||||||| |||||||| || | | ||||||||||||| ||||||||||||
Sbjct  65176441  ATGAATACTCCTATGTGCACATCATT-GG-G-AGTTAGGAAAGGTTCGTGGACTGAACAA  65176497

Query  61        GAAGATTTTCACTTGAGAAAATGCATTCAAAAATATGGTGAAGGAAAGTGGAATCTTGTT  120
                 ||||||||||||||||||||||||||| |||| |||||||||||||||||||||||||||
Sbjct  65176498  GAAGATTTTCACTTGAGAAAATGCATTAAAAAGTATGGTGAAGGAAAGTGGAATCTTGTT  65176557

Query  121       CCTTCTAGAGCTGGTAATTAAACGTTATAATACAACTACTATTTTCCGATTCATCGTTCT  180
                 ||||||||||||||||||||||||||||||||  |||| |||||||||||||||| ||||
Sbjct  65176558  CCTTCTAGAGCTGGTAATTAAACGTTATAATAATACTATTATTTTCCGATTCATCATTCT  65176617
```

```
Query  181       TTCTTTCTTAAATTTCATGTTAATACATGCAGGTTTAAATAGATGTCGGAAAAGCTGTAG  240
                 |||||||||||||||| ||||||||||||  ||| |||||||||||||||||||||||||
Sbjct  65176618  TTCTTTCTTAAATTTCGTGTTAATACATGTGGGTCTAAATAGATGTCGGAAAAGCTGTAG  65176677

Query  241       ACTGAGATGGCTAAATTATCTAAGACCACATATCAAA-AGAGGTGACTTTGAACCAGATG  299
                 |||||||||||||||||||||| | |||||||||||| ||||||| ||||||||  ||||
Sbjct  65176678  ACTGAGATGGCTAAATTATCTACGGCCACATATCAAAGAGAGGTGTCTTTGAACGGGATG  65176737

Query  300       AAGTGGATCTCATTTTGAGGCTTCATAAGCTCTTAGGCAACAGGCAATTAATTAGTACTT  359
                 |||||||| | ||||||| ||||||| |||||||||||||||||||||||||||||||||
Sbjct  65176738  AAGTGGATTTAATTTTGACGCTTCATTAGCTCTTAGGCAACAGGCAATTAATTAGTACTT  65176797

Query  360       TATAATATTTAATACTTTAATTTCATATATATTAATTACACTCAATTACGTAAATTATAT  419
                 |||||| |||||||||||||| |||||||||||| |||||||| ||||| ||||||||| 
Sbjct  65176798  TATAATGTTTAATACTTTAATCTCATATATATTAGTTACACTCGATTACATAAATTATAC  65176857

Query  420       TACTATGTATTA  431
                 ||||||||||||
Sbjct  65176858  TACTATGTATTA  65176869
```

b

>*MYB113*[WT] genomic sequence

ATGAATACTCCTATGTGCACATCATTGGGAGTTAGGAAAGGTTCGTGGACTGAACAAGAAGATTTTCACTTGAGAAAATGCATTAAAAAGTATGGTGAAG
GAAAGTGGAATCTTGTTCCTTCTAGAGCTGGTAATTAAACGTTATAATAATACTATTATTTTCCGATTCATCATTCTTTCTTTCTTAAATTTCGTGTTAA
TACATGTGGGTCTAAATAGATGTCGGAAAAGCTGTAGACTGAGATGGCTAAATTATCTACGCCACATATCAAAGAGAGGTGTCTTTGAACGGGATGAAGT
GGATTTAATTTTGACGCTTCATTAGCTCTTAGGCAACAGGCAATTAATTAGTACTTTATAATGTTTAATACTTTAATCTCATATATATTAGTTACACTCG
ATTACATAAATTATACTACTATGTATTATATAACATGTCCGCTCATGCAATACTATATGTTGTAAAATGTATAACCATATCATCGAAAGATTATGTTATT
TCGTGTAGGTGGACACTTATTGCTGGTAGACTTCCGGGAAGGACAGCAAACGATATAAAAAACTACTGGAATACTCGTCTTCTAAGAAGTTAAATACTAA
TATTACGAATAATGAAATAACAAAACCTCAACCTCAGACGTTATCATCAAATGCAAAGAATGTTTCTTTGTGCAACAACAAAAGTATGATCACAAACACA
TTAGATAAAGATGATGAACAACGCAACAAAGAAATCGTAGTAAATATTTGGGAGAAGCCAACGGGAGAAACAACAACATTGCCATCGTCTATAGACGATG
ACAGAGTTGAATGGTGGACAAGCTTACTGGAAAATTGTAAAGAAATTGAGGATGAAGAAGCAGTAGTATTGAGCTTTGAAGAAGAAACTAAGTTATTATC
AAATTTGTTGCATGAGGAAAATAATAATTCACCAATCATGCTACAAGGAGAAAGTCATGGTTGGGATATTGACTTATTATGGAATCTACTTAATTAA

>putative *MYB113*[WT] transcript (**FORWARD STRAND**)

ATGAATACTCCTATGTGCACATCATTGGGAGTTAGGAAAGGTTCGTGGACTGAACAAGAAGATTTTCACTTGAGAAAATGCATTAAAAAGTATGGTGAAG
GAAAGTGGAATCTTGTTCCTTCTAGAGCTGGTATAAATAGATGTCGGAAAAGCTGTAGACTGAGATGGCTAAATTATCTACGCCACATATCAAAGAGAGG
TGTCTTTGAACGGGATGAAGTGGATTTAATTTTGACGCTTCATTAGCTCTTAGGCAACAGGTGGACACTTATTGCTGGTAGACTTCCGGGAAGGACAGCA
AACGATATAAAAAACTACTGGAATACTCGTCTTCTAAGAAGTTAAATACTAATATTACGAATAATGAAATAACAAAACCTCAACCTCAGACGTTATCATC
AAATGCAAAGAATGTTTCTTTGTGCAACAACAAAAGTATGATCACAAACACATTAGATAAAGATGATGAACAACGCAACAAAGAAATCGTAGTAAATATT
TGGGAGAAGCCAACGGGAGAAACAACAACATTGCCATCGTCTATAGACGATGACAGAGTTGAATGGTGGACAAGCTTACTGGAAAATTGTAAAGAAATTG
AGGATGAAGAAGCAGTAGTATTGAGCTTTGAAGAAGAAACTAAGTTATTATCAAATTTGTTGCATGAGGAAAATAATAATTCACCAATCATGCTACAAGG
AGAAAGTCATGGTTGGGATATTGACTTATTATGGAATCTACTTAATTAA

c

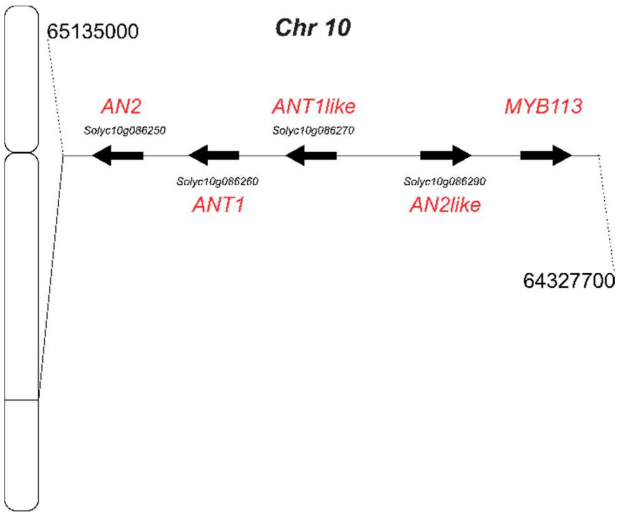

d

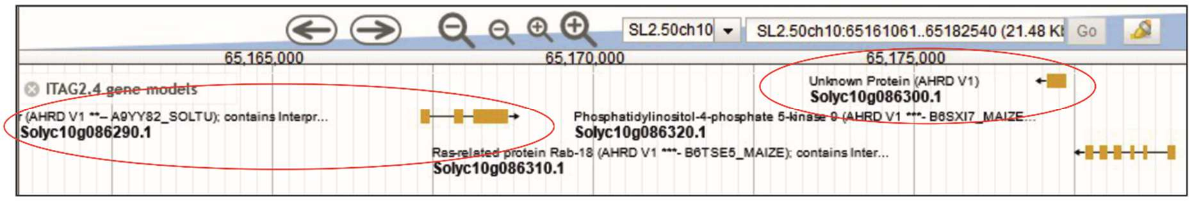

e

>Solyc10g086300.1.1 Unknown Protein (**REVERSE STRAND**)
ATGATTGGTGAATTATTATTTTCCTCATGCAACAAATTTGATAATAACTTAGTTTCTTCTTCAAAGCTCAATACTACTGCTTCTTCATCCTCAATTTCTT
TACAATTTTCCAGTAAGCTTGTCCACCATTCAACTCTGTCATCGTCTATAGACGATGGCAATGTTGTTGTTTCTCCCGTTGGCTTCTCCCAAATATTTAC
TACGATTTCTTTGTTGCGTTGTTCATCATCTTTATCTAATGTGTTTGTGATCATACTTTTGTTGTTGCACAAAGAAACATTCTTTGCATTTGATGATAAC
GTCTGA

Fig. S12. a Blast sequence analysis of the gene *MYB113* identified in *Aubergine* used as a query with the genome of *S. lycopersicum*. Only the two most significant alignments are shown. **b** Sequence of the putative *MYB113* gene of tomato and of its transcript. The initial ATG and the premature stop codon are in red. **c** Position of the gene *MYB113* in the distal part of the long arm of chromosome 10 in *S. lycopersicon* genome compared to the positions of the other four *R2R3 MYB* genes. **d** JBrowse showing the position of the sequence *Solyc10g086300.1* in tomato chromosome 10 with respect to the position of *AN2like* (*Solyc10g086290.1*). The tools used in the analyses shown in a and d are from the Sol Genomics Network website. **e** Sequence of the *Solyc10g086300.1.1* transcript annotated in the Sol Genomics Network database.

```
MYB113^WT       ATGAATACTCCTATGTGCACATCA---TTGGGAGTTAGGAAAGGTTCGTGGACTGAACAA 57
MYB113^Sly      ATGAATACTCCTATGTGTACATCATTGGGAGTAATTAGGAAAGGTTCATGGACTGAACAA 60
MYB113^Abg      ATGAATACTCCTATGTGTACATCATTAGGAGTAATTAGGAAAGGTTCATGGACTGAACAA 60
                ***************** ******      * * ************* ************

MYB113^WT       GAAGATTTTCACTTGAGAAAATGCATTAAAAAGTATGGTGAAGGAAAGTGGAATCTTGTT 117
MYB113^Sly      GAAGATTTTCACTTGAGAAAATGCATTCAAAAATATGGTGAAGGAAAGTGGAATCTTGTT 120
MYB113^Abg      GAAGATTTTCACTTGAGAAAATGCATTCAAAAATATGGTGAAGGAAAGTGGAATCTTGTT 120
                ************************** **** ****************************

MYB113^WT       CCTTCTAGAGCTGGTAATTAAACGTTATAATAATACTATTATTTTCCGATTCATCATTCT 177
MYB113^Sly      CCTTCTAGAGCTGGTAATTA-ACGTTATAATACAACTACTATTTTCCGATTCATCGTTCT 179
MYB113^Abg      CCTTCTAGAGCTGGTAATTAAACGTTATAATACAACTACTATTTTCCGATTCATCGTTCT 180
                ******************** ***********  **** **************** ****

MYB113^WT       TTCTTTCTTAAATTTCGTGTTAATACATGTGGGTCTAAATAGATGTCGGAAAAGCTGTAG 237
MYB113^Sly      TTCTTTCTTAAATTTCATGTTAATACATGCAGGTCTAAATAGATGTCGAAAAAGCTGTAG 239
MYB113^Abg      TTCTTTCTTAAATTTCATGTTAATACATGCAGGTTTAAATAGATGTCGGAAAAGCTGTAG 240
                **************** ************  *** ************* ***********

MYB113^WT       ACTGAGATGGCTAAATTATCTACGCCACATATCAAAGAGAGGTGTCTTTGAACGGGATGA 297
MYB113^Sly      ACTGAGATGGCTAAATTATCTAAGACCACATATCAAAAGAGGTGACTTTGAACCAGATGA 299
MYB113^Abg      ACTGAGATGGCTAAATTATCTAAGACCACATATCAAAAGAGGTGACTTTGAACCAGATGA 300
                ********************* * *        ** ******* ********  *****

MYB113^WT       AGTGGATTTAATTTTGACGCTTCATTAGCTCTTAGGCAACAGGCAATTAATTAGTACTTT 357
MYB113^Sly      AGTGGATCTCATTTTGAGGCTTCATAAGCTCTTAGGCAACAGGCAATTAATTAGTACTTT 359
MYB113^Abg      AGTGGATCTCATTTTGAGGCTTCATAAGCTCTTAGGCAACAGGCAATTAATTAGTACTTT 360
                ******* * ******* ******* **********************************

MYB113^WT       ATAATGTTTAATACTTTAATCTCATATATATTAGTTACACTCGATTACATAAATTATACT 417
MYB113^Sly      ATAATATTTAATACTTTAATTTCATATATATTAATTACACTCAATTACGTAAATTATATT 419
MYB113^Abg      ATAATATTTAATACTTTAATTTCATATATATTAATTACACTCAATTACGTAAATTATATT 420
                ***** ************** ************ ******** ***** ********* *

MYB113^WT       ACTATGTATTATATAACATGTCCGCTCATGCAATACTATA-------------------- 457
MYB113^Sly      ACTATGTATTACTCCCTATGTTCCTATTTAGTTGTCCACTTTAAGAAATGACACACGTAT 479
MYB113^Abg      ACTATGTATTACTGTCTATGTCCCTATTTAGTTGTCCACTTTAAGAAATGATACACGTAT 480
                ***********      **** *     *      * *

MYB113^WT       ------------------------------------------------------------ 457
MYB113^Sly      TAAAATAGCAATAATTAACATAGTGAAGTTACAATTTTATCCTTATTAATTATGGTTTCA 539
MYB113^Abg      TAAAATAGCAACAATTAACATAGTGAAGTTACAATTTTATCCTTATTAATTATGGTTTCA 540

MYB113^WT       ------------------------------------------------------------ 457
MYB113^Sly      AAAAGGATGAATTAAAACTTAAAAATTTTCAAGAAGTTTAATTAGGGTATAATAGAAAA- 598
MYB113^Abg      AAAAGTTTGAATTAAAACTTGAAAATTTTCAAGAAGTTTAAATAAGGGTATAATAGATTA 600

MYB113^WT       ------------------------------------------------------------ 457
MYB113^Sly      -AAAAATTGTCTTTTTTTTATTTGTTAAAATGAACAAGTAAATAGAGACAGCTAAAAGAG 657
MYB113^Abg      TTTTTTTTGTCTTTTCTTAATTTGTCAAAATGGACAAGTAAATAGGGACATGTAAAATAG 660

MYB113^WT       ------------------------------------------------------------ 457
MYB113^Sly      GAAACATGGACAAGTAAATAGGGACAGATGGAGTATATAACATGTCCACTCATGCGGTAC 717
MYB113^Abg      GAAATATGGACAAATAAATAGGAACAAATGGAGTGTATAACATGTCCACTCATGTAGTCA 720

MYB113^WT       ------------------------------------------------------------ 457
MYB113^Sly      TATTAT------ATGTTGTGAAATGTGTAATCATATCATCTAAAAATTACGTTATTTTGT 771
MYB113^Abg      TGCGGTACTATATGTTTGTAAAATGTGTAATCATATCATCTAAAAATTACGTTATTTTGT 780

MYB113^WT       ----------------------------------------TGTTGTAAAATGTATAACCA 477
MYB113^Sly      GTAGATGGTCACTTATTGCTGATAGACTTCCAGGAAGGACTTGTAAAATG--TGTAATCA 829
MYB113^Abg      GTAGATGGTCACTTGTTGCTGGTAGACTTCCAGGAAGGACTTGTAAAATGTGTGTAATCA 840
                                        *  *  **    * *** **
```

```
MYB113^WT    TATCATCGAAAGATTATGTTATTTCGTGTAGGTGGACACTTATTGCTGGTAGACTTCCGG    537
MYB113^Sly   TATCATCTAAAAATTACGTTATTTTGTGTAGGTGGTCACTTATTGCTGGTAGACTTCCAG    889
MYB113^Abg   TATCATCTAAAAATTACGTTATTTTGTGTAGGTGGTCACTTATTGCTGGTAGACTTCCAG    900
             ******* *** **** ******* ********** ********************** *

MYB113^WT    GAAGGACAGCAAACGATATAAAAAACTACTGGAATACTCGTCTTCTAAG-AAGTTAAATA    596
MYB113^Sly   GAAGGACAGCAAACGATGTGAAAAATTACTGGAATACTCGTCTTCTAAGGAAGTTAAATA    949
MYB113^Abg   GAAGGACAGCAAACGATGTGAAAAATTACTGGAATACTCGTCTTCTAAGGAAGTTAAATA    960
             ***************** * ***** *********************** **********

MYB113^WT    CTAATATTACGAATAATGAAATAACAAAACCTCAACCTCAGACGTTATCATCAAATGCAA    656
MYB113^Sly   CTAATATTACAAAGAATGAAATAATAAAACCTCAACCTCGGACCTTATCATCAAATGCAA    1009
MYB113^Abg   CTAATATTACAAAGAATGAAATAATAAAACCTCAACCTCGGACCTTATCATCAAATGCAA    1020
             ********** ** ********** ************** *** ****************

MYB113^WT    AGAATGTTTCTTTGTGCAACAACAAAAGTATGATCACAAACACATTAGATAAAGATGATG    716
MYB113^Sly   AGAATGTTTCTTGGTGCAACAACAAAAGTATGATCACAAACACATTAGACAAAGATGACG    1069
MYB113^Abg   AGAATGTTTCTTGGTGCAACAACAAAAGTATGATCACAAACACATTAGACAAAGATGACA    1080
             ************ ************************************ ********

MYB113^WT    AACAACGCAACAAAGAAATCGTAGTAAATATTTGGGAGAAGCCAACGGGAGAAACAACAA    776
MYB113^Sly   AACAACGCAACAAAGAAATCGTAGTAAGTATTTGTGAGAAGCCAA---------------    1114
MYB113^Abg   AACAACGGAACAAAGAAATCGTAGTAAATATCTGTGAGAAGCCAA---------------    1125
             ******* ******************* *** ** **********

MYB113^WT    CATTGCCATCGTCTATAGACGATGACAGAGTTGAATGGTGGACAAGCTTACTGGAAAATT    836
MYB113^Sly   ---CATCATCGTCTATAGACGATGACGGAGTTGAATGGTGGACAAGTTTACTGGAAAATT    1171
MYB113^Abg   ---CATCATCGTCTATAGACGATGACGGAGTTAAATGGTGGACAAGTTTACTGGAAAATT    1182
                   ******************** ***** ************* *************

MYB113^WT    GTAAAGAAATTGAGGATGAAGAAGCAGTAGTATTGAGCTTTGAAGAAGAAACTAAGTTAT    896
MYB113^Sly   GTAATGAAATTGAGGATGAAGAAGCAGCAGTATTGAGATTTGAAGAAGAAAATAAGTCGT    1231
MYB113^Abg   GTAATGAAATTGAGGATGAAGAAGCAGCAGTATTGAGCTTTGAAGAAGAAAATAAGTTGT    1242
             **** ********************** ********* ************* *****  *

MYB113^WT    TATCAAATTTGTTGCATGAGGAAAATAATAATTCACCAATCATGCTACAAGGAGAAAGTC    956
MYB113^Sly   TACCAAATTTGTTGCATGAGGAAAG---TAATTCACCAATCATGCTACAAGGAGAAAGTG    1288
MYB113^Abg   TACCAAATTTGTTGCATGAGGAAAA---TAATTCACCAATCATGCTACAAGGAGAAAGTG    1299
             ** *********************    *******************************

MYB113^WT    ATGGTTGGGATATTGACTTATTATGGAATCTACTTAATTAA    997
MYB113^Sly   ATGGTTGGGATATTGACTTATTATGGAATCTACTTAATTAA    1329
MYB113^Abg   ATGGTTGGGATATTGACTTATTATGGAATCTACTTAATTAA    1340
             *****************************************
```

Fig. S13. ClustalW alignment of the gene *MYB113* sequenced in *Aubergine* (Abg), *S. lycopersicoides* (Sly) and wild type (WT) plants. Exons are highlighted in grey. The symbol * indicates perfect alignment.

a

> *THM2 7*[Abg]

```
ATGGGAAGGTCACCTTGTTGTGAGAAGGCACATACAAACAAAGGAGCATGGACTAAAGAAGAAGATGAAAGACTTATTTCTTACATTAGAGCTCATGGTG
AAGGTTGTTGGAGGTCTCTTCCTAAAGCTGCTGGACTTCTCCGATGCGGTAAAAGTTGTCGTCTCCGATGGATTAATTACTTAAGACCTGACCTTAAACG
TGGTAACTTTACTGAAGAAGAAGATGAACTCATTATTAAACTCCATAGCCTCCTTGGAAACAAGTATGTTTAACATTTCTATGTTATTTTATTTTGTCCT
ACTAATTAAATCCTTAACGAGCTGAGACAGATTCAGGATTTAAATTTGATCTGACTCAATATGAGATGATTATATGGATCACTAGTACTAATTATGTTTT
TTGTTTCCTTATGATAGGTGGTCGCTTATAGCAGGAAGATTACCAGGAAGAACAGATAACGAGATAAAAAATTACTGGAACACACATATAAGACGAAAGC
TTTTGAGTCGAGGTATTGATCCAACAACACATCGATCAATCAATGATCCTACTACAATACCAAAAGTTACAACCATTACTTTTGCTGCTGCTGCTGCTCA
TGAAAATATTAAAGATATTGATCAACAAGATGAGGTGATAAATATCAAAGCTGAATTCATTGAAACAAGCAAAGAATCAGATAATAATGAAATAAAACAA
GAAAAGTCATCATGTCTTCCTGACTTAAATCTTGAACTCAGAATTAGTCCTCCACATCATCAACAACTCGATCATCATCATCATCATCATCATCGTCATC
ATCAACGTTCAAGCTCTTTATGTTTTACATGTAGTTTGGGAATTCAAAATAGTAAAGATTGCAGTTGTGGAAGTGAAAGTAATGGAAATGGATGGAGTAA
TAATATTGTAAGTATGAACATTATGGCTGGTTATGACTTTTTGGGCTTGAAGACTAATGGTCTTTTGGACTATAGAACTTTGGAAACTAAGTGA
```

> *THM2 7*[Sly]

```
ATGGGAAGGTCACCTTGTTGTGAGAAGGCACATACAAACAAAGGAGCATGGACTAAAGAAGAAGATGAAAGACTTATTTCTTACATTAGAGCTCATGGTG
AAGGTTGTTGGAGGTCTCTTCCTAAAGCTGCTGGACTTCTCCGATGCGGTAAAAGTTGTCGTCTCCGATGGATTAATTACTTAAGACCTGACCTTAAACG
TGGTAACTTTACTGAAGAAGAAGATGAACTCATTATCAAACTCCATAGCCTCCTTGGAAACAAGTATGTTTAACATTTCTATCTTGTTTTATTTTGTCCT
ACTAATTAAATCCTTAACGAGCTGAGACAGATTCAGGATTTAAATTTGATCTGACTCAATATGAGATGATTATATGGATCACTAGTACTAATTATGTTTT
TTGTTTCCTTATGATAGGTGGTCGCTTATAGCAGGAAGATTACCAGGAAGAACAGATAACGAGATAAAAAATTATTGGAACACACATATAAGACGAAAGC
TTTTGAGTCGAGGTATTGATCCAACAACACATCGATCAATCAATGATCCTACTACAATACCAAAAGTTACAACCATTACTTTTGCTGCTGCTGCTGCTGC
TCATGAAAATATTAAAGATATTGATCAACAAGATGAGGTGATAAATATCAAAGCTGAATTCATTGAAACAAGCAAAGAATCAGATAATAATGAAATAAAA
CAAGAAAAGTCATCATGTCTTCCTGACTTAAATCTTGAACTCAGAATTAGTCCTCCACATCATCAACAACTCGATCATCATCATCATCATCATCATCGTC
ATCATCAACGTTCAAGCTCTTTATGTTTTACATGTAGTTTGGGAATTCAAAATAGTAAAGATTGCAGTTGTGGAAGTGAAAGTAATGGAAATGGATGGAG
TAATAATATTGTAAGTATGAACATTATGGCTGGTTATGACTTTTTGGGCTTGAAGACTAATGGTCTTTTGGACTATAGAACTTTGGAAACTAAGTGA
```

> *THM2 7*[WT]

```
ATGGGAAGGTCACCTTGTTGTGAGAAGGCACATACAAACAAAGGAGCATGGACTAAAGAAGAAGATGAAAGACTAATTTCTTACATTAGAGCTCATGGTG
AAGGTTGTTGGAGGTCTCTTCCTAAAGCTGCTGGACTTCTTCGATGCGGTAAAAGTTGTCGTCTCCGATGGATTAATTACTTAAGACCTGACCTTAAACG
TGGTAACTTTACTGAAGAAGAAGATGAACTCATTATCAAACTCCATAGCCTCCTTGGAAACAAGTATGTTTAACATTTCTATCTTATTTTATTTTGTCCT
ACTAGTTAAATCGTTAACGAGATGAGACAGATTCAGGATTTAAATTTGACCTGACTCAAGGTGGTTATATGGATCACTAGTAGTAATTATGTTTTTTTGT
TTCCTTATGATAGGTGGTCGCTTATAGCAGGAAGATTACCAGGAAGAACAGATAACGAGATAAAAAACTATTGGAACACACATATAAGACGAAAGCTCTT
GAGTCGAGGTATTGATCCAACAACACATAGATCAATCAATGATCCTACTACAATACCAAAAGTTACAACGATTACTTTTGCTGCTGCTCATGAAAATATT
AAAGATATTGATCAACAAGATGAGATGATAAATATCAAAGCTGAATTCGTTGAAACAAGCAAAGAATCAGATAATAATGAAATAATTCAAGAAAAGTCAT
CATCATGTCTTCCTGACTTAAATCTTGAACTCAGAATTAGTCCTCCACATCATCAACAACTCGATCATCATCGTCATCATCAACGATCAAGCTCTTTATG
TTTTACATGTAGTTTGGGAATTCAAAATAGTAAAGATTGCAGTTGTGGAAGTGAAAGTAATGGAAATGGATGGAGTAATAATATGGTAAGTATGAACATT
ATGGCTGGTTATGACTTTTTGGGCTTGAAGACTAATGGTCTTTTGGACTATAGAACTTTGGAAACTAAGTGA
```

b

```
THM27WT    MGRSPCCEKAHTNKGAWTKEEDERLISYIRAHGEGCWRSLPKAAGLLRCGKSCRLRWINY
THM27Abg   MGRSPCCEKAHTNKGAWTKEEDERLISYIRAHGEGCWRSLPKAAGLLRCGKSCRLRWINY
THM27Sly   MGRSPCCEKAHTNKGAWTKEEDERLISYIRAHGEGCWRSLPKAAGLLRCGKSCRLRWINY

THM27WT    LRPDLKRGNFTEEEDELIIKLHSLLGNKWSLIAGRLPGRTDNEIKNYWNTHIRRKLLSRG
THM27Abg   LRPDLKRGNFTEEEDELIIKLHSLLGNKWSLIAGRLPGRTDNEIKNYWNTHIRRKLLSRG
THM27Sly   LRPDLKRGNFTEEEDELIIKLHSLLGNKWSLIAGRLPGRTDNEIKNYWNTHIRRKLLSRG

THM27WT    IDPTTHRSINDPTTIPKVTTITF---AAAHENIKDIDQQDEmINIKAEFvETSKESDNNE
THM27Abg   IDPTTHRSINDPTTIPKVTTITF-AAAAAHENIKDIDQQDEVINIKAEFIETSKESDNNE
THM27Sly   IDPTTHRSINDPTTIPKVTTITFaAAAAAHENIKDIDQQDEVINIKAEFIETSKESDNNE

THM27WT    IiQEKsSSCLPDLNLELRISPPHHQQLD-----HHRHHQRSSSLCFTCSLGIQNSKDCSC
THM27Abg   IkQEK-SSCLPDLNLELRISPPHHQQLDHHHHHHHRHHQRSSSLCFTCSLGIQNSKDCSC
THM27Sly   IkQEK-SSCLPDLNLELRISPPHHQQLDHHHHHHHRHHQRSSSLCFTCSLGIQNSKDCSC

THM27WT    GSESNGNGWSNNmVSMNIMAGYDFLGLKTNGLLDYRTLETK
THM27Abg   GSESNGNGWSNNIVSMNIMAGYDFLGLKTNGLLDYRTLETK
THM27Sly   GSESNGNGWSNNIVSMNIMAGYDFLGLKTNGLLDYRTLETK
```

Fig. S14. Sequence analysis of the gene and protein THM27 in *Aubergine* (Abg), *S. lycopersicoides* (Sly) and wild type (WT) plants. **a** Sequence of the gene *THM27* in *Aubergine* (Abg), *S. lycopersicoides* (Sly) and wild type (WT) plants. Exons are highlighted in grey. **b** Sequence of the protein THM27 in *Aubergine* (Abg), *S. lycopersicoides* (Sly) and wild

type (WT) plants. R2 and R3 MYB domains are underlined. The [DE]Lx2[RK]x3Lx6Lx3R motif containing the bHLH-binding site [45] is boxed in blue, and the EAR motif sequence, conserved in TFs known to function as negative regulators [46], is boxed in red.

```
THM27^WT     ATGGGAAGGTCACCTTGTTGTGAGAAGGCACATACAAACAAAGGAGCATGGACTAAAGAA  60
THM27^Sly    ATGGGAAGGTCACCTTGTTGTGAGAAGGCACATACAAACAAAGGAGCATGGACTAAAGAA  60
THM27^Abg    ATGGGAAGGTCACCTTGTTGTGAGAAGGCACATACAAACAAAGGAGCATGGACTAAAGAA  60
             ************************************************************

THM27^WT     GAAGATGAAAGACTAATTTCTTACATTAGAGCTCATGGTGAAGGTTGTTGGAGGTCTCTT  120
THM27^Sly    GAAGATGAAAGACTTATTTCTTACATTAGAGCTCATGGTGAAGGTTGTTGGAGGTCTCTT  120
THM27^Abg    GAAGATGAAAGACTTATTTCTTACATTAGAGCTCATGGTGAAGGTTGTTGGAGGTCTCTT  120
             ************** *********************************************

THM27^WT     CCTAAAGCTGCTGGACTTCTTCGATGCGGTAAAAGTTGTCGTCTCCGATGGATTAATTAC  180
THM27^Sly    CCTAAAGCTGCTGGACTTCTCCGATGCGGTAAAAGTTGTCGTCTCCGATGGATTAATTAC  180
THM27^Abg    CCTAAAGCTGCTGGACTTCTCCGATGCGGTAAAAGTTGTCGTCTCCGATGGATTAATTAC  180
             ******************** ***************************************

THM27^WT     TTAAGACCTGACCTTAAACGTGGTAACTTTACTGAAGAAGAAGATGAACTCATTATCAAA  240
THM27^Sly    TTAAGACCTGACCTTAAACGTGGTAACTTTACTGAAGAAGAAGATGAACTCATTATCAAA  240
THM27^Abg    TTAAGACCTGACCTTAAACGTGGTAACTTTACTGAAGAAGAAGATGAACTCATTATTAAA  240
             ******************************************************** ***

THM27^WT     CTCCATAGCCTCCTTGGAAACAAGTATGTTTAACATTTCTATCTTATTTTATTTTGTCCT  300
THM27^Sly    CTCCATAGCCTCCTTGGAAACAAGTATGTTTAACATTTCTATCTTGTTTTATTTTGTCCT  300
THM27^Abg    CTCCATAGCCTCCTTGGAAACAAGTATGTTTAACATTTCTATGTTATTTTATTTTGTCCT  300
             ****************************************** ** **************

THM27^WT     ACTAGTTAAATCGTTAACGAGATGAGACAGATTCAGGATTTAAATTTGACCTGACTCAAG  360
THM27^Sly    ACTAATTAAATCCTTAACGAGCTGAGACAGATTCAGGATTTAAATTTGATCTGACTCAAT  360
THM27^Abg    ACTAATTAAATCCTTAACGAGCTGAGACAGATTCAGGATTTAAATTTGATCTGACTCAAT  360
             **** ******* ******** *************************** *********

THM27^WT     GTG-----GTTATATGGATCACTAGTAGTAATTATGTTTTTTTGTTTCCTTATGATAGGT  415
THM27^Sly    ATGAGATGATTATATGGATCACTAGTACTAATTATG-TTTTTTGTTTCCTTATGATAGGT  419
THM27^Abg    ATGAGATGATTATATGGATCACTAGTACTAATTATG-TTTTTTGTTTCCTTATGATAGGT  419
              **      ****************** ******** ***********************

THM27^WT     GGTCGCTTATAGCAGGAAGATTACCAGGAAGAACAGATAACGAGATAAAAAACTATTGGA  475
THM27^Sly    GGTCGCTTATAGCAGGAAGATTACCAGGAAGAACAGATAACGAGATAAAAAATTATTGGA  479
THM27^Abg    GGTCGCTTATAGCAGGAAGATTACCAGGAAGAACAGATAACGAGATAAAAAATTACTGGA  479
             **************************************************** ** ****

THM27^WT     ACACACATATAAGACGAAAGCTCTTGAGTCGAGGTATTGATCCAACAACACATAGATCAA  535
THM27^Sly    ACACACATATAAGACGAAAGCTTTTGAGTCGAGGTATTGATCCAACAACACATCGATCAA  539
THM27^Abg    ACACACATATAAGACGAAAGCTTTTGAGTCGAGGTATTGATCCAACAACACATCGATCAA  539
             ********************** ****************************** ******

THM27^WT     TCAATGATCCTACTACAATACCAAAAGTTACAACGATTACTTTTGCTGC---------TG  586
THM27^Sly    TCAATGATCCTACTACAATACCAAAAGTTACAACCATTACTTTTGCTGCTGCTGCTGCTG  599
THM27^Abg    TCAATGATCCTACTACAATACCAAAAGTTACAACCATTACTTTTGCTGCTGC---TGCTG  596
             ********************************** **************         **

THM27^WT     CTCATGAAAATATTAAAGATATTGATCAACAAGATGAGATGATAAATATCAAAGCTGAAT  646
THM27^Sly    CTCATGAAAATATTAAAGATATTGATCAACAAGATGAGGTGATAAATATCAAAGCTGAAT  659
THM27^Abg    CTCATGAAAATATTAAAGATATTGATCAACAAGATGAGGTGATAAATATCAAAGCTGAAT  656
             ************************************** *********************

THM27^WT     TCGTTGAAACAAGCAAAGAATCAGATAATAATGAAATAATTCAAGAAAAGTCATCATCAT  706
THM27^Sly    TCATTGAAACAAGCAAAGAATCAGATAATAATGAAATAAAACAAGAAAAGT---CATCAT  716
THM27^Abg    TCATTGAAACAAGCAAAGAATCAGATAATAATGAAATAAAACAAGAAAAGT---CATCAT  713
             ** ************************************  **********   ******

THM27^WT     GTCTTCCTGACTTAAATCTTGAACTCAGAATTAGTCCTCCACATCATCAACAACTCGATC  766
THM27^Sly    GTCTTCCTGACTTAAATCTTGAACTCAGAATTAGTCCTCCACATCATCAACAACTCGATC  776
THM27^Abg    GTCTTCCTGACTTAAATCTTGAACTCAGAATTAGTCCTCCACATCATCAACAACTCGATC  773
             ************************************************************

THM27^WT     AT---------------CATCGTCATCATCAACGATCAAGCTCTTTATGTTTTACATGTA  811
THM27^Sly    ATCATCATCATCATCATCATCGTCATCATCAACGTTCAAGCTCTTTATGTTTTACATGTA  836
THM27^Abg    ATCATCATCATCATCATCATCGTCATCATCAACGTTCAAGCTCTTTATGTTTTACATGTA  833
             **               ***************** *************************
```

```
THM27^WT    GTTTGGGAATTCAAAATAGTAAAGATTGCAGTTGTGGAAGTGAAAGTAATGGAAATGGAT  871
THM27^Sly   GTTTGGGAATTCAAAATAGTAAAGATTGCAGTTGTGGAAGTGAAAGTAATGGAAATGGAT  896
THM27^Abg   GTTTGGGAATTCAAAATAGTAAAGATTGCAGTTGTGGAAGTGAAAGTAATGGAAATGGAT  893
            ************************************************************

THM27^WT    GGAGTAATAATATGGTAAGTATGAACATTATGGCTGGTTATGACTTTTTGGGCTTGAAGA  931
THM27^Sly   GGAGTAATAATATTGTAAGTATGAACATTATGGCTGGTTATGACTTTTTGGGCTTGAAGA  956
THM27^Abg   GGAGTAATAATATTGTAAGTATGAACATTATGGCTGGTTATGACTTTTTGGGCTTGAAGA  953
            ************* **********************************************

THM27^WT    CTAATGGTCTTTTGGACTATAGAACTTTGGAAACTAAGTGA           972
THM27^Sly   CTAATGGTCTTTTGGACTATAGAACTTTGGAAACTAAGTGA           997
THM27^Abg   CTAATGGTCTTTTGGACTATAGAACTTTGGAAACTAAGTGA           994
            *****************************************
```

Fig. S15. ClustalW alignment of the gene *THM27* sequenced in *Aubergine* (Abg), *S. lycopersicoides* (Sly) and wild type (WT) plants. Exons are highlighted in grey. The symbol * indicates perfect alignment.

a

>*AN2*^{Abg} *cds*
ATGAATACTCCTATGTGTGCATCGTTGGGAGTTAGGAAAGGTTCATGGACTGAACAAGAAGATTTTCTTTTAAGAAATTGCATTCAAAAATATGGTGAAG
GAAAGTGGCATCTTGTTCCTGCTAGAGCTGGTTTGAATCGATGTCGAAAGAGTTGCAGACTAAGGTGGCTAAATTATCTAAGGCCACATATCAAGAGAGG
TGACTTTGCTCCAGATGAAGTAGATCTCATCTTGAGACTTCATAAACTCTTAGGCAATAGGTGGTCACTTATTGCTGGTAGACTTCCAGGAAGGACTGCA
AATGATGTTAAGAACTATTGGAACACTCACTTTCATAAGAAGTTAAATATTATTGCTCCTCATCTTCATCCTCGTCCTCGTCCTCATCCTCATCTACAGA
TTAAGCATAAGAGCATCGCGGTTACTAAGAATGAAATAATAAAACCTCAACCTCGGAACTTCTCAAACGTTAATAAGAATATTTCTCATTGGTGCAACAA
CAACAAAAGTATGATCACAAACACATTAGACAAAGATGACAAACGTTGCAAGGAGATGGTAGTAAATATTTGTGAGAAGCCAATAGGAGAAAATACATCG
TCGATAGACGATGGAGTTGAATGGTGGACAAATTTACTGGTAAATTGCAATGAAATTGAAGAAGAAGCTGTTGTTACAAATTTTGAAAAGACACCAACAA
TGTTGTTAAATGAGGAAATATCACCACCGTTAATAATTAATGGTGAAGGCAACTCCATGCAACAAGGACAAACTCATGATAATTGGGATGACTTTTCAAC
TGATATTGACTTATGGAATCTACTTAATTAA

>*ANT1*^{Abg} *cds*
ATGAACAGTACATCTATGTCTTCATTGGGAGTGAGAAAAGGTTCTTGGACTGATGAAGAAGATTTTCTTTTAAGAAAATGTATTAACAAGTATGGTGAAG
GAAAATGGCATCTTGTTCCCATGAGAGCTGGTCTGAATAGATGTCGGAAAAGTTGTAGATTGAGGTGGCTGAATTATCTAAGGCCACATATCAAAAGAGG
TGACTTTGAACAAGATGAAGTGGATCTCATTTTGAGGCTTCATAAGCTCTTAGGCAACAGATGGTCACTTATTGCAGGTAGACTTCCAGGAAGGACAGCT
AACGACATAAAAAACTATTGGAACACTAACCTTCTAAGGAAGTTAAATACTAGTAAAATTGTTCCTCGTGAAAAGATTAACAATAAGTGTGGAGAAATTA
GTACTAAGATTGAAATAATAAAACCTCAACCTAGGAAGTATTTCTCAAGCACAAAGATGAATATTGTAATTTTTGATGAGGAGGAACATTGCAAGGAAAT
AATAAGTGAGAAGCAAACTCCAGATGCATCGATGGACAACGTAGATCAATGGTGGACAAATTTACTGGAAAATTGCAATGACGATGTTGAAGAAGATGAA
GAGGTTGTAATTAATTATGAAAAAACACTAACAAGTTTGTTACATGAAGAAATATCACCACCATTAAATGGTGAAGGTAACTCTATAATGCAACAAGGAC
AAACAAGTCATGATAGTTGGGGTGACTTTTCTCTTAATTTACCACCCATGCAACAAGGAGTACAAAATGATGATTGGGATGATTTTTCTGCTGAAATTGA
CTTATGGAATCTACTTGATTAA

>*ANT1like*^{Abg} *cds*
ATGAACAGTACATCTATGTCTTCTTTGGGAATAAGAAAAGGTTCATGGACTGAAGAAGAAGATTTCCTCTTAAGGAAATGTATCAACAAGTATGGTGAAG
GAAAGTGGCATCTTGTTCCCATAAGAGCTGGTCTGAATAGATGTCGGAAAAGTTGTAGACTGAGGTGGCTGAATTATCTAAGGCCACATATCAAGAGAGG
TGACTTTGAACAAGATGAAGTGGATCTCATTTTGAGGCTTCATAAGCTCTTAGGCAACAGATGGTCACTTATTGCTGGTAGACTTCCAGGAAGGACAGCA
AACGATGTGAAAAACTATTGGAACACAAACCTTCTAAGGAAGTTAAATACTACTAAAATTGTTGCTCGTGAAAAGATTAAGAGTAAGCGTGGAGAAATTA
GTGATAAGATTGAAATAATAAAACCTCAACCTAGGAAGTTCATATCGAACACAAAGAAGAATATTACAAGCAATATTGTAATTGAAGACAAAGAGGAACA
ATGTAAGGAAATAACAAGTGAGAAGCAAACTAGAGATGCATCGATAGACAACGGAGATGAATGGTGGGAAAATTTACTGGAAAATTGCAACGACGATGTT
GAAGAAGATGAAGAGGTTGTAATTAATTATGAAAAAACACTAACAAGTTTGTTACATGAGGAAATAACACCACCATTAAATGGTGGAGGTAACTTCATGC
AACAAGGACAAAGTGATGGTTGGGATGATTTTTTTGTTGATATTGATATATGGGATTTACTTAATTAA

>*AN2like*^{Abg} *cds*
ATGAATATTGCCAAGACATTGGGAGTAAGAAAAGGTTCATGGACTGAAGAAGAAGATACTCTTTTGAGGAAATGTATTAACAAGTATGGAGAAGGAAAGT
GGCATCTTGTTCCTTCTAGAGCTGGTCTAAATCGATGTCGAAAGAGTTGTAGACTGAGGTGGTTGAATTATCTAAGGCCACATATCAAGAGAGGTGACTT
TGCTCCGGATGAAATAGATCTCATTTTAAGACTTCACAAGCTTCTAGGCAATAGGTGGTCACTTATTGCTGGGAGACTTCCAGGAAGAACAGCAAACGAT
GTGAAGAACTATTGGAACACACACCTACACAAGAAGTTATTAATAACTCCTCAGATACAAGAGAATAAGTACAATAATACCCTCAAGATTATCACTGAAA
GCACTATACTACGACCACGACCTCGAACCTTTTCAAGTGAAAATAATATTTCTTGGTGCACTAACAATAGTATGATCACAAACACATTATACAAAGATGA
CGAACAACGTAACAAAGAAATCGTAGTAAATATTTGTGAGAAGCCAACAAGAGAAACTCCGTCATCGTCTATAGACGATGACGGAGTTAAATGGTGTACA
AATTTACTGGAAAATTGGAAAGAATTTGAGGAAGCAGCAGCAGCAGTATTGAGCTTTGAGGAAGAAAATAATTTGTTACCAAATTTGTTGAATGAGGGAA
ATAATTCAACGACCATGCAACATGGAGAAAATGATGACTTTTCAGTTGATATTGACCTATGGAATCTATTTAATTAG

>*MYB113*^{Abg} *cds*
ATGAATACTCCTATGTGTACATCATTAGGAGTAATTAGGAAAGGTTCATGGACTGAACAAGAAGATTTTCACTTGAGAAAATGCATTCAAAAATATGGTG
AAGGAAAGTGGAATCTTGTTCCTTCTAGAGCTGGTATAAATAGATGTCGGAAAAGCTGTAGACTGAGATGGCTAAATTATCTAAGACCACATATCAAAAG
AGGTGACTTTGAACCAGATGAAGTGGATCTCATTTTGAGGCTTCATAAGCTCTTAGGCAACAGGTGGTCACTTATTGCTGGTAGACTTCCAGGAAGGACA
GCAAACGATGTGAAAAATTACTGGAATACTCGTCTTCTAAGGAAGTTAAATACTAATATTACAAAGAATGAAATAATAAAACCTCAACCTCGGACCTTAT
CATCAAATGCAAAGAATGTTTCTTGGTGCAACAACAAAAGTATGATCACAAACACATTAGACAAAGATGACAAACAACGGAACAAAGAAATCGTAGTAAA
TATCTGTGAGAAGCCAACATCATCGTCTATAGACGATGACGGAGTTAAATGGTGGACAAGTTTACTGGAAAATTGTAATGAAATTGAGGATGAAGAAGCA
GCAGTATTGAGCTTTGAAGAAGAAAATAAGTTGTTACCAAATTTGTTGCATGAGGAAAATAATTCACCAATCATGCTACAAGGAGAAAGTGATGGTTGGG
ATATTGACTTATTATGGAATCTACTTAATTAA

b

AN2^{Abg}

MNTPMCASLGVRKGSWTEQEDFLLRNCIQKYGEGKWHLVPARAGLNRCRKSCRLRWLNYLRPHIKRGDFAPDEVDLILRLHKLLGNRWSLIAGRLPGRTA
NDVKNYWNTHFHKKLNIIAPHLHPRPRPHPHLQIKHKSIAVTKNEIIKPQPRNFSNVNKNISHWCNNNKSMITNTLDKDDKRCKEMVVNICEKPIGENTS
SIDDGVEWWTNLLVNCNEIEEEAVVTNFEKTPTMLLNEEISPPLIINGEGNSMQQGQTHDNWDDFSTDIDLWNLLN

ANT1^{Abg}

MNSTSMSSLGVRKGSWTDEEDFLLRKCINKYGEGKWHLVPMRAGLNRCRKSCRLRWLNYLRPHIKRGDFEQDEVDLILRLHKLLGNRWSLIAGRLPGRTA
NDIKNYWNTNLLRKLNTSKIVPREKINNKCGEISTKIEIIKPQPRKYFSSTKMNIVIFDEEEHCKEIISEKQTPDASMDNVDQWWTNLLENCNDDVEEDE
EVVINYEKTLTSLLHEEISPPLNGEGNSIMQQGQTSHDSWGDFSLNLPPMQQGVQNDDWDDFSAEIDLWNLLD

ANT1like^{Abg}

MNSTSMSSLGIRKGSWTEEEDFLLRKCINKYGEGKWHLVPIRAGLNRCRKSCRLRWLNYLRPHIKRGDFEQDEVDLILRLHKLLGNRWSLIAGRLPGRTA
NDVKNYWNTNLLRKLNTTKIVAREKIKSKRGEISDKIEIIKPQPRKFISNTKKNITSNIVIEDKEEQCKEITSEKQTRDASIDNGDEWWENLLENCNDDV
EEDEEVVINYEKTLTSLLHEEITPPLNGGGNFMQQGQSDGWDDFFVDIDIWDLLN

AN2like^{Abg}

MNIAKTLGVRKGSWTEEEDTLLRKCINKYGEGKWHLVPSRAGLNRCRKSCRLRWLNYLRPHIKRGDFAPDEIDLILRLHKLLGNRWSLIAGRLPGRTAND
VKNYWNTHLHKKLLITPQIQENKYNNTLKIITESTILRPRPRTFSSENNISWCTNNSMITNTLYKDDEQRNKEIVVNICEKPTRETPSSSIDDDGVKWCT
NLLENWKEFEEAAAAVLSFEEENNLLPNLLNEGNNSTTMQHGENDDFSVDIDLWNLFN

MYB113^{Abg}

MNTPMCTSLGVIRKGSWTEQEDFHLRKCIQKYGEGKWNLVPSRAGINRCRKSCRLRWLNYLRPHIKRGDFEPDEVDLILRLHKLLGNRWSLIAGRLPGRT
ANDVKNYWNTRLLRKLNTNITKNEIIKPQPRTLSSNAKNVSWCNNKSMITNTLDKDDKQRNKEIVVNICEKPTSSSIDDDGVKWWTSLLENCNEIEDEEA
AVLSFEEENKLLPNLLHEENNSPIMLQGESDGWDIDLLWNLLN

Fig. S16. cds and protein sequences of the R2R3 MYB transcription factors belonging to the cluster of chromosome 10 cloned in *Aubergine* (*Abg*) plants. **a** cds of the *R2R3 MYB* genes belonging to the cluster of chromosome 10 cloned in *Aubergine* (*Abg*) plants. **b** Sequences of the relative R2R3 MYB transcription factors. R2 and R3 MYB domains are underlined. The [DE]Lx2[RK]x3Lx6Lx3R motif containing the bHLH-binding site [45] is in blue, and the ANDV motif sequence, conserved in TFs known to function as positive regulators [46], is in red.

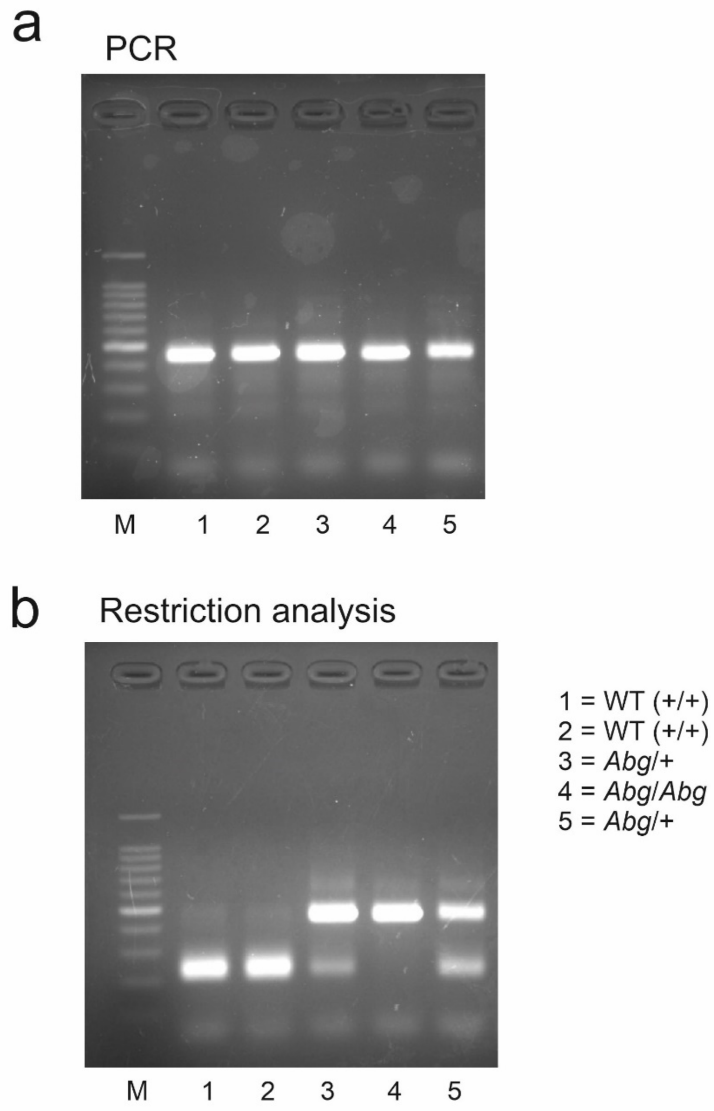

Fig. S17. CAPS marker for the gene *AN2like*. **a** Agarose gel electrophoresis following PCR amplification of a specific region within the gene. **b** Agarose gel electrophoresis following the restriction analysis of the amplicon with the endonuclease Spe I. In both a and b M indicates a 100 bp DNA ladder.

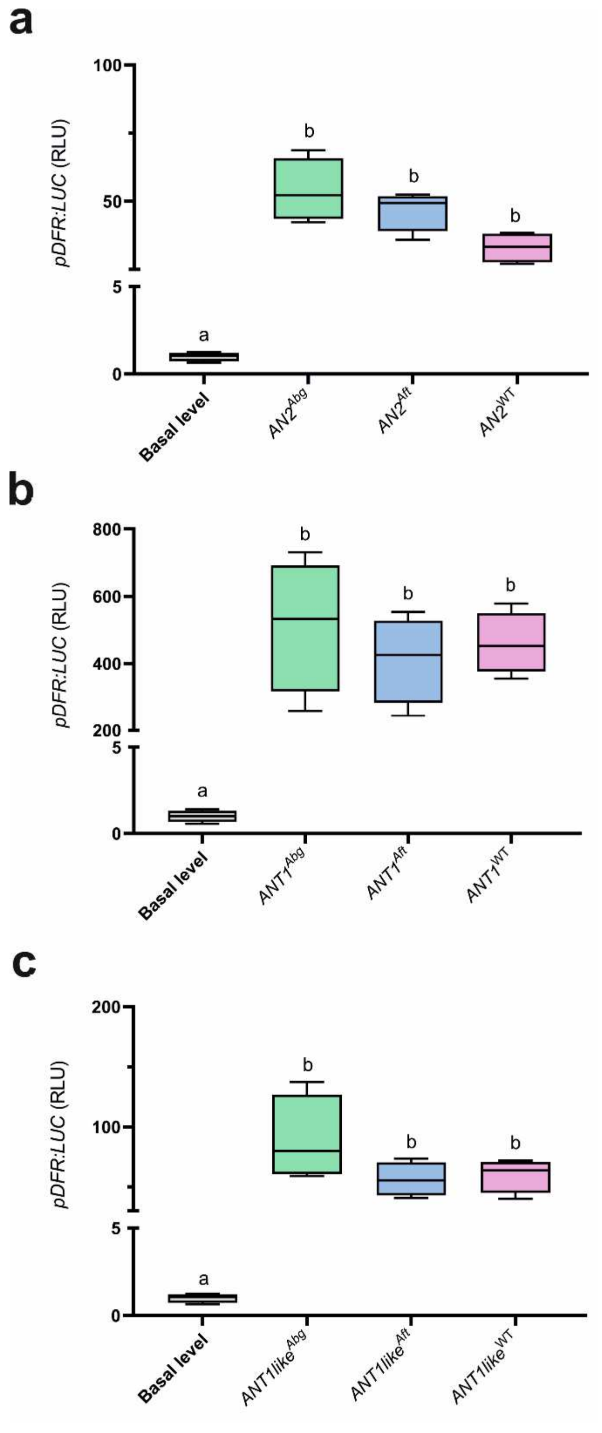

Fig. S18. Dual-Luc assay of the genes *AN2* (**a**), *ANT1* (**b**) and *ANT1like* (**c**) with effector plasmids containing the sequences of the genes of *Aubergine* (Abg), *Anthocyanin fruit* (Aft) and wild type (WT) plants. In all the transactivation assays, the effector *R2R3 MYB* plasmids were co-expressed with the effector plasmid containing the bHLH factor *AN1*. Data are expressed as relative luciferase activity (RLU) (Firefly_Luc/Renilla_Luc) with the value of the *DFR* promoter basal level set to 1 and are means of four biological replicates. One-way ANOVA with Tukey's HSD post hoc test was performed. Different letters indicate significant differences at $P \leq 0.05$.

a

>*AN2like*^{Abg} long transcript
ATGAATATTGCCAAGACATTGGGAGTAAGAAAAGGTTCATGGACTGAAGAAGAAGATACTCTTTTGAGGAAATGTATTAACAAGTATGGAGAAGGAAAGT
GGCATCTTGTTCCTTCTAGAGCTGGTCTAAATCGATGTCGAAAGAGTTGTAGACTGAGGTGGTTGAATTATCTAAGGCCACATATCAAGAGAGGTGACTT
TGCTCCGGATGAAATAGATCTCATTTTAAGACTTCACAAGCTTCTAGGCAATAGGTGGTCACTTATTGCTGGGAGACTTCCAGGAAGAACAGCAAACGAT
GTGAAGAACTATTGGAACACACACCTACACAAGAAGTTATTAATAACTCCTCAGATACAAGAGAATAAGTACAATAATACCCTCAAGATTATCACTGAAA
GCACTATACTACGACCACGACCTCGAACCTTTTCAAGTGAAAATAATATTTCTTGGTGCACTAACAATAGTATGATCACAAACACATTATACAAAGATGA
CGAACAACGTAACAAAGAAATCGTAGTAAATATTTGTGAGAAGCCAACAAGAGAAACTCCGTCATCGTCTATAGACGATGACGGAGTTAAATGGTGTACA
AATTTACTGGAAAATTGGAAAGAATTTGAGGAAGCAGCAGCAGCAGTATTGAGCTTTGAGGAAGAAAATAATTTGTTACCAAATTTGTTGAATGAGGGAA
ATAATTCAACGACCATGCAACATGGAGAAAATGATGACTTTTCAGTTGATATTGACCTATGGAATCTATTTAATTAG

>*AN2like*^{Abg} short transcript
ATGAATATTGCCAAGACATTGGGAGTAAGAAAAGGTTCATGGACTGAAGAAGAAGATACTCTTTTGAGGAAATGTATTAACAAGTATGGAGAAGGAAAGT
GGCATCTTGTTCCTTCTAGAGCTGGTCTAAATCGATGTCGAAAGAGTTGTAGACTGAGGTGGTTGAATTATCTAAGGCCACATATCAAGAGAGGTGACTT
TGCTCCGGATGAAATAGATCTCATTTTAAGACTTCACAAGCTTCTAGGCAATAGGTGGTCACTTATTGCTGGGAGACTTCCAGGAAGAACAGCAAACGAT
GTGAAGAACTATTGGAACACACACCTACACAAGAAGTTATTAATAACTCCTCAGATACAAGAGAATAAGTACAATAATACCCTCAAGATTATCACTGAAA
GCACTATACTACGACCACGACCTCGAACCTTTTCAAGTGAAAATAATATTTCTTGGTGCACTAACAATAGTATGATCACAAACACATTATACAAAGATGA
CGGAGTTAAATGGTGTACAAATTTACTGGAAAATTGGAAAGAATTTGAGGAAGCAGCAGCAGCAGTATTGAGCTTTGAGGAAGAAAATAATTTGTTACCA
AATTTGTTGAATGAGGGAAATAATTCAACGACCATGCAACATGGAGAAAATGATGACTTTTCAGTTGATATTGACCTATGGAATCTATTTAATTAG

b

```
AN2like_gene        ATGAATATTGCCAAGACATTGGGAGTAAGAAAAGGTTCATGGACTGAAGAAGAAGATACT  60
AN2like_long tr.    ATGAATATTGCCAAGACATTGGGAGTAAGAAAAGGTTCATGGACTGAAGAAGAAGATACT  60
AN2like_short tr.   ATGAATATTGCCAAGACATTGGGAGTAAGAAAAGGTTCATGGACTGAAGAAGAAGATACT  60
                    ************************************************************

AN2like_ gene       CTTTTGAGGAAATGTATTAACAAGTATGGAGAAGGAAAGTGGCATCTTGTTCCTTCTAGA  120
AN2like_long tr.    CTTTTGAGGAAATGTATTAACAAGTATGGAGAAGGAAAGTGGCATCTTGTTCCTTCTAGA  120
AN2like_short tr.   CTTTTGAGGAAATGTATTAACAAGTATGGAGAAGGAAAGTGGCATCTTGTTCCTTCTAGA  120
                    ************************************************************

AN2like_ gene       GCTGGTAAAGTGAAATTACGATTTTAATTTTATGAATTTTAAATTTTATGATAATAACTA  180
AN2like_long tr.    GCT---------------------------------------------------------  123
AN2like_short tr.   GCT---------------------------------------------------------  123
                    ***

AN2like_ gene       AGTTTTAAATTTATGTAGATATTAAGTAATAATTTGTTAATGCAAAAAATACTATTTAGA  240
AN2like_long tr.    ------------------------------------------------------------  117
AN2like_short tr.   ------------------------------------------------------------  117

AN2like_ gene       CAAAATCTATTAGATTATACTAAATTTCCTTTTTAAGAAAAGAGAAACTTAACCTTATGT  300
AN2like_long tr.    ------------------------------------------------------------  117
AN2like_short tr.   ------------------------------------------------------------  117

AN2like_ gene       TGTGATAGTGGCGTACGAACTCACAACTCTGGCATGAATAGCATTTCATGCCTCCTTTCT  360
AN2like_long tr.    ------------------------------------------------------------  117
AN2like_short tr.   ------------------------------------------------------------  117

AN2like_ gene       TATTACTGAGTCGTCAATCATTTTCGTTAGGAGTTTACAAGTTAATATAGACATATATTT  420
AN2like_long tr.    ------------------------------------------------------------  117
AN2like_short tr.   ------------------------------------------------------------  117

AN2like_ gene        CTTAATTTTTTAGTCCATATACAATATCTACGAAAAAGTTACTTGATCTGTTCAATCCA  480
AN2like_long tr.     ------------------------------------------------------------ 117
AN2like_short tr.    ------------------------------------------------------------ 117

AN2like_ gene       CAAATCCCACTTACTATTTCATACGAATATATGCAGGTCTAAATCGATGTCGAAAGAGTT  540
AN2like_long tr.    ------------------------------------GGTCTAAATCGATGTCGAAAGAGTT  148
AN2like_short tr.   ------------------------------------GGTCTAAATCGATGTCGAAAGAGTT  148
                                                        ************************

AN2like_ gene       GTAGACTGAGGTGGTTGAATTATCTAAGGCCACATATCAAGAGAGGTGACTTTGCTCCGG  600
AN2like_long tr.    GTAGACTGAGGTGGTTGAATTATCTAAGGCCACATATCAAGAGAGGTGACTTTGCTCCGG  208
AN2like_short tr.   GTAGACTGAGGTGGTTGAATTATCTAAGGCCACATATCAAGAGAGGTGACTTTGCTCCGG  208
                    ************************************************************

AN2like_ gene       ATGAAATAGATCTCATTTTAAGACTTCACAAGCTTCTAGGCAATAGGCAAGTCAAAAAAT  660
AN2like_long tr.    ATGAAATAGATCTCATTTTAAGACTTCACAAGCTTCTAGGCAATAGG-------------  255
AN2like_short tr.   ATGAAATAGATCTCATTTTAAGACTTCACAAGCTTCTAGGCAATAGG-------------  255
                    ***********************************************
```

```
AN2like_ gene        TCGTTAAAAAGTATTCAAAAATTATTATACATATATATTTAGGAAAAGTAATTTTTGACA  720
AN2like_long tr.     ------------------------------------------------------------  286
AN2like_short tr.    ------------------------------------------------------------  286

AN2like_ gene        TATAAATCTGAGTACATACTTCTCTCTCAAATATACTATAGTAAGTGTCTTTGATGCACT  780
AN2like_long tr.     ------------------------------------------------------------  286
AN2like_short tr.    ------------------------------------------------------------  286

AN2like_ gene        CACTATTTTATTTTATTTTTTGGTTTAGGTGGTCACTTATTGCTGGGAGACTTCCAGGAA  836
AN2like_long tr.     -------------------------TAGGTGGTCACTTATTGCTGGGAGACTTCCAGGAA  290
AN2like_short tr.    -------------------------TAGGTGGTCACTTATTGCTGGGAGACTTCCAGGAA  290
                                           ***********************************

AN2like_ gene        GAACAGCAAACGATGTGAAGAACTATTGGAACACACACCTACACAAGAAGTTATTAATAA  896
AN2like_long tr.     GAACAGCAAACGATGTGAAGAACTATTGGAACACACACCTACACAAGAAGTTATTAATAA  350
AN2like_short tr.    GAACAGCAAACGATGTGAAGAACTATTGGAACACACACCTACACAAGAAGTTATTAATAA  350
                     ************************************************************

AN2like_ gene        CTCCTCAGATACAAGAGAATAAGTACAATAATACCCTCAAGATTATCACTGAAAGCACTA  956
AN2like_long tr.     CTCCTCAGATACAAGAGAATAAGTACAATAATACCCTCAAGATTATCACTGAAAGCACTA  410
AN2like_short tr.    CTCCTCAGATACAAGAGAATAAGTACAATAATACCCTCAAGATTATCACTGAAAGCACTA  410
                     ************************************************************

AN2like_ gene        TACTACGACCACGACCTCGAACCTTTTCAAGTGAAAATAATATTTCTTGGTGCACTAACA  1016
AN2like_long tr.     TACTACGACCACGACCTCGAACCTTTTCAAGTGAAAATAATATTTCTTGGTGCACTAACA  470
AN2like_short tr.    TACTACGACCACGACCTCGAACCTTTTCAAGTGAAAATAATATTTCTTGGTGCACTAACA  470
                     ************************************************************

AN2like_ gene        ATAGTATGATCACAAACACATTATACAAAGATGACGAACAACGTAACAAAGAAATCGTAG  1076
AN2like_long tr.     ATAGTATGATCACAAACACATTATACAAAGATGACGAACAACGTAACAAAGAAATCGTAG  530
AN2like_short tr.    ATAGTATGATCACAAACACATTATACAAAGA-----------------------------  501
                     *******************************

AN2like_ gene        TAAATATTTGTGAGAAGCCAACAAGAGAAACTCCGTCATCGTCTATAGACGATGACGGAG  1136
AN2like_long tr.     TAAATATTTGTGAGAAGCCAACAAGAGAAACTCCGTCATCGTCTATAGACGATGACGGAG  590
AN2like_short tr.    ----------------------------------------------------TGACGGAG  509
                                                                         ********

AN2like_ gene        TTAAATGGTGTACAAATTTACTGGAAAATTGGAAAGAATTTGAGGAAGCAGCAGCAGCAG  1196
AN2like_long tr.     TTAAATGGTGTACAAATTTACTGGAAAATTGGAAAGAATTTGAGGAAGCAGCAGCAGCAG  650
AN2like_short tr.    TTAAATGGTGTACAAATTTACTGGAAAATTGGAAAGAATTTGAGGAAGCAGCAGCAGCAG  569
                     ************************************************************

AN2like_ gene        TATTGAGCTTTGAGGAAGAAAATAATTTGTTACCAAATTTGTTGAATGAGGGAAATAATT  1256
AN2like_long tr.     TATTGAGCTTTGAGGAAGAAAATAATTTGTTACCAAATTTGTTGAATGAGGGAAATAATT  710
AN2like_short tr.    TATTGAGCTTTGAGGAAGAAAATAATTTGTTACCAAATTTGTTGAATGAGGGAAATAATT  629
                     ************************************************************

AN2like_ gene        CAACGACCATGCAACATGGAGAAAATGATGACTTTTCAGTTGATATTGACCTATGGAATC  1316
AN2like_long tr.     CAACGACCATGCAACATGGAGAAAATGATGACTTTTCAGTTGATATTGACCTATGGAATC  770
AN2like_short tr.    CAACGACCATGCAACATGGAGAAAATGATGACTTTTCAGTTGATATTGACCTATGGAATC  689
                     ************************************************************

AN2like_ gene        TATTTAATTAG       1327
AN2like_long tr.     TATTTAATTAG       781
AN2like_short tr.    TATTTAATTAG       700
                     ***********
```

c

```
AN2like^Abg long tr.    ATGAATATTGCCAAGACATTGGGAGTAAGAAAAGGTTCATGGACTGAAGAAGAAGATACT
AN2like^Aft             ATGAATATTGCCAAGACATTGGGAGTGAGAAAAGGTTCATGGACTGAAGATGAAGATATT
                        ************************** *********************** ******* *

AN2like^Abg long tr.    CTTTTGAGGAAATGTATTAACAAGTATGGAGAAGGAAAGTGGCATCTTGTTCCTTCTAGA
AN2like^Aft             CTTTTGAGGAAATGTATTGACAAGTATGGAGAAGGAAAGTGGCATCTTGTTCCTTTTAGA
                        ****************** ************************************ ****

AN2like^Abg long tr.    GCTGGTCTAAATCGATGTCGAAAGAGTTGTAGACTGAGGTGGTTGAATTATCTAAGGCCA
AN2like^Aft             GCTGGTCTAAATAGATGTCGAAAGAGTTGTAGACTGAGGTGGTTGAATTATCTAAGGCCA
                        ************ ***********************************************

AN2like^Abg long tr.    CATATCAAGAGAGGTGACTTTGCTCCGGATGAAATAGATCTCATTTTAAGACTTCACAAG
AN2like^Aft             CATATCAAGAGAGGTGACTTTGCTATGGATGAAATAGATCTCATTTTGAGACTTCACAAG
                        ************************  ********************* ************

AN2like^Abg long tr.    CTTCTAGGCAATAGGTGGTCACTTATTGCTGGGAGACTTCCAGGAAGAACAGCAAACGAT
AN2like^Aft             CTTCTAGGCAATAGATGGTCACTTATTGCTGGGAGACTTCCGGGAAGAACAGCAAACGAT
                        ************** ************************** ******************
```

```
AN2like^Abg long tr.    GTGAAGAACTATTGGAACACACACCTACACAAGAAGTTATTAATAACTCCTCAGATACAA
AN2like^Aft             GTGAAAAACTATTGGAACACACACCTACACAAGAAGTTATTAATAACTCCTCAGATACAA
                        ***** ******************************************************

AN2like^Abg long tr.    GAGAATAAGTACAATAATACCCTCAAGATTATCACTGAAAGCACTATACTACGACCACGA
AN2like^Aft             GAGAATAAGTACAATAAAACCCTCAAGATTATCACTGAAAGCACTATACTACGACCACGA
                        ***************** ******************************************

AN2like^Abg long tr.    CC------------TCGAACCTTTTCAAGTGAAAATAATATTTCTTGGTGCACTAACAAT
AN2like^Aft             CCAAGACCTCGACCTCGAACATTCTCAAGTGAAAATAATATTTCTTGGTGCACTAACAAT
                        **            ****** ** ************************************

AN2like^Abg long tr.    AGTATGATCACAAACACATTATACAAAGATGACGAACAACGTAACAAAGAAATCGTAGTA
AN2like^Aft             AGTATGATCACAAACACATTAGACAAAGATGACGAACAACGCAACAAAGAAATCGCAGTA
                        ********************* ******************* ************* ****

AN2like^Abg long tr.    AATATTTGTGAGAAGCCAACAAGAGAAACTCCGTCATCGTCTATAGACGATGACGGAGTT
AN2like^Aft             AATATTTGTGAGAAGCCAACAAGAGAAACACCGTCATCGTCTATAGACGATGATGGAGTT
                        ***************************** *********************** ******

AN2like^Abg long tr.    AAATGGTGTACAAATTTACTGGAAAATTGGAAAGAATTTGAGGAAGCAGCAGCAGCAGTA
AN2like^Aft             AAATGGTGGACAAATTTACTGGAAAATTGGAAAGAATTTGAGGAAGAAGCAACAGCAGTA
                        ******** ************************************* **** ********

AN2like^Abg long tr.    TTGAGCTTTGAGGAAGAAAATAATTTGTTACCAAATTTGTTGAATGAGGGAAATAATTCA
AN2like^Aft             TTGAACTTTGAGGAAGAAAATAAGTTGTTACCAAATTTGTTGTGTGAGGAACATAATTCA
                        **** ****************** ******************  ***** * ********

AN2like^Abg long tr.    ACGACCATGCAACATGGAGAAAATGATGACTTTTCAGTTGATATTGACCTATGGAATCTA
AN2like^Aft             ACAACCATGCAACATGGAGAAAATGATGACTTTTCAGTTGATATTGACCTATGGAATCTA
                        ** *********************************************************

AN2like^Abg long tr.    TTTAATTAG
AN2like^Aft             TTTAATTAG
                        *********
```

d

AN2like^{Abg} long protein
MNIAKTLGVRKGSWTEEEDTLLRKCINKYGEGKWHLVPSRAGLNRCRKSCRLRWLNYLRPHIKRGDFAPDEIDLILRLHKLLGNRWSLIAGRLPGRTAND
VKNYWNTHLHKKLLITPQIQENKYNNTLKIITESTILRPRPRTFSSENNISWCTNNSMITNTLYKDDEQRNKEIVVNICEKPTRETPSSSIDDDGVKWCT
NLLENWKEFEEAAAAVLSFEEENNLLPNLLNEGNNSTTMQHGENDDFSVDIDLWNLFN

AN2like^{Abg} short protein
MNIAKTLGVRKGSWTEEEDTLLRKCINKYGEGKWHLVPSRAGLNRCRKSCRLRWLNYLRPHIKRGDFAPDEIDLILRLHKLLGNRWSLIAGRLPGRTAND
VKNYWNTHLHKKLLITPQIQENKYNNTLKIITESTILRPRPRTFSSENNISWCTNNSMITNTLYKDDGVKWCTNLLENWKEFEEAAAAVLSFEEENNLLP
NLLNEGNNSTTMQHGENDDFSVDIDLWNLFN

Fig. S19. *AN2like* transcripts and relative proteins in *Aubergine* (*Abg*) plants. **a** Sequences of the long and the short *AN2like* transcripts. **b** ClustalW alignment of the gene *AN2like* with the long and the short transcripts. **c** ClustalW alignment of the long transcript of *AN2like*^{Abg} with the transcript of the same gene of *Anthocyanin fruit* (*Aft*) plants. In b and c the symbol * indicates perfect alignment. **d** Sequence of the two polypeptides produced through translation of the long and of the short transcripts. The 27-long amino acidic stretch that is missing in the shorter transcript is represented in yellow in the sequence of the longer one.

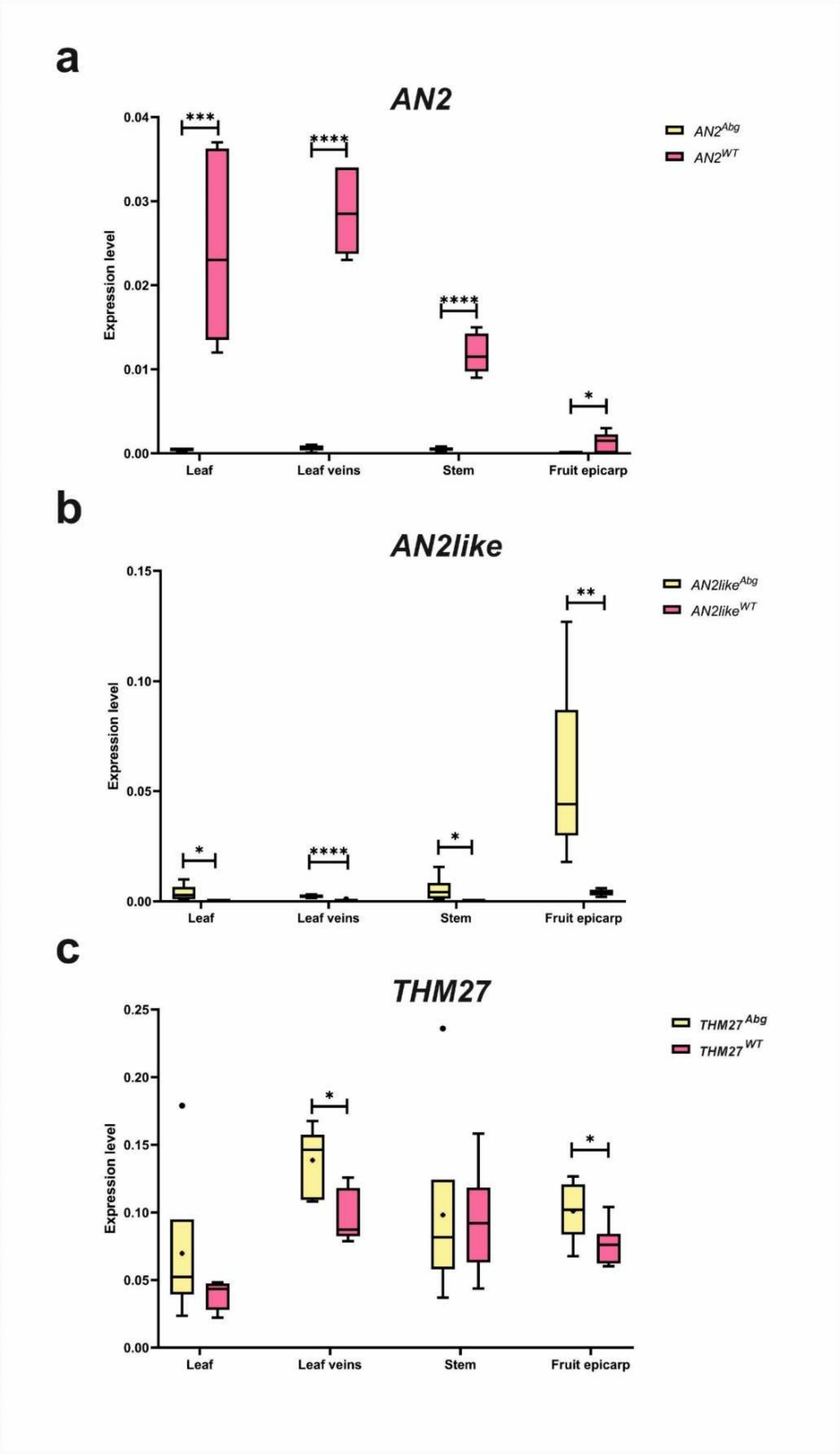

Fig. S20. Expression levels of the wild type (WT) and *Abg* alleles of the regulatory *R2R3 MYB AN2* (**a**), *AN2like* (**b**), and *THM27* (**c**) genes analysed by qPCR in leaves, leaf veins, stems and fruit peel from *Abg* heterozygous (*Abg*/+) plants. Data are means of six biological replicates. Unpaired t-test was carried out for WT and *Abg* alleles for each gene and * ($P \leq 0.05$), ** ($P \leq 0.01$), *** ($P \leq 0.001$), and **** ($P \leq 0.0001$) asterisks indicate significant differences.

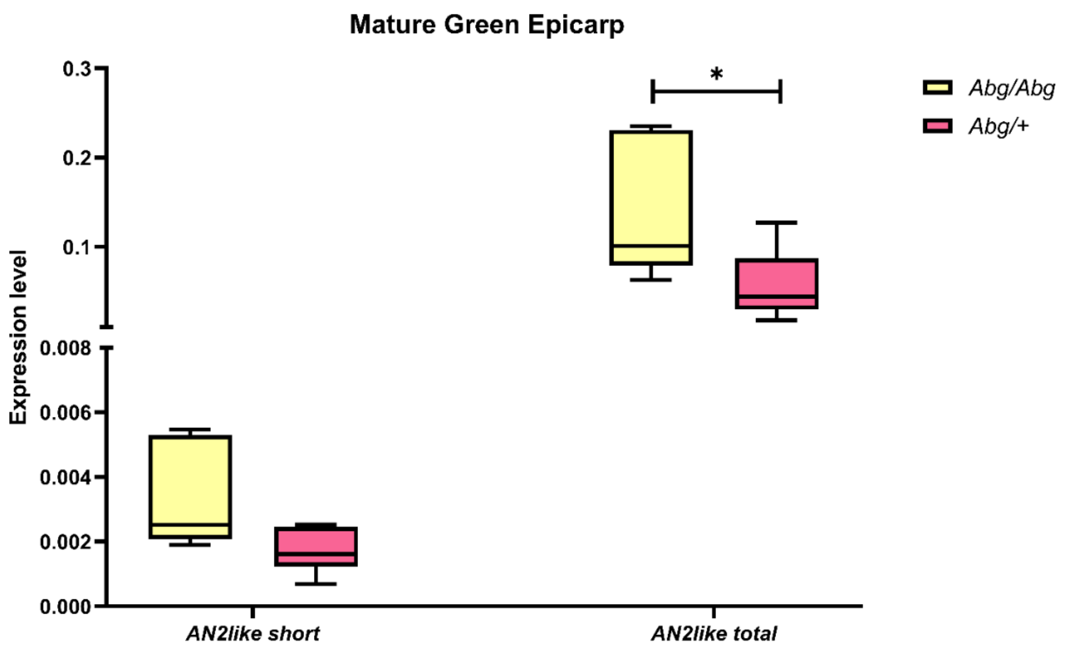

Fig. S21. Expression levels of the total transcripts (long transcript + short transcript) and the short transcripts of the *AN2like*^{Abg} gene analysed by qPCR in the fruit peel of *Abg* homozygous (*Abg*/*Abg*) and heterozygous (*Abg*/+) plants. Data are means of six biological replicates. Unpaired t-test was carried out and * asterisk indicates significant difference ($P \leq 0.05$).

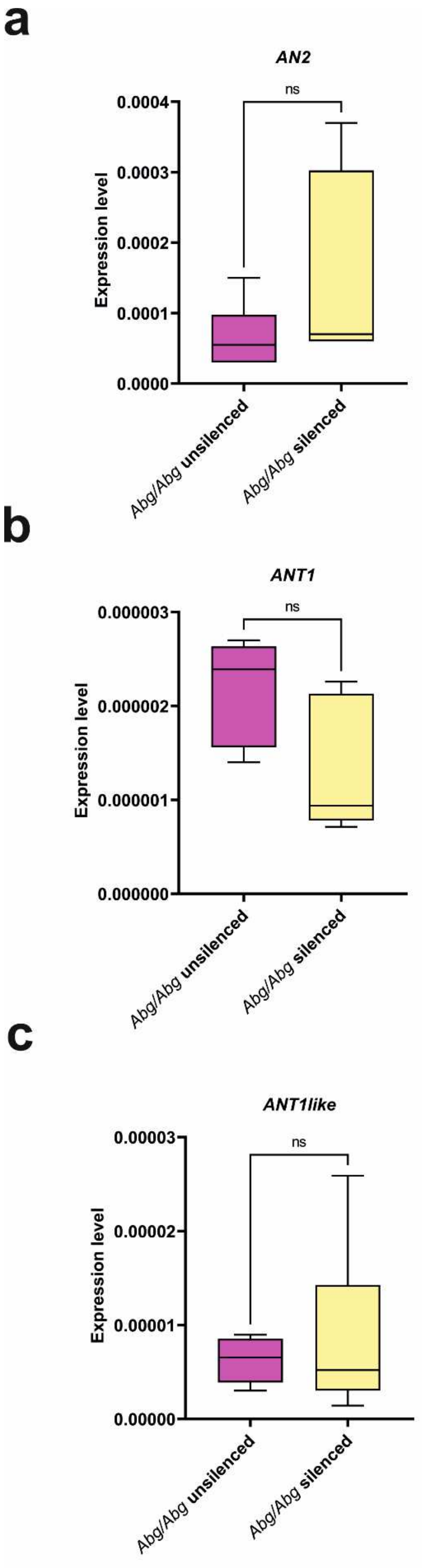

Fig. S22. qPCR analysis of the regulatory *R2R3 MYB* gene *AN2*, *ANT1* and *ANT1like* in the peel of *Abg*/*Abg* unsilenced or silenced fruits. Data are means of six biological replicates. Unpaired t-test ($P \leq 0.05$) was carried out.

a

*Abg*_LA3668

ATGAATACTCCTATGTGTACATCATTAGGAGTAATTAGGAAAGGTTCATGGACTGAACAAGAAGATTTTCACTTGAGAAAATGCATTCAAAAATATGGTG
AAGGAAAGTGGAATCTTGTTCCTTCTAGAGCTGGTAATTAAACGTTATAATACAACTACTATTTTCCGATTCATCGTTCTTTCTTTCTTAAATTTCATGT
TAATACATGCAGGTTTAAATAGATGTCGGAAAAGCTGTAGACTGAGATGGCTAAATTATCTAAGACCACATATCAAAAGAGGTGACTTTGAACCAGATGA
AGTGGATCTCATTTTGAGGCTTCATAAGCTCTTAGGCAACAGGCAATTAATTAGTACTTTATAATATTTAATACTTTAATTTCATATATATTAATTACAC
TCAATTACGTAAATTATATTACTATGTATTACTGTCTATGTCCCTATTTAGTTGTCCACTTTAAGAAATGATACACGTATTAAAATAGCAACAATTAACA
TAGTGAAGTTACAATTTTATCCTTATTAATTATGGTTTCAAAAAGTTTGAATTAAAACTTGAAAATTTTCAAGAAGTTTAAATAAGGGTATAATAGATTA
TTTTTTTTGTCTTTTCTTAATTTGTCAAAATGGACAAGTAAATAGGGACATGTAAAATAGGAAATATGGACAAATAAATAGGAACAAATGGAGTGTATAA
CATGTCCACTCATGTAGTCATGCGGTACTATATGTTTGTAAAATGTGTAATCATATCATCTAAAAATTACGTTATTTTGTGTAGATGGTCACTTGTTGCT
GGTAGACTTCCAGGAAGGACTTGTAAAATGTGTGTAATCATATCATCTAAAAATTACGTTATTTTGTGTAGGTGGTCACTTATTGCTGGTAGACTTCCAG
GAAGGACAGCAAACGATGTGAAAAATTACTGGAATACTCGTCTTCTAAGGAAGTTAAATACTAATATTACAAAGAATGAAATAATAAAACCTCAACCTCG
GACCTTATCATCAAATGCAAAGAATGTTTCTTGGTGCAACAACAAAAGTATGATCACAAACACATTAGACAAAGATGACAAACAACGGAACAAAGAAATC
GTAGTAAATATCTGTGAGAAGCCAACATCATCGTCTATAGACGATGACGGAGTTAAATGGTGGACAAGTTTACTGGAAAATTGTAATGAAATTGAGGATG
AAGAAGCAGCAGTATTGAGCTTTGAAGAAGAAAATAAGTTGTTACCAAATTTGTTGCATGAGGAAAATAATTCACCAATCATGCTACAAGGAGAAAGTGA
TGGTTGGGATATTGACTTATTATGGAATCTACTTAATTAA

*S.lycopersicoides*_LA2951

ATGAATACTCCTATGTGTACATCATTGGGAGTAATTAGGAAAGGTTCATGGACTGAACAAGAAGATTTTCACTTGAGAAAATGCATTCAAAAATATGGTG
AAGGAAAGTGGAATCTTGTTCCTTCTAGAGCTGGTAATTAACGTTATAATACAACTACTATTTTCCGATTCATCGTTCTTTCTTTCTTAAATTTCATGTT
AATACATGCAGGTCTAAATAGATGTCGAAAAAGCTGTAGACTGAGATGGCTAAATTATCTAAGACCACATATCAAAAGAGGTGACTTTGAACCAGATGAA
GTGGATCTCATTTTGAGGCTTCATAAGCTCTTAGGCAACAGGCAATTAATTAGTACTTTATAATATTTAATACTTTAATTTCATATATATTAATTACACT
CAATTACGTAAATTATATTACTATGTATTACTCCCTATGTTCCTATTTAGTTGTCCACTTTAAGAAATGACACACGTATTAAAATAGCAATAATTAACAT
AGTGAAGTTACAATTTTATCCTTATTAATTATGGTTTCAAAAAGGATGAATTAAAACTTAAAAATTTTCAAGAAGTTTAATTAGGGTATAATAGAAAAAA
AAATTGTCTTTTTTTTTATTTGTTAAAATGAACAAGTAAATAGAGACAGCTAAAAGAGGAAACATGGACAAGTAAATAGGGACAGATGGAGTATATAACAT
GTCCACTCATGCGGTACTATTATATGTTGTGAAATGTGTAATCATATCATCTAAAAATTACGTTATTTTGTGTAGATGGTCACTTATTGCTGATAGACTT
CCAGGAAGGACTTGTAAAATGTGTAATCATATCATCTAAAAATTACGTTATTTTGTGTAGGTGGTCACTTATTGCTGGTAGACTTCCAGGAAGGACAGCA
AACGATGTGAAAAATTACTGGAATACTCGTCTTCTAAGGAAGTTAAATACTAATATTACAAAGAATGAAATAATAAAACCTCAACCTCGGACCTTATCAT
CAAATGCAAAGAATGTTTCTTGGTGCAACAACAAAAGTATGATCACAAACACATTAGACAAAGATGACGAACAACGCAACAAAGAAATCGTAGTAAGTAT
TTGTGAGAAGCCAACATCATCGTCTATAGACGATGACGGAGTTGAATGGTGGACAAGTTTACTGGAAAATTGTAATGAAATTGAGGATGAAGAAGCAGCA
GTATTGAGATTTGAAGAAGAAAATAAGTCGTTACCAAATTTGTTGCATGAGGAAAGTAATTCACCAATCATGCTACAAGGAGAAAGTGATGGTTGGGATA
TTGACTTATTATGGAATCTACTTAATTAA

*S.lycopersicum*_LA4345

ATGAATACTCCTATGTGCACATCATTGGGAGTTAGGAAAGGTTCGTGGACTGAACAAGAAGATTTTCACTTGAGAAAATGCATTAAAAAGTATGGTGAAG
GAAAGTGGAATCTTGTTCCTTCTAGAGCTGGTAATTAAACGTTATAATAATACTATTATTTTCCGATTCATCATTCTTTCTTTCTTAAATTTCGTGTTAA
TACATGTGGGTCTAAATAGATGTCGGAAAAGCTGTAGACTGAGATGGCTAAATTATCTACGGCCACATATCAAAGAGAGGTGTCTTTGAACGGGATGAAG
TGGATTTAATTTTGACGCTTCATTAGCTCTTAGGCAACAGGCAATTAATTAGTACTTTATAATGTTTAATACTTTAATCTCATATATATTAGTTACACTC
GATTACATAAATTATACTACTATGTATTATATAACATGTCCGCTCATGCAATACTATATGTTGTAAAATGTATAACCATATCATCGAAAGATTATGTTAT
TTCGTGTAGGTGGACACTTATTGCTGGTAGACTTCCGGGAAGGACAGCAAACGATATAAAAAACTACTGGAATACTCGTCTTCTAAGAAGTTAAATACTA
ATATTACGAATAATGAAATAACAAAACCTCAACCTCAGACGTTATCATCAAATGCAAAGAATGTTTCTTTGTGCAACAACAAAAGTATGATCACAAACAC
ATTAGATAAAGATGATGAACAACGCAACAAAGAAATCGTAGTAAATATTTGGGAGAAGCCAACGGGAGAAACAACAACATTGCCATCGTCTATAGACGAT
GACAGAGTTGAATGGTGGACAAGCTTACTGGAAAATTGTAAAGAAATTGAGGATGAAGAAGCAGTAGTATTGAGCTTTGAAGAAGAAACTAAGTTATTAT
CAAATTTGTTGCATGAGGAAAATAATAATTCACCAATCATGCTACAAGGAGAAAGTCATGGTTGGGATATTGACTTATTATGGAATCTACTTAATTAA

*S.lycopersicum*_LA2838A

ATGAATACTCCTATGTGCACATCATTGGGAGTTAGGAAAGGTTCGTGGACTGAACAAGAAGATTTTCACTTGAGAAAATGCATTAAAAAGTATGGTGAAG
GAAAGTGGAATCTTGTTCCTTCTAGAGCTGGTAATTAAACGTTATAATAATACTATTATTTTCCGATTCATCATTCTTTCTTTCTTAAATTTCGTGTTAA
TACATGTGGGTCTAAATAGATGTCGGAAAAGCTGTAGACTGAGATGGCTAAATTATCTACGCCACATATCAAAGAGAGGTGTCTTTGAACGGGATGAAGT
GGATTTAATTTTGACGCTTCATTAGCTCTTAGGCAACAGGCAATTAATTAGTACTTTATAATGTTTAATACTTTAATCTCATATATATTAGTTACACTCG
ATTACATAAATTATACTACTATGTATTATATAACATGTCCGCTCATGCAATACTATATGTTGTAAAATGTATAACCATATCATCGAAAGATTATGTTATT
TCGTGTAGGTGGACACTTATTGCTGGTAGACTTCCGGGAAGGACAGCAAACGATATAAAAAACTACTGGAATACTCGTCTTCTAAGAAGTTAAATACTAA
TATTACGAATAATGAAATAACAAAACCTCAACCTCAGACGTTATCATCAAATGCAAAGAATGTTTCTTTGTGCAACAACAAAAGTATGATCACAAACACA
TTAGATAAAGATGATGAACAACGCAACAAAGAAATCGTAGTAAATATTTGGGAGAAGCCAACGGGAGAAACAACAACATTGCCATCGTCTATAGACGATG
ACAGAGTTGAATGGTGGACAAGCTTACTGGAAAATTGTAAAGAAATTGAGGATGAAGAAGCAGTAGTATTGAGCTTTGAAGAAGAAACTAAGTTATTATC
AAATTTGTTGCATGAGGAAAATAATAATTCACCAATCATGCTACAAGGAGAAAGTCATGGTTGGGATATTGACTTATTATGGAATCTACTTAATTAA

*Aft*_LA1996

ATGAATACTCCTATGAGCACATCATTGGGAGTTAGGAAAGGTTCGTGGACTGAACAAGAAGATTTTCACTTGAGAAAATGCATTAAAAAAATATGGTGAAA
GAAAGTGGAATCTTGTTCCTTCTAGAGCTAGTAATTAAACGTTATAATAATACTTCTATTTTTCGATTCATTATTCTTTCTTTCTAAATTTCATGTTAA
TACATGCGAATCTAAATAGATGTCGGAAAAGCTGTAGACTGAGATGACTAAATTATCTACGGCCACATATCAAGAGAGGTGACGTTGAACCAGATGAAGT
GGATTTAATTTTGACGCTTCATTAGCTCTTAGGCAACAGGCAATTAATTAGTACTTTATAATGTTTAATACTTTAATCTCATATATATTAGTTACACTCG
ATTATATAAATTATACTACTATGTATTATATAACATGTCCTGCGGTACTACATGTTGTAAAACGTGTAATCATATCATCTAAAGATTATGTTATTTCGTG
TAGGTGGTTACTTATTGCTGATAGACTTTCGGGAAGGATAGCAAACGATATAAAAAACTATTGAAATACTCGTCTTCTAAGGAAGTTAAATACTAATATT
ACAAAGAATGAAATAATAAAACCTCAACCTCGGACCTTATCATCAAATGCAAAGAATGTTTCTTGGTGCAACAACAAAAGTATGATCACAAACACATTAG
ACAAAGATGACAAACAACGGAACAAAGAAATCGTAGTAAATATTTGGGAGAAGCCAACGGGAGAAACAACAACATTGCCATCGTCTATAGACGATGACAG
AGTTGAATGGTGGACAAGTTTACTGGAAAATTGTAAAGAAATTGAGGATGAAGAAGCAGTAGTATTGAGCTTTGAAGAAGAAAATAAGTTATTATCAAAT
TTGTTGCATGAGGAAAATAATAATTCACCAATCATGCTACAAGGAGAAAGTGATGGTTGGGATATTGACTTATTATGGAATCTACTTAATTAA

*S.chilense*_LA3111

ATGAATACTCCTATGAGCACATCATTGGGAGTTAGGAAAGGTTCGTGGACTGAACAAGAAGATTTTCACTTGAGAAAATGCATTAAAAAAATATGGTGAAA
GAAAGTGGAATCTTGTTCCTTCTAGAGCTGGTAATTAAACGTTATAATAATACTTCTATTTTTCGATTCATTATTCTTTCTTTCTAAATTTCATGTTAA
TACATGCGAATCTAAATAGATGTCGGAAAAGCTGTAGACTGAGATGACTAAATTATCTACGGCCACATATCAAGAGAGGTGACGTTGAACCAGATGAAGT

GGATTTAATTTTGACGCTTCATTAGCTCTTAGGCAACAGGCAATTAATTAGTACTTTATAATGTTTAATACTTTAATCTCATATATATTAGTTACACTCG
ATTACATAAATTATACTACTATGTCTTATATAACATGTCCGCTCATGCGATACTATATGTTGTAAAATGTATAACCATATCATCTAAAGATTATGTTATT
TCGTGTAGGTGGACACTTATTGCTGGTAGACTTTCGGGAAGGACAGCAAGCGATATAAAAAACTACTGGAATACTTGTCTTCTAAGGAAGTTGAATACTA
ATATTACGAATAATCAAATAATAAAACCTCAACCTCGAACGTTATCATCAAATGCAAAGAATGTTTCTTTGTGCATCAACAAAAGTATGATCACAAACAC
ATTAGATAAAGATGATGAACAACGCAACAAAGAAACCGTAGTAAATATTTGTGAGAAGCCAACGGGAGAAACAACAACATTGACAGAGTTGAATGGTGGA
CAAGCTTACTGGAAAATTGTAATGAAATTGAGGATGAAGAAGCAGTAGTATTGAGCTTTGAAGAAGAAAATAAGTTATTATCAAATTTGTTGCATGAGGA
AAATAATAATTCACCAATCATGCTACAAGGAGAAAGTGATGGTTGGGATATTGACTTATTATGGAATCTACTTAATTAA

*S.chilense*_LA1930
ATGAATACTCCTATGTGCACATCATTGGGAGCTAGGGAAGGTTCGTGGACTGAACAAGAAGATTTTCACTTAGAGAAAATGCATTAAAAAATATGGTGAA
GGAAAGTGGAATGTTGTTCCTTCTAGAGCTGGTAATTAAACGTTATGATAATACTACTATTTTCCGGTTCATCAATCTTTCTTTCTTAAATTTTATGTTA
ATATATACGGATCTAAATAAATGTCGGAAAAGCTGTAGACTGAGATGACTAAATTATCTACGGCCACATATCAAGAGAGGTGACTTTGAACCAGATGAAG
TGGATTTAATTTTGACGCTTCATTAGCTCTTAGGCAACAGGCAATTAATTAGTACTTTATAATGTTTAATACTTTAATCTCATATATATTAGTTACACTC
GATTACATAAATTATATTACTATGTATTATATAATATGTCCGCTCATGCGGTACTATATGTTGTAAAATGTGTAACCATATCATCTAAAGATTATGTTAT
TTCGTGTAGATACACTTATTGCTGGTAGACTTCCGGGAAGGCCAGCAAACGATATAAAAAACTACTGTCGTCTTCTAAGGAAGTTAAATACTAATATTAC
GAATAATGAAATAATAAAACCTCAACCTCGCGACGTTATCATCAAATGCAAAGAATGTTTCTTTGTGCAACAACAAAAGTATGATCACAAACACATTAGA
TAAAGATGATGAACAACGCAACAAAGAAATCGAAGTAAATATTTGGGAGAAGCCAACGGGAGAAACAACAACATTGCCATCGTCTATAGACGATGGCAGA
GTTGAATGGTGGACAAGCTTACTGGAAAATTGTAATGAAATTGAGGATGAAGAAGCAGTAGTATTGAGCTTTGAAGAAGAAAATAAGTTATTATCAAATT
TGTTGCATGAGGAAAATAATAATTCACCAATCATGCTACAAGGAGAAAGTGATGGTTGGGATATTGACTTATTATGGAATCTACTTAATTAA

*S.pimpinellifolium*_LA1670
ATGAATACTCCTATGTGCACATCATTGGGAGTTAGGAAAGGTTCGTGGACTGAACAAGAAGATTTTCACTCGAGAAAATGCATTAAAAAATATGGTGAAG
GAAAGTGGAATCTTGTTCCTTCTAGAGCTGGTAATTAAACGTTATAATAATACTATTATTTTCCGATTCATCATTCTTTCTTTCTTAAATTTCGTGTTAA
TACATGTGGGTCTAAATAGATGTCGGAAAAGCTGTAGACTGAGATGGCTAAATTATCTACGGCCACATATCAAAGAGAGGTGTCTTTGAACGGGATGAAG
TGGATTTAATTTTGACGCTTCATTAGCTCTTAGGCAACAGGCAATTAATTAGTACTTTATAATGTTTAATACTTTAATCTCATATATATTAGTTACACTC
GATTACATAAATTATACTACTATGTATTATATAACATGTCCGCTCATGCAATACTATATGTTGTAAAATGTATAACCATATCATCTAAAGATTATGTTAT
TTCGTGTAGGTGGACACTTATTGCTGGTAGACTTCCGGGAAGGACAGCAAACGATATAAAAAACTACTGGAATACTCGTCTTCTAAGAAGTTAAATACTA
ATATTACGAATAATGAAATAACAAAACCTCAACCTCGGACGTTATCATCAAATGCAAAGAATGTTTCTTTGTGCAACAACAAAAGTATGATCTCAAACAC
ATTAGATAAAGATGATGAACAACGCAACAAAGAAATCGTAGTAAATATTTGGGAGAAGCCAACGGGAGAAACAACAACATTGCCATCGTCTATAGACGAT
GACAGAGTTGAATGGTGGACAAGCTTACTGGAAAATTGTAAAGAAATTGAGGATGAAGAAGCAGTAGTATTGAGCTTTGAAGAAGAAACTAAGTTATTAT
CAAATTTGTTGCATGAGGAAAATAATAATTCACCAATCATGCTACAAGGAGAAAGTCATGGTTGGGATATTGACTTATTATGGAATCTACTTAATTAA

*S.pennelli*_LA0716
ATGAATACTCCTATGTGCACATCATTGGGAGTAGGAAAGGTTCGTGGACTGAACAAGAAGATTTTCACTTGAGAAAATGCATTAAAAATATGGTGAAGGA
AAGTGGAATCTTGTTCCTTCTAGAGCTGGTAATTAAACGTTATAATAATACTGCTATTTTCCGATTTATCATTCTTTCTTTCTTAAATTTTATGTTAATA
CATGCGAGTCTAAATAGATGTCGGAAAAGCTGTAGACTGAGATGGCTAAATTATCTGCAGCCACATATCAAGAGAGGTGACTTTGAACCAGATGAAGTGG
ATTTAATTTTGACGCTTCATTAGCTCTTAGGCAACAGGCAATTAATTAGTACTTTATAATGTTTAATACTTTAATCTCATATATATTAGTTACACTCGAT
TACATAAATTACATTACTACGTATTATATAACATGTCCGTTCATGCGGTACTATATGTTGTAAAATGTGTAACCATATCATCTAAAGATTATGTTATTTC
GTGTAGGTGGTCACTTATTGCTGGTAGACTTCAGGGAAGGACAACAAACGATATAAAAAACTACTGGATTACTCGTCTTCTAAGGAAGTTAAATACTAAT
ATTACGAAAAATGAAATAATAAAACCTCAACCTCGAACGGTATCATCAAATGCAAAGAATGTTTCTTTGTGCAACAACAAAAGTATGATCACAAACACAT
TAGATAAAAATGATGAACAACGCAACAAAGAAATCGTAGTAAATATTTGTGAGAAGCCAACGGGAGAAACAACAACATTGCCATCGTCTATAGACGATGA
CAGAGTTGAATGGTGGACAAGTTTACTGGAAAATTGTAATGAAATTGAGGATGAAGAAGCAGTAGTATTGAGCTTTGAAGAAGAAACTAAGTTATTATCA
AATTTGTTGCATGACGAAAATAATAATTCACCAATCATGCTACAAGGAGAAAGTGATGGTTGGGATATTGACTTATTATGGGATCTGCTTAATTAA

*S.lycopersicum_var_cerasiforme*_LA1673
ATGAATACTCCTATGTGCACATCATTGGGAGTTAGGAAAGGTTCGTGGACTGAACAAGAAGATTTTCACTCGAGAAAATGCATTAAAAAATATGGTGAAG
GAAAGTGGAATCTTGTTCCTTCTAGAGCTGGTAATTAAACGTTATAATAATACTATTATTTTCCGATTCATCATTCTTTCTTTCTTAAATTTCGTGTTAA
TACATGTGGGTCTAAATAGATGTCGGAAAAGCTGTAGACTGAGATGGCTAAATTATCTACGGCCACATATCAAAGAGAGGTGTCTTTGAACGGGATGAAG
TGGATTTAATTTTGACGCTTCATTAGCTCTTAGGCAACAGGCAATTAATTAGTACTTTATAATGTTTAATACTTTAATCTCATATATATTAGTTACACTC
GATTACATAAATTATACTACTATGTATTATATAACATGTCCGCTCATGCAATACTATATGTTGTAAAATGTATAACCATATCATCTAAAGATTATGTTAT
TTCGTGTAGGTGGACACTTATTGCTGGTAGACTTCCGGGAAGGACAGCAAACGATATAAAAAACTACTGGAATACTCGTCTTCTAAGAAGTTAAATACTA
ATATTACGAATAATGAAATAACAAAACCTCAACCTCGGACGTTATCATCAAATGCAAAGAATGTTTCTTTGTGCAACAACAAAAGTATGATCTCAAACAC
ATTAGATAAAGATGATGAACAACGCAACAAAGAAATCGTAGTAAATATTTGGGAGAAGCCAACGGGAGAAACAACAACATTGCCATCGTCTATAGACGAT
GACAGAGTTGAATGGTGGACAAGCTTACTGGAAAATTGTAAAGAAATTGAGGATGAAGAAGCAGTAGTATTGAGCTTTGAAGAAGAAACTAAGTTATTAT
CAAATTTGTTGCATGAGGAAAATAATAATTCACCAATCATGCTACAAGGAGAAAGTCATGGTTGGGATATTGACTTATTATGGAATCTACTTAATTAA

*S.sitiens*_LA0574
ATGAATACTCCTATGTGTACATCATTGGGAGTAATTAGGAAAGGTTCATGGACTGAACAAGAAGATTTTCACTTGAGAAAATGCATTCAAAAATATGGTG
AAGGAAAGTGGAATCTTGTTCCTTCTAGAGCTGGTAATTAAACGTTATAATACAACTACTATTTTCCGATTCATCGTTCTTTCTTTCTTAAATTTCATGT
TAATACATGCAGGTCTAAATAGATGTCGAAAAAGCTGTAGACTGAGATGGCTAAATTATCTAAGACCACATATCAAAAGAGGTGACTTTGAACCAGATGA
AGTGGATCTCATTTTGAGGCTTCATAAGCTCTTAGGCAACAGGCAATTAATTAGTACTTTATAAGATTTAATACTTTAATTTCATATATATTAATTACAC
TCAATTACGTAAATTATATTACTATGTATTACTCCCTATGTCCCTATTTAGTTGTCCACTTTAAGAAATGATACACGTATTAAAATAGCAACAATTAACA
TAGTGAAGTTATAATTTTATCCTTATTAATTATGGTTTCAAAAAGGATGAATTAAAACTTAGAAATTTTCAAGAAGTTTAAATAAGGATATAATAGATTT
ATTTTTTTTGTCTTTTCTTAATTTGTCAAAATGGACAAGTAAATAGGGACATATAAAATAGAAAATATAGACAAGTAAATAGGAACAGATGGAGTGTATA
ACATGTCCACTCATGTAGTCATGCGGTACTATATGTTTGTAAAATGTGTAATCATATCATCTAAAAATTACGTTATTTTGTGTAGATGGTCACTGTTGCT
GATAGACTTCCAGGAAGGACTTGTAAAATGTGTCATCATATCATCTAAAAATTACGTTATTTTGTGTAGGTGGTCACTTATTGCTGGTAGACTTCCAGGA
AGGACAGCAAACGATGTGAAAAATTACTGGAATACTCGTCTTCTAAGGAAGTTAAATACTAATATTACTAAAAATGAAATAATAAAACCTCAACCTCGGA
CCTTATCATCAAATGCAAAGAATGTTTCTTGGTGCAACAACAAAAGTATGATCACAAACACATTAGACAAAGATGACGACCAACGCGACAAAGAAATCGT
AGTAAATATTTGTGAGAAGCCAACATCATCGTCTATAGACGATGACGGAGTTGAATGGTGGACAAGTTTACTGGAAATTGAGGATGAAGAAGCAGCAGTA
TTGAGCTTTGAAGAAGAAAATAAGTTGTTACCAAATTTGTTGCATGAGGAAAATAATTCACCAATCATGCTACAAGGAGAAAGTGATGGTTGGGATATTG
ACTTATTATGGAATCTACTTAATTAA

b

```
S.chilense_LA1930                       ATGAATACTCCTATGTGCACATCATTGG---GAGCTAGGGAAGGTTCGTGGACTGAACAA      57
S.pennelli_LA0716                       ATGAATACTCCTATGTGCACATCATTGG---GA-GTAGGAAAGGTTCGTGGACTGAACAA      56
S.lycopersicum_LA2838A                  ATGAATACTCCTATGTGCACATCATTGG---GAGTTAGGAAAGGTTCGTGGACTGAACAA      57
S.lycopersicum_LA4345                   ATGAATACTCCTATGTGCACATCATTGG---GAGTTAGGAAAGGTTCGTGGACTGAACAA      57
S.lycopersicum_var_cerasiforme_LA1673   ATGAATACTCCTATGTGCACATCATTGG---GAGTTAGGAAAGGTTCGTGGACTGAACAA      57
S.pimpinellifolium_LA1670               ATGAATACTCCTATGTGCACATCATTGG---GAGTTAGGAAAGGTTCGTGGACTGAACAA      57
Aft_LA1996                              ATGAATACTCCTATGAGCACATCATTGG---GAGTTAGGAAAGGTTCGTGGACTGAACAA      57
S.chilense_LA3111                       ATGAATACTCCTATGAGCACATCATTGG---GAGTTAGGAAAGGTTCGTGGACTGAACAA      57
S.sitiens_LA0574                        ATGAATACTCCTATGTGTACATCATTGGGAGTAATTAGGAAAGGTTCATGGACTGAACAA      60
S.lycopersicoides_LA2951                ATGAATACTCCTATGTGTACATCATTGGGAGTAATTAGGAAAGGTTCATGGACTGAACAA      60
Abg_LA3668                              ATGAATACTCCTATGTGTACATCATTAGGAGTAATTAGGAAAGGTTCATGGACTGAACAA      60
                                        *************** * ******** *    *  **** ******* ************

S.chilense_LA1930                       GAAGATTTTCACTTAGAGAAAATGCATTAAAAAATATGGTGAAGGAAAGTGGAATGTTGT      117
S.pennelli_LA0716                       GAAGATTTTCACTT-GAGAAAATGCATTAA-AAATATGGTGAAGGAAAGTGGAATCTTGT      114
S.lycopersicum_LA2838A                  GAAGATTTTCACTT-GAGAAAATGCATTAAAAAGTATGGTGAAGGAAAGTGGAATCTTGT      116
S.lycopersicum_LA4345                   GAAGATTTTCACTT-GAGAAAATGCATTAAAAAGTATGGTGAAGGAAAGTGGAATCTTGT      116
S.lycopersicum_var_cerasiforme_LA1673   GAAGATTTTCACTC-GAGAAAATGCATTAAAAAATATGGTGAAGGAAAGTGGAATCTTGT      116
S.pimpinellifolium_LA1670               GAAGATTTTCACTC-GAGAAAATGCATTAAAAAATATGGTGAAGGAAAGTGGAATCTTGT      116
Aft_LA1996                              GAAGATTTTCACTT-GAGAAAATGCATTAAAAAATATGGTGAAAGAAAGTGGAATCTTGT      116
S.chilense_LA3111                       GAAGATTTTCACTT-GAGAAAATGCATTAAAAAATATGGTGAAAGAAAGTGGAATCTTGT      116
S.sitiens_LA0574                        GAAGATTTTCA-CTTGAGAAAATGCATTCAAAAATATGGTGAAGGAAAGTGGAATCTTGT      119
S.lycopersicoides_LA2951                GAAGATTTTCA-CTTGAGAAAATGCATTCAAAAATATGGTGAAGGAAAGTGGAATCTTGT      119
Abg_LA3668                              GAAGATTTTCA-CTTGAGAAAATGCATTCAAAAATATGGTGAAGGAAAGTGGAATCTTGT      119
                                        ***********    ************* * ** ********* *********** ****

S.chilense_LA1930                       TCCTTCTAGAGCTGGTAATTAAACGTTATGATAATACTACTATTTTCCGGTTCATCAATC      177
S.pennelli_LA0716                       TCCTTCTAGAGCTGGTAATTAAACGTTATAATAATACTGCTATTTTCCGATTTATCATTC      174
S.lycopersicum_LA2838A                  TCCTTCTAGAGCTGGTAATTAAACGTTATAATAATACTATTATTTTCCGATTCATCATTC      176
S.lycopersicum_LA4345                   TCCTTCTAGAGCTGGTAATTAAACGTTATAATAATACTATTATTTTCCGATTCATCATTC      176
S.lycopersicum_var_cerasiforme_LA1673   TCCTTCTAGAGCTGGTAATTAAACGTTATAATAATACTATTATTTTCCGATTCATCATTC      176
S.pimpinellifolium_LA1670               TCCTTCTAGAGCTGGTAATTAAACGTTATAATAATACTATTATTTTCCGATTCATCATTC      176
Aft_LA1996                              TCCTTCTAGAGCTAGTAATTAAACGTTATAATAATACTTCTATTTTTCGATTCATTATTC      176
S.chilense_LA3111                       TCCTTCTAGAGCTGGTAATTAAACGTTATAATAATACTTCTATTTTTCGATTCATTATTC      176
S.sitiens_LA0574                        TCCTTCTAGAGCTGGTAATTAAACGTTATAATACAACTACTATTTTCCGATTCATCGTTC      179
S.lycopersicoides_LA2951                TCCTTCTAGAGCTGGTAATTA-ACGTTATAATACAACTACTATTTTCCGATTCATCGTTC      178
Abg_LA3668                              TCCTTCTAGAGCTGGTAATTAAACGTTATAATACAACTACTATTTTCCGATTCATCGTTC      179
                                        ************* ******* ******* ***  ***  ****** ** ** **   **

S.chilense_LA1930                       TTTCTTTCTTAAATTTTATGTTAATATATACGGATCTAAATAAATGTCGGAAAAGCTGTA      237
S.pennelli_LA0716                       TTTCTTTCTTAAATTTTATGTTAATACATGCGAGTCTAAATAGATGTCGGAAAAGCTGTA      234
S.lycopersicum_LA2838A                  TTTCTTTCTTAAATTTCGTGTTAATACATGTGGGTCTAAATAGATGTCGGAAAAGCTGTA      236
S.lycopersicum_LA4345                   TTTCTTTCTTAAATTTCGTGTTAATACATGTGGGTCTAAATAGATGTCGGAAAAGCTGTA      236
S.lycopersicum_var_cerasiforme_LA1673   TTTCTTTCTTAAATTTCGTGTTAATACATGTGGGTCTAAATAGATGTCGGAAAAGCTGTA      236
S.pimpinellifolium_LA1670               TTTCTTTCTTAAATTTCGTGTTAATACATGTGGGTCTAAATAGATGTCGGAAAAGCTGTA      236
Aft_LA1996                              TTTCTTTCTTAAATTTCATGTTAATACATGCGAATCTAAATAGATGTCGGAAAAGCTGTA      236
S.chilense_LA3111                       TTTCTTTCTTAAATTTCATGTTAATACATGCGAATCTAAATAGATGTCGGAAAAGCTGTA      236
S.sitiens_LA0574                        TTTCTTTCTTAAATTTCATGTTAATACATGCAGGTCTAAATAGATGTCGAAAAAGCTGTA      239
S.lycopersicoides_LA2951                TTTCTTTCTTAAATTTCATGTTAATACATGCAGGTCTAAATAGATGTCGAAAAAGCTGTA      238
Abg_LA3668                              TTTCTTTCTTAAATTTCATGTTAATACATGCAGGTTTAAATAGATGTCGGAAAAGCTGTA      239
                                        ****************  ******** **     * ****** ****** **********

S.chilense_LA1930                       GACTGAGATGACTAAATTATCTACGGCCACATATCAA-GAGAGGTGACTTTGAACCAGAT      296
S.pennelli_LA0716                       GACTGAGATGGCTAAATTATCTGCAGCCACATATCAA-GAGAGGTGACTTTGAACCAGAT      293
S.lycopersicum_LA2838A                  GACTGAGATGGCTAAATTATCTACG-CCACATATCAAAGAGAGGTGTCTTTGAACGGGAT      295
S.lycopersicum_LA4345                   GACTGAGATGGCTAAATTATCTACGGCCACATATCAAAGAGAGGTGTCTTTGAACGGGAT      296
S.lycopersicum_var_cerasiforme_LA1673   GACTGAGATGGCTAAATTATCTACGGCCACATATCAAAGAGAGGTGTCTTTGAACGGGAT      296
S.pimpinellifolium_LA1670               GACTGAGATGGCTAAATTATCTACGGCCACATATCAAAGAGAGGTGTCTTTGAACGGGAT      296
Aft_LA1996                              GACTGAGATGACTAAATTATCTACGGCCACATATCAA-GAGAGGTGACGTTGAACCAGAT      295
S.chilense_LA3111                       GACTGAGATGACTAAATTATCTACGGCCACATATCAA-GAGAGGTGACGTTGAACCAGAT      295
S.sitiens_LA0574                        GACTGAGATGGCTAAATTATCTAAGACCACATATCAA-AAGAGGTGACTTTGAACCAGAT      298
S.lycopersicoides_LA2951                GACTGAGATGGCTAAATTATCTAAGACCACATATCAA-AAGAGGTGACTTTGAACCAGAT      297
Abg_LA3668                              GACTGAGATGGCTAAATTATCTAAGACCACATATCAA-AAGAGGTGACTTTGAACCAGAT      298
                                        ********** ***********    ***********  ******* * ******  ***

S.chilense_LA1930                       GAAGTGGATTTAATTTTGACGCTTCATTAGCTCTTAGGCAACAGGCAATTAATTAGTACT      356
S.pennelli_LA0716                       GAAGTGGATTTAATTTTGACGCTTCATTAGCTCTTAGGCAACAGGCAATTAATTAGTACT      353
S.lycopersicum_LA2838A                  GAAGTGGATTTAATTTTGACGCTTCATTAGCTCTTAGGCAACAGGCAATTAATTAGTACT      355
S.lycopersicum_LA4345                   GAAGTGGATTTAATTTTGACGCTTCATTAGCTCTTAGGCAACAGGCAATTAATTAGTACT      356
S.lycopersicum_var_cerasiforme_LA1673   GAAGTGGATTTAATTTTGACGCTTCATTAGCTCTTAGGCAACAGGCAATTAATTAGTACT      356
S.pimpinellifolium_LA1670               GAAGTGGATTTAATTTTGACGCTTCATTAGCTCTTAGGCAACAGGCAATTAATTAGTACT      356
Aft_LA1996                              GAAGTGGATTTAATTTTGACGCTTCATTAGCTCTTAGGCAACAGGCAATTAATTAGTACT      355
S.chilense_LA3111                       GAAGTGGATTTAATTTTGACGCTTCATTAGCTCTTAGGCAACAGGCAATTAATTAGTACT      355
S.sitiens_LA0574                        GAAGTGGATCTCATTTTGAGGCTTCATAAGCTCTTAGGCAACAGGCAATTAATTAGTACT      358
S.lycopersicoides_LA2951                GAAGTGGATCTCATTTTGAGGCTTCATAAGCTCTTAGGCAACAGGCAATTAATTAGTACT      357
Abg_LA3668                              GAAGTGGATCTCATTTTGAGGCTTCATAAGCTCTTAGGCAACAGGCAATTAATTAGTACT      358
                                        ********* * ******* ******* ********************************
```

```
S.chilense_LA1930                        TTATAATGTTTAATACTTTAATCTCATATATATTAGTTACACTCGATTACATAAATTATA     416
S.pennelli_LA0716                        TTATAATGTTTAATACTTTAATCTCATATATATTAGTTACACTCGATTACATAAATTACA     413
S.lycopersicum_LA2838A                   TTATAATGTTTAATACTTTAATCTCATATATATTAGTTACACTCGATTACATAAATTATA     415
S.lycopersicum_LA4345                    TTATAATGTTTAATACTTTAATCTCATATATATTAGTTACACTCGATTACATAAATTATA     416
S.lycopersicum_var_cerasiforme_LA1673    TTATAATGTTTAATACTTTAATCTCATATATATTAGTTACACTCGATTACATAAATTATA     416
S.pimpinellifolium_LA1670                TTATAATGTTTAATACTTTAATCTCATATATATTAGTTACACTCGATTACATAAATTATA     416
Aft_LA1996                               TTATAATGTTTAATACTTTAATCTCATATATATTAGTTACACTCGATTATATAAATTATA     415
S.chilense_LA3111                        TTATAATGTTTAATACTTTAATCTCATATATATTAGTTACACTCGATTACATAAATTATA     415
S.sitiens_LA0574                         TTATAAGATTTAATACTTTAATTTCATATATATTAATTACACTCAATTACGTAAATTATA     418
S.lycopersicoides_LA2951                 TTATAATATTTAATACTTTAATTTCATATATATTAATTACACTCAATTACGTAAATTATA     417
Abg_LA3668                               TTATAATATTTAATACTTTAATTTCATATATATTAATTACACTCAATTACGTAAATTATA     418
                                         ******  ************** ************ ******** ****  ******* *

S.chilense_LA1930                        TTACTATGTATTATATAATATG--------------------------------------     438
S.pennelli_LA0716                        TTACTACGTATTATATAACATG--------------------------------------     435
S.lycopersicum_LA2838A                   CTACTATGTATTATATAACATG--------------------------------------     437
S.lycopersicum_LA4345                    CTACTATGTATTATATAACATG--------------------------------------     438
S.lycopersicum_var_cerasiforme_LA1673    CTACTATGTATTATATAACATG--------------------------------------     438
S.pimpinellifolium_LA1670                CTACTATGTATTATATAACATG--------------------------------------     438
Aft_LA1996                               CTACTATGTATTATATAACATG--------------------------------------     437
S.chilense_LA3111                        CTACTATGTCTTATATAACATG--------------------------------------     437
S.sitiens_LA0574                         TTACTATGTATTACTCCCTATGTCCCTATTTAGTTGTCCACTTTAAGAAATGATACACGT     478
S.lycopersicoides_LA2951                 TTACTATGTATTACTCCCTATGTTCCTATTTAGTTGTCCACTTTAAGAAATGACACACGT     477
Abg_LA3668                               TTACTATGTATTACTGTCTATGTCCCTATTTAGTTGTCCACTTTAAGAAATGATACACGT     478
                                          ***** ** ***      ***

S.chilense_LA1930                        ------------------------------------------------------------     438
S.pennelli_LA0716                        ------------------------------------------------------------     435
S.lycopersicum_LA2838A                   ------------------------------------------------------------     437
S.lycopersicum_LA4345                    ------------------------------------------------------------     438
S.lycopersicum_var_cerasiforme_LA1673    ------------------------------------------------------------     438
S.pimpinellifolium_LA1670                ------------------------------------------------------------     438
Aft_LA1996                               ------------------------------------------------------------     437
S.chilense_LA3111                        ------------------------------------------------------------     437
S.sitiens_LA0574                         ATTAAAATAGCAACAATTAACATAGTGAAGTTATAATTTTATCCTTATTAATTATGGTTT     538
S.lycopersicoides_LA2951                 ATTAAAATAGCAATAATTAACATAGTGAAGTTACAATTTTATCCTTATTAATTATGGTTT     537
Abg_LA3668                               ATTAAAATAGCAACAATTAACATAGTGAAGTTACAATTTTATCCTTATTAATTATGGTTT     538

S.chilense_LA1930                        ------------------------------------------------------------     438
S.pennelli_LA0716                        ------------------------------------------------------------     435
S.lycopersicum_LA2838A                   ------------------------------------------------------------     437
S.lycopersicum_LA4345                    ------------------------------------------------------------     438
S.lycopersicum_var_cerasiforme_LA1673    ------------------------------------------------------------     438
S.pimpinellifolium_LA1670                ------------------------------------------------------------     438
Aft_LA1996                               ------------------------------------------------------------     437
S.chilense_LA3111                        ------------------------------------------------------------     437
S.sitiens_LA0574                         CAAAAAGGATGAATTAAAACTTAGAAATTTTCAAGAAGTTTAAATAAGGATATAATAGAT     598
S.lycopersicoides_LA2951                 CAAAAAGGATGAATTAAAACTTAAAAATTTTCAAGAAGTTTAATTAGGGTATAATAG-AA     596
Abg_LA3668                               CAAAAAGTTTGAATTAAAACTTGAAAATTTTCAAGAAGTTTAAATAAGGGTATAATA-GA     597

S.chilense_LA1930                        ------------------------------------------------------------     438
S.pennelli_LA0716                        ------------------------------------------------------------     435
S.lycopersicum_LA2838A                   ------------------------------------------------------------     437
S.lycopersicum_LA4345                    ------------------------------------------------------------     438
S.lycopersicum_var_cerasiforme_LA1673    ------------------------------------------------------------     438
S.pimpinellifolium_LA1670                ------------------------------------------------------------     438
Aft_LA1996                               ------------------------------------------------------------     437
S.chilense_LA3111                        ------------------------------------------------------------     437
S.sitiens_LA0574                         TTATTTTTTTTGTCTTTTCTTAATTTGTCAAAATGGACAAGTAAATAGGGACATATAAAA     658
S.lycopersicoides_LA2951                 AA--AAAAATTGTCTTTTTTTTATTTGTTAAAATGAACAAGTAAATAGAGACAGCTAAAA     654
Abg_LA3668                               TTATTTTTTTTGTCTTTTCTTAATTTGTCAAAATGGACAAGTAAATAGGGACATGTAAAA     657

S.chilense_LA1930                        ------------------------------------------------------------     438
S.pennelli_LA0716                        ------------------------------------------------------------     435
S.lycopersicum_LA2838A                   ------------------------------------------------------------     437
S.lycopersicum_LA4345                    ------------------------------------------------------------     438
S.lycopersicum_var_cerasiforme_LA1673    ------------------------------------------------------------     438
S.pimpinellifolium_LA1670                ------------------------------------------------------------     438
Aft_LA1996                               ------------------------------------------------------------     437
S.chilense_LA3111                        ------------------------------------------------------------     437
S.sitiens_LA0574                         TAGAAAATATAGACAAGTAAATAGGAACAGATGGAGTGTATAACATGTCCACTCATGTAG     718
S.lycopersicoides_LA2951                 GAGGAAACATGGACAAGTAAATAGGGACAGATGGAGTATATAACATGTCCACTCATGCGG     714
Abg_LA3668                               TAGGAAATATGGACAAATAAATAGGAACAAATGGAGTGTATAACATGTCCACTCATGTAG     717

S.chilense_LA1930                        ------------------------------------------------------------     438
S.pennelli_LA0716                        ------------------------------------------------------------     435
S.lycopersicum_LA2838A                   ------------------------------------------------------------     437
S.lycopersicum_LA4345                    ------------------------------------------------------------     438
S.lycopersicum_var_cerasiforme_LA1673    ------------------------------------------------------------     438
S.pimpinellifolium_LA1670                ------------------------------------------------------------     438
Aft_LA1996                               ------------------------------------------------------------     437
S.chilense_LA3111                        ------------------------------------------------------------     437
S.sitiens_LA0574                         TCATGCGGTACTATATGTTTGTAAAATGTGTAATCATATCATCTAAAAATTACGTTATTT     778
S.lycopersicoides_LA2951                 TACTATTAT------ATGTTGTGAAATGTGTAATCATATCATCTAAAAATTACGTTATTT     768
Abg_LA3668                               TCATGCGGTACTATATGTTTGTAAAATGTGTAATCATATCATCTAAAAATTACGTTATTT     777
```

```
S.chilense_LA1930                          -----------------------TCCGCTCATGCGGTACTATATGTTGTAAAATGTGTAA      475
S.pennelli_LA0716                          -----------------------TCCGTTCATGCGGTACTATATGTTGTAAAATGTGTAA      472
S.lycopersicum_LA2838A                     -----------------------TCCGCTCATGCAATACTATATGTTGTAAAATGTATAA      474
S.lycopersicum_LA4345                      -----------------------TCCGCTCATGCAATACTATATGTTGTAAAATGTATAA      475
S.lycopersicum_var_cerasiforme_LA1673      -----------------------TCCGCTCATGCAATACTATATGTTGTAAAATGTATAA      475
S.pimpinellifolium_LA1670                  -----------------------TCCGCTCATGCAATACTATATGTTGTAAAATGTATAA      475
Aft_LA1996                                 -----------------------TC-----CTGCGGTACTACATGTTGTAAAACGTGTAA      469
S.chilense_LA3111                          -----------------------TCCGCTCATGCGATACTATATGTTGTAAAATGTATAA      474
S.sitiens_LA0574                           TGTGTAGATGGTCACTG-TTGCTGATAGACTTCCAGGAAGGACTTGTAAAATG--TGTCA      835
S.lycopersicoides_LA2951                   TGTGTAGATGGTCACTTATTGCTGATAGACTTCCAGGAAGGACTTGTAAAATG--TGTAA      826
Abg_LA3668                                 TGTGTAGATGGTCACTTGTTGCTGGTAGACTTCCAGGAAGGACTTGTAAAATGTGTGTAA      837
                                                                          * *   *     *  *  **    * * *

S.chilense_LA1930                          CCATATCATCTAAAGATTATGTTATTTCGTGTAGATA--CACTTATTGCTGGTAGACTTC      533
S.pennelli_LA0716                          CCATATCATCTAAAGATTATGTTATTTCGTGTAGGTGGTCACTTATTGCTGGTAGACTTC      532
S.lycopersicum_LA2838A                     CCATATCATCGAAAGATTATGTTATTTCGTGTAGGTGGACACTTATTGCTGGTAGACTTC      534
S.lycopersicum_LA4345                      CCATATCATCGAAAGATTATGTTATTTCGTGTAGGTGGACACTTATTGCTGGTAGACTTC      535
S.lycopersicum_var_cerasiforme_LA1673      CCATATCATCTAAAGATTATGTTATTTCGTGTAGGTGGACACTTATTGCTGGTAGACTTC      535
S.pimpinellifolium_LA1670                  CCATATCATCTAAAGATTATGTTATTTCGTGTAGGTGGACACTTATTGCTGGTAGACTTC      535
Aft_LA1996                                 TCATATCATCTAAAGATTATGTTATTTCGTGTAGGTGGTTACTTATTGCTGATAGACTTT      529
S.chilense_LA3111                          CCATATCATCTAAAGATTATGTTATTTCGTGTAGGTGGACACTTATTGCTGGTAGACTTT      534
S.sitiens_LA0574                           TCATATCATCTAAAAATTACGTTATTTTGTGTAGGTGGTCACTTATTGCTGGTAGACTTC      895
S.lycopersicoides_LA2951                   TCATATCATCTAAAAATTACGTTATTTTGTGTAGGTGGTCACTTATTGCTGGTAGACTTC      886
Abg_LA3668                                 TCATATCATCTAAAAATTACGTTATTTTGTGTAGGTGGTCACTTATTGCTGGTAGACTTC      897
                                            ********* *** **** ******* ****** *    *********** *******

S.chilense_LA1930                          CGGGAAGGCCAGCAAACGATATAAAAAACTACTG------TCGTCTTCTAAGGAAGTTAA      587
S.pennelli_LA0716                          AGGGAAGGACAACAAACGATATAAAAAACTACTGGATTACTCGTCTTCTAAGGAAGTTAA      592
S.lycopersicum_LA2838A                     CGGGAAGGACAGCAAACGATATAAAAAACTACTGGAATACTCGTCTTCTAAGA-AGTTAA      593
S.lycopersicum_LA4345                      CGGGAAGGACAGCAAACGATATAAAAAACTACTGGAATACTCGTCTTCTAAGA-AGTTAA      594
S.lycopersicum_var_cerasiforme_LA1673      CGGGAAGGACAGCAAACGATATAAAAAACTACTGGAATACTCGTCTTCTAAGA-AGTTAA      594
S.pimpinellifolium_LA1670                  CGGGAAGGACAGCAAACGATATAAAAAACTACTGGAATACTCGTCTTCTAAGA-AGTTAA      594
Aft_LA1996                                 CGGGAAGGATAGCAAACGATATAAAAAACTATTGAAATACTCGTCTTCTAAGGAAGTTAA      589
S.chilense_LA3111                          CGGGAAGGACAGCAAGCGATATAAAAAACTACTGGAATACTTGTCTTCTAAGGAAGTTGA      594
S.sitiens_LA0574                           CAGGAAGGACAGCAAACGATGTGAAAAATTACTGGAATACTCGTCTTCTAAGGAAGTTAA      955
S.lycopersicoides_LA2951                   CAGGAAGGACAGCAAACGATGTGAAAAATTACTGGAATACTCGTCTTCTAAGGAAGTTAA      946
Abg_LA3668                                 CAGGAAGGACAGCAAACGATGTGAAAAATTACTGGAATACTCGTCTTCTAAGGAAGTTAA      957
                                             ******  * *** **** * ***** ** **      * **********  **** *

S.chilense_LA1930                          ATACTAATATTACGAATAATGAAATAATAAAACCTCAACCTCGCGACGTTATCATCAAAT      647
S.pennelli_LA0716                          ATACTAATATTACGAAAAATGAAATAATAAAACCTCAACCTC-GAACGGTATCATCAAAT      651
S.lycopersicum_LA2838A                     ATACTAATATTACGAATAATGAAATAACAAAACCTCAACCTC-AGACGTTATCATCAAAT      652
S.lycopersicum_LA4345                      ATACTAATATTACGAATAATGAAATAACAAAACCTCAACCTC-AGACGTTATCATCAAAT      653
S.lycopersicum_var_cerasiforme_LA1673      ATACTAATATTACGAATAATGAAATAACAAAACCTCAACCTC-GGACGTTATCATCAAAT      653
S.pimpinellifolium_LA1670                  ATACTAATATTACGAATAATGAAATAACAAAACCTCAACCTC-GGACGTTATCATCAAAT      653
Aft_LA1996                                 ATACTAATATTACAAAGAATGAAATAATAAAACCTCAACCTC-GGACCTTATCATCAAAT      648
S.chilense_LA3111                          ATACTAATATTACGAATAATCAAATAATAAAACCTCAACCTC-GAACGTTATCATCAAAT      653
S.sitiens_LA0574                           ATACTAATATTACTAAAAATGAAATAATAAAACCTCAACCTCG-GACCTTATCATCAAAT      1014
S.lycopersicoides_LA2951                   ATACTAATATTACAAAGAATGAAATAATAAAACCTCAACCTCG-GACCTTATCATCAAAT      1005
Abg_LA3668                                 ATACTAATATTACAAAGAATGAAATAATAAAACCTCAACCTCG-GACCTTATCATCAAAT      1016
                                           ************* ** *** ****** **************   **  ***********

S.chilense_LA1930                          GCAAAGAATGTTTCTTTGTGCAACAACAAAAGTATGATCACAAACACATTAGATAAAGAT      707
S.pennelli_LA0716                          GCAAAGAATGTTTCTTTGTGCAACAACAAAAGTATGATCACAAACACATTAGATAAAAAT      711
S.lycopersicum_LA2838A                     GCAAAGAATGTTTCTTTGTGCAACAACAAAAGTATGATCACAAACACATTAGATAAAGAT      712
S.lycopersicum_LA4345                      GCAAAGAATGTTTCTTTGTGCAACAACAAAAGTATGATCACAAACACATTAGATAAAGAT      713
S.lycopersicum_var_cerasiforme_LA1673      GCAAAGAATGTTTCTTTGTGCAACAACAAAAGTATGATCTCAAACACATTAGATAAAGAT      713
S.pimpinellifolium_LA1670                  GCAAAGAATGTTTCTTTGTGCAACAACAAAAGTATGATCTCAAACACATTAGATAAAGAT      713
Aft_LA1996                                 GCAAAGAATGTTTCTTGGTGCAACAACAAAAGTATGATCACAAACACATTAGACAAAGAT      708
S.chilense_LA3111                          GCAAAGAATGTTTCTTTGTGCATCAACAAAAGTATGATCACAAACACATTAGATAAAGAT      713
S.sitiens_LA0574                           GCAAAGAATGTTTCTTGGTGCAACAACAAAAGTATGATCACAAACACATTAGACAAAGAT      1074
S.lycopersicoides_LA2951                   GCAAAGAATGTTTCTTGGTGCAACAACAAAAGTATGATCACAAACACATTAGACAAAGAT      1065
Abg_LA3668                                 GCAAAGAATGTTTCTTGGTGCAACAACAAAAGTATGATCACAAACACATTAGACAAAGAT      1076
                                           **************** ***** **************** ************* *** **

S.chilense_LA1930                          GATGAACAACGCAACAAAGAAATCGAAGTAAATATTTGGGAGAAGCCAACGGGAGAAACA      767
S.pennelli_LA0716                          GATGAACAACGCAACAAAGAAATCGTAGTAAATATTTGTGAGAAGCCAACGGGAGAAACA      771
S.lycopersicum_LA2838A                     GATGAACAACGCAACAAAGAAATCGTAGTAAATATTTGGGAGAAGCCAACGGGAGAAACA      772
S.lycopersicum_LA4345                      GATGAACAACGCAACAAAGAAATCGTAGTAAATATTTGGGAGAAGCCAACGGGAGAAACA      773
S.lycopersicum_var_cerasiforme_LA1673      GATGAACAACGCAACAAAGAAATCGTAGTAAATATTTGGGAGAAGCCAACGGGAGAAACA      773
S.pimpinellifolium_LA1670                  GATGAACAACGCAACAAAGAAATCGTAGTAAATATTTGGGAGAAGCCAACGGGAGAAACA      773
Aft_LA1996                                 GACAAACAACGGAACAAAGAAATCGTAGTAAATATTTGGGAGAAGCCAACGGGAGAAACA      768
S.chilense_LA3111                          GATGAACAACGCAACAAAGAAACCGTAGTAAATATTTGTGAGAAGCCAACGGGAGAAACA      773
S.sitiens_LA0574                           GACGACCAACGCGACAAAGAAATCGTAGTAAATATTTGTGAGAAGCCAA-----------      1123
S.lycopersicoides_LA2951                   GACGAACAACGCAACAAAGAAATCGTAGTAAGTATTTGTGAGAAGCCAA-----------      1114
Abg_LA3668                                 GACAAACAACGGAACAAAGAAATCGTAGTAAATATCTGTGAGAAGCCAA-----------      1125
                                           **  * *****  ********* ** ***** *** ** **********

S.chilense_LA1930                          ACAACATTGCCATCGTCTATAGACGATGGCAGAGTTGAATGGTGGACAAGCTTACTGGAA      827
S.pennelli_LA0716                          ACAACATTGCCATCGTCTATAGACGATGACAGAGTTGAATGGTGGACAAGTTTACTGGAA      831
S.lycopersicum_LA2838A                     ACAACATTGCCATCGTCTATAGACGATGACAGAGTTGAATGGTGGACAAGCTTACTGGAA      832
S.lycopersicum_LA4345                      ACAACATTGCCATCGTCTATAGACGATGACAGAGTTGAATGGTGGACAAGCTTACTGGAA      833
S.lycopersicum_var_cerasiforme_LA1673      ACAACATTGCCATCGTCTATAGACGATGACAGAGTTGAATGGTGGACAAGCTTACTGGAA      833
S.pimpinellifolium_LA1670                  ACAACATTGCCATCGTCTATAGACGATGACAGAGTTGAATGGTGGACAAGCTTACTGGAA      833
Aft_LA1996                                 ACAACATTGCCATCGTCTATAGACGATGACAGAGTTGAATGGTGGACAAGTTTACTGGAA      828
S.chilense_LA3111                          ACAACATTG-------------------ACAGAGTTGAATGGTGGACAAGCTTACTGGAA      814
S.sitiens_LA0574                           -------CATCATCGTCTATAGACGATGACGGAGTTGAATGGTGGACAAGTTTACTGGAA      1176
S.lycopersicoides_LA2951                   -------CATCATCGTCTATAGACGATGACGGAGTTGAATGGTGGACAAGTTTACTGGAA      1167
Abg_LA3668                                 -------CATCATCGTCTATAGACGATGACGGAGTTAAATGGTGGACAAGTTTACTGGAA      1178
                                                                        * ***** ************* *********
```

```
S.chilense_LA1930                         AATTGTAATGAAATTGAGGATGAAGAAGCAGTAGTATTGAGCTTTGAAGAAGAAAATAAG      887
S.pennelli_LA0716                         AATTGTAATGAAATTGAGGATGAAGAAGCAGTAGTATTGAGCTTTGAAGAAGAAACTAAG      891
S.lycopersicum_LA2838A                    AATTGTAAAGAAATTGAGGATGAAGAAGCAGTAGTATTGAGCTTTGAAGAAGAAACTAAG      892
S.lycopersicum_LA4345                     AATTGTAAAGAAATTGAGGATGAAGAAGCAGTAGTATTGAGCTTTGAAGAAGAAACTAAG      893
S.lycopersicum_var_cerasiforme_LA1673     AATTGTAAAGAAATTGAGGATGAAGAAGCAGTAGTATTGAGCTTTGAAGAAGAAACTAAG      893
S.pimpinellifolium_LA1670                 AATTGTAAAGAAATTGAGGATGAAGAAGCAGTAGTATTGAGCTTTGAAGAAGAAACTAAG      893
Aft_LA1996                                AATTGTAAAGAAATTGAGGATGAAGAAGCAGTAGTATTGAGCTTTGAAGAAGAAAATAAG      888
S.chilense_LA3111                         AATTGTAATGAAATTGAGGATGAAGAAGCAGTAGTATTGAGCTTTGAAGAAGAAAATAAG      874
S.sitiens_LA0574                          ------------ATTGAGGATGAAGAAGCAGCAGTATTGAGCTTTGAAGAAGAAAATAAG      1224
S.lycopersicoides_LA2951                  AATTGTAATGAAATTGAGGATGAAGAAGCAGCAGTATTGAGATTTGAAGAAGAAAATAAG      1227
Abg_LA3668                                AATTGTAATGAAATTGAGGATGAAGAAGCAGCAGTATTGAGCTTTGAAGAAGAAAATAAG      1238
                                                      ******************* ********* ************* ****

S.chilense_LA1930                         TTATTATCAAATTTGTTGCATGAGGAAAATAATAATTCACCAATCATGCTACAAGGAGAA      947
S.pennelli_LA0716                         TTATTATCAAATTTGTTGCATGACGAAAATAATAATTCACCAATCATGCTACAAGGAGAA      951
S.lycopersicum_LA2838A                    TTATTATCAAATTTGTTGCATGAGGAAAATAATAATTCACCAATCATGCTACAAGGAGAA      952
S.lycopersicum_LA4345                     TTATTATCAAATTTGTTGCATGAGGAAAATAATAATTCACCAATCATGCTACAAGGAGAA      953
S.lycopersicum_var_cerasiforme_LA1673     TTATTATCAAATTTGTTGCATGAGGAAAATAATAATTCACCAATCATGCTACAAGGAGAA      953
S.pimpinellifolium_LA1670                 TTATTATCAAATTTGTTGCATGAGGAAAATAATAATTCACCAATCATGCTACAAGGAGAA      953
Aft_LA1996                                TTATTATCAAATTTGTTGCATGAGGAAAATAATAATTCACCAATCATGCTACAAGGAGAA      948
S.chilense_LA3111                         TTATTATCAAATTTGTTGCATGAGGAAAATAATAATTCACCAATCATGCTACAAGGAGAA      934
S.sitiens_LA0574                          TTGTTACCAAATTTGTTGCATGAGGAAAA---TAATTCACCAATCATGCTACAAGGAGAA      1281
S.lycopersicoides_LA2951                  TCGTTACCAAATTTGTTGCATGAGGAAAG---TAATTCACCAATCATGCTACAAGGAGAA      1284
Abg_LA3668                                TTGTTACCAAATTTGTTGCATGAGGAAAA---TAATTCACCAATCATGCTACAAGGAGAA      1295
                                          *  *** **************** ****    ****************************

S.chilense_LA1930                         AGTGATGGTTGGGATATTGACTTATTATGGAATCTACTTAATTAA          992
S.pennelli_LA0716                         AGTGATGGTTGGGATATTGACTTATTATGGGATCTGCTTAATTAA          996
S.lycopersicum_LA2838A                    AGTCATGGTTGGGATATTGACTTATTATGGAATCTACTTAATTAA          997
S.lycopersicum_LA4345                     AGTCATGGTTGGGATATTGACTTATTATGGAATCTACTTAATTAA          998
S.lycopersicum_var_cerasiforme_LA1673     AGTCATGGTTGGGATATTGACTTATTATGGAATCTACTTAATTAA          998
S.pimpinellifolium_LA1670                 AGTCATGGTTGGGATATTGACTTATTATGGAATCTACTTAATTAA          998
Aft_LA1996                                AGTGATGGTTGGGATATTGACTTATTATGGAATCTACTTAATTAA          993
S.chilense_LA3111                         AGTGATGGTTGGGATATTGACTTATTATGGAATCTACTTAATTAA          979
S.sitiens_LA0574                          AGTGATGGTTGGGATATTGACTTATTATGGAATCTACTTAATTAA          1326
S.lycopersicoides_LA2951                  AGTGATGGTTGGGATATTGACTTATTATGGAATCTACTTAATTAA          1329
Abg_LA3668                                AGTGATGGTTGGGATATTGACTTATTATGGAATCTACTTAATTAA          1340
                                          *** ************************** **** *********
```

c

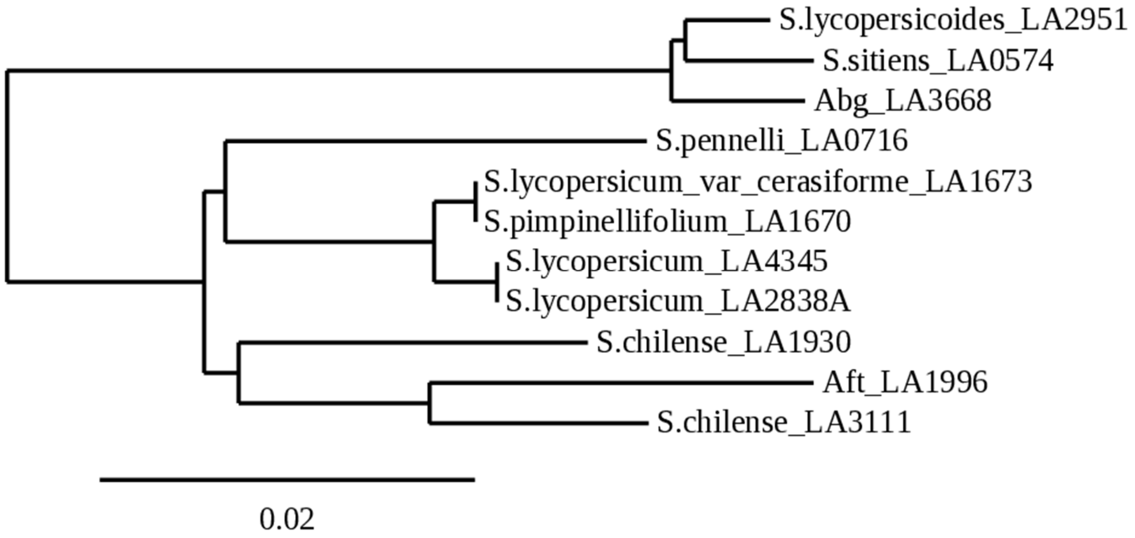

Fig. S23. Sequence analysis of the gene *MYB113* from different tomato accessions and wild Solanum species. **a** Sequence of the gene *MYB113* in tomato (*S. lycopersicum*), *Aubergine* (*Abg*) and in a subset of wild Solanum species. In each sequence exons are highlighted in grey. The stop codons are indicated in red. **b** ClustalW alignment of the gene *MYB113* sequenced in tomato, *Abg* and in a subset of wild Solanum species. The symbol * indicates perfect alignment. **c** Phylogenetic tree of *MYB113*^{Abg} with the sequences of the orthologous genes of tomato and a subset of wild Solanum species. The analysis was performed on the phylogeny.fr platform [54]. LA + number indicates the acronym of the specific accession of each species in which the gene was sequenced.
