# Supplementary material for: Novel R2R3 MYB transcription factors regulate anthocyanin synthesis in Aubergine tomato plants
Source: BMC Plant Biol. 2023 Mar 20;23:148. doi: 10.1186/s12870-023-04153-7 (PMC10026432; doi:10.1186/s12870-023-04153-7)
Supplement: Supplementary file 2 — Additional File 2: Table S1. List of oligonucleotide primers used for gene cloning, CAPS analysis and as VIGS guides. Table S2. List of oligonucleotide primers used for mutagenesis of AN2likeAft. Table S3. List of oligonucleotide primers used for qPCR analysis. [file 12870_2023_4153_MOESM2_ESM.pdf]

Additional File 2

Novel R2R3 MYB transcription factors regulate anthocyanin synthesis in *Aubergine* tomato plants

Authors: Jacopo Menconi, Pierdomenico Perata and Silvia Gonzali*

Affiliations: PlantLab, Center of Plant Sciences, Scuola Superiore Sant'Anna, Piazza Martiri della Libertà 33, 56127 Pisa, Italy

*Author for correspondence: s.gonzali@santannapisa.it (Silvia Gonzali)

This file includes: Tables S1-S3

Table S1. List of oligonucleotide primers used for gene cloning, CAPS analysis and as VIGS guides

Gene Name	Forward primer	Reverse primer
AN2^{Abg}	CACCATGAATACTCCTATGTGTGCCATCGTT	TTAATTAAGTAGATTCCATAAGTCAATATC
ANT1	ATGAACAGTACATCTATGTCTTCA	TTGACTTATGGAATCTACTTGATTAA
ANT1like	ATGAACAGTACATCTATGTCTTC	TGATATTGATATATGGGATTTACTTAATTAA
AN2like	CACCATGAATATTGCCAAGACA	CTAATTAAATAGATTCCATAGGTCAATATC
MYB113	CACCACATATAATGAATACTCCTATG	ATATTAATGACATTAATTAAGTAGATTCC
THM27	CATCATCATCATCATCATCGTCATC	CTCCATCCATTTCCATTACTTTCAC
AN2like CAPS analysis	TCGAAAGAGTTGTAGACTGAGGTG	GTGGTCGTAGTATAGTGCTTTCAGTG
AN2like VIGS guide	CACCGGGAATTCATTGGAACACACACCTA	CGGCTCGAGATTGTTAGTGCACCAAG

Notes: ^{Abg} indicates that the oligonucleotide primers were designed on sequences specific of *Aubergine* gene. When not present, primers were designed on sequences common to *S. lycopersicum* and *S. lycopersicoides*.

Table S2. List of oligonucleotide primers used for mutagenesis of *AN2like*^{Aft}

Primer name	Primer
AN2like^{Aft} *FW overlap*	CACATTAGACAAAGATGATGGAGTTAAATGGTGGACAAA
AN2like^{Aft} *RV overlap*	ATTTAACTCCATCATCTTTGTCTAATGTGTTTGTGATCAT

Table S3. List of oligonucleotide primers used for qPCR analysis

Gene Name	Forward primer	Reverse primer
$AN2^{Abg}$	ACTCTTAGGCAATAGGTGGTCAC	AGGACGAGGATGAAGATGAGGA
$AN2^{WT}$	TTCCAGGAAGGACAGCAAAC	AACGAGGACGAGAATGAGGA
$ANT1^{Abg}$	GCAATGACGATGTTGAAGAAGA	CCCAACTATCATGACTTGTTTG
$ANT1like^{Abg}$	AGAGTAAGCGTGGAGAAATTAGTGA	CCTTACATTGTTCCTCTTTGTCTTC
$AN2like^{Abg}$	GCAAACGATGTGAAGAACTATTGGAAC	GGTCGTAGTATAGTGCTTTCAGTGATAATC
$AN2like^{Abg}$ short	TGAAAGCACTATACTACGACCAC	CACCATTTAACTCCGTCATCTTTGTA
$AN2like^{WT}$	ATATTGCCAAGACATTGGGAGTG	CCATACTTGTCAATACATTTCCTCA
$MYB113^{Abg}$	CCTCAACCTCGGACCTTATCA	CGATGATGTTGGCTTCTCACAG
$THM27^{Abg}$	CATCATCATCATCATCATCGTCATC	CTCCATCCATTTCCATTACTTTCAC
$THM27^{WT}$	ACATCATCAACAACTCGATCATCA	CATCCATTTCCATTACTTTCACTTC
$EF1a^{WT}$	GCTGCTGTAACAAGATGGATGC	GGGGATTTTGTCAGGGTTGTAA
ANS^{WT}	GAACTAGCACTTGGCGTCGAA	TTGCAAGCCAGGCACCATA
DFR^{WT}	TCCGAAGACGACAACGGTTT	TGACAAGCCAAGAGCCGATAA

Notes: ^{Abg} indicates that the oligonucleotide primers were designed on sequences specific of *Aubergine* cds.
^{WT} indicates that the oligonucleotide primers were designed on sequences specific of *S. lycopersicum* cds.
